# The Effect of Dominant Land-Cover Transitions in Shaping Trajectories of Global Forest Change

**DOI:** 10.1007/s00267-025-02245-8

**Published:** 2025-08-05

**Authors:** Aman Gupta, Maarten B. Eppinga, Reinhard Furrer, Maria J. Santos

**Affiliations:** 1 Department of Geography, University of Zurich, Zurich, Switzerland; 2 Department for Mathematical Modeling and Machine Learning, University of Zurich, Zürich, Switzerland

**Keywords:** Global forest change, Forest change trajectories, Land-cover transitions, Landscape configuration, Time-series modeling

## Abstract

Land-cover transitions from forest to non-forest land-covers have led to substantial loss and fragmentation of global forests. While average rates of forest change are well studied, less attention has been paid to spatio-temporal trajectories of forest loss and recovery. These trajectories likely differ by land-cover transition types, and may be constrained by the initial amount and spatial arrangement of the non-forest land-cover. In this study, we distinguish between abrupt and gradual forest change trajectories, evaluating how they vary across land-cover transitions and how initial non-forest land-cover conditions mediate the amount and rate of forest change. To this end, we use annual global land-cover maps (1992–2020) from the European Space Agency’s Climate Change Initiative. We find that the expansion of croplands and bare lands drive forest loss, while transitions from wetlands, shrublands and grasslands are key contributors to forest gain. Forest loss rates were lower when the non-forest land-cover was initially fragmented, except for settlements and bare land, where fragmentation accelerated loss. Conversely, forest gain was highest in wetlands and shrublands, though increased fragmentation generally reduced the amount and rate of forest gain. Our results show that the amount and rate of forest change are associated with the initial amount and spatial arrangement of the non-forest, transition land-cover. This insight may improve our ability to link remotely-sensed land-cover changes to the underlying drivers of global forest change.

## Introduction

Human activities, such as industrial logging, settlement and cropland expansion have contributed to the loss and fragmentation of global forests, with only 18% of global forest area currently intact from signs of human disturbance (IPBES 2019; Watson et al. 2018). Forest fragmentation is a consequence of land-cover (LC) transition between forest and non-forest LCs (Laso Bayas et al. 2022; FAO 2020). LC transitions alter the size, shape and spatial arrangement of forest fragments (Ewers and Didham 2006; Fahrig 2003), with known effects on species composition, tree mortality, carbon storage, and vulnerability to fire, flood, and drought (Sturm et al. 2022; Ordway and Asner 2020; Haddad et al. 2015; Bradshaw et al. 2007; Cochrane 2001). Previous research has highlighted how LC transitions give rise to a variety of spatio-temporal forest change trajectories (Nowosad and Stepinski 2019; Austin et al. 2017; Geist and Lambin 2001). Additionally, the initial amount and spatial arrangement of the non-forest LC can indicate different stages in the process of forest loss or recovery, and influence future trajectories of forest change (Precinoto et al. 2022; van Vliet et al. 2015; Lambin and Geist 2006). Until now, however, forest change assessments have mostly focused on estimates of an average rate of change, while analysis of the variation in spatio-temporal forest change trajectories has received less attention (FAO 2020; Hansen et al. 2013). To address this gap, we calculate a relative rate of forest change to emphasize the difference between gradual and abrupt trajectories of persistent forest loss and gain. Thereafter, we separately assess how the type of LC transition, along with the initial amount and spatial arrangement of the transition LC, mediate the observed amount and rate of forest change. By analyzing these relationships, our study assesses the extent to which pathways of forest loss and recovery are dependent on the dominant LC transition, and the initial composition and configuration of the transition LC.

The amount and rate of forest change are strongly influenced by the type of LC transition. The conversion of forests to agricultural lands, and vice-versa, accounts for 30.6% of all global LC transitions between 1992 and 2018 and is a major driver of global forest dynamics (Potapov et al. 2022; Pendrill et al. 2022; Radwan et al. 2021). Forest to cropland transitions often result in the rapid and extensive clearing of vast tracts of forest areas, especially in tropical regions (Davis et al. 2020; Austin et al. 2017). In contrast, transitions from forests to natural ecosystems, such as grasslands or shrublands, tend to have a reduced extent of impact and also proceed more gradually. These slower transitions are often caused by less-intensive modes of anthropogenic disturbances, such as selective logging, over-grazing or fire, or from climate change induced shifts in local temperature or precipitation patterns (Brando et al. 2019; Stevens et al. 2017). While previous studies have extensively studied the impact of LC transitions on forest extent (Radwan et al. 2021; Mousivand and Arsanjani 2019; Liu et al. 2018), few have investigated their influence on the rate of forest change. Those that have, have typically relied on average estimates of change that are determined by the net difference in forest amount over a time interval (FAO 2020; Austin et al. 2017; Achard et al. 2014; Hansen et al. 2013). Such an approach assumes a uniform rate of change and disregards the diversity of trajectories – from gradual to abrupt - that forest landscapes may potentially undertake during the time frame considered (Ceccherini et al. 2020; Munroe et al. 2013). These trajectories may include critical inflection points that can be useful in delineating distinct phases of forest change (Lambin and Meyfroidt 2011; Rudel et al. 2005). To address this gap, we calculate the relative rate of forest change, defined as the reciprocal of the duration of the interval of forest change, and apply it to forest change trajectories that demonstrate a pattern of persistent forest loss or gain only. While this approach excludes non-persistent forest change trajectories, it effectively discriminates between gradual and abrupt pathways of persistent forest change, offering a refined understanding of how specific drivers shape the temporal dynamics of forest change at the global scale.

The initial amount and spatial arrangement of the transition LC can indicate different stages in the process of forest loss or recovery, influencing future spatio-temporal pathways of forest change (van Vliet et al. 2015; Lambin and Geist 2006). Evidence suggests that LC transitions create distinct patterns of disturbances in forest landscapes (Curtis et al. 2018; Geist and Lambin 2001). For example, large-scale agricultural expansion often results in clear-cut or geometric patterns of deforestation, while smallholder agriculture tends to create patchy or fishbone patterns (Alencar et al. 2023; Austin et al. 2017; Arima et al. 2016; Silva et al. 2008). Consequently, aggregated or compact distributions of croplands in highly disturbed forest landscapes, might indicate the footprint of rapid, commercially-driven deforestation (Nowosad and Stepinski 2019; Austin et al. 2017). In contrast, patchy cropland distributions with interspersed forest cover suggest slower transitions, often due to asynchronous land-use decisions made by multiple landowners (Lambin and Meyfroidt 2011; Rindfuss et al. 2007). Similarly, the potential for forest recovery depends on the size and shape of forest clearings. Fragmented landscapes with small, dispersed forest patches may regenerate slowly due to limited seed dispersal and increased edge effects; while larger, connected forest areas may exhibit greater resilience and capacity for regrowth (Alencar et al. 2023; Munroe et al. 2013). Even urban growth follows patterns of spreading-out (diffusion) and filling-in (coalescence), while also exhibiting an inverse relationship between the size and rate of expansion (Lemoine-Rodríguez et al. 2020; Dietzel et al. 2005). Taken together, these studies highlight the influence of the initial amount and spatial arrangement of the transition LC on the dynamics of forest change.

In this study, we build on past observations that LC transitions give rise to distinct trajectories of forest/non-forest LC arrangements in forested landscapes (Nowosad and Stepinski 2019; Curtis et al. 2018). We operationalize our approach by dividing the global land-surface using a spatial grid of 3 arc-minutes resolution. Within each grid cell, we identify the dominant LC transition contributing to the loss or gain of forest extent, focusing on the period between 1992 and 2020. Thereafter, we assess the varying influence of the type of transition LC (the non-forest LC of the dominant LC transition), as well as its starting amount and spatial arrangement, on the amount and rate of forest change. We hypothesize that the initial amount and spatial arrangement of the transition LC will mediate the amount and rate of observed forest change. More specifically, we ask:

1. Does the amount and rate of forest change vary with the type of transition LC?
2. How does the initial amount and spatial arrangement of the transition LC mediate the observed amount and rate of forest change?

Answering these questions may elucidate how spatio-temporal trajectories of forest change depend on the type of LC transition, and the initial amount and spatial arrangement of the non-forest, transition LC. These insights may improve our ability to link remotely-sensed LC changes to the socio-economic drivers of global forest change.

## Materials and Method

**Fig. 1:**
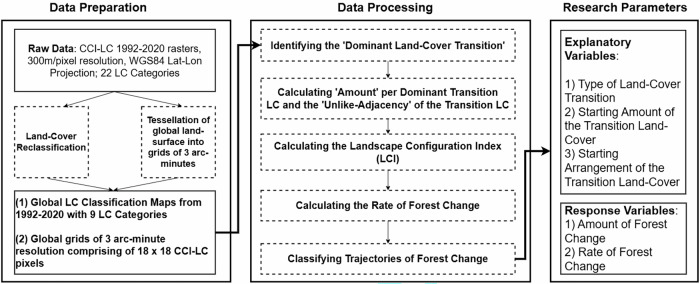
Flowchart illustrating the sequence of steps taken to process the input data in order to capture the relevant research parameters

This section describes the methodological framework for assessing how LC transition type, initial amount and spatial arrangement influence the amount and rate of forest change. Our analysis focuses on grid cells exhibiting substantial (>5% of the grid cell) and persistent forest change (unidirectional loss/gain) over the study period. The method used to extract the relevant research parameters (Fig. 1) and conduct the analysis are described below.

### Data Description

We use the global, annual LC maps spanning 29 years (1992–2020) prepared by the Copernicus Climate Change Service (C3S) and the Climate Change Initiative (CCI) of the European Space Agency (ESA). These maps were created by merging multiple earth observation products based on ESA’s GlobCover project (Defourny et al. 2017), and provide information on 22 distinct LC classes at a horizontal ground-resolution of 300 meters. The typology of LC classes is based on the Land Cover Classification System (LCCS) developed by the United Nations Food and Agricultural Organization (Di Gregorio and Jansen, 2000). The 2015–2020 LC maps have an average overall accuracy of ~71%, with user and producer accuracies for the forest, cropland, bare and water classes exceeding an average of 80% (Defourny et al. 2021; Defourny et al. 2017). However, the data providers note that the validation database was designed to assess static LC classification accuracy and not LC change. They highlight three limitation for LC change detection: (1) changes are captured only between aggregated IPCC land categories, (2) abrupt changes are detected more reliably than gradual ones, and (3) changes are first identified at the 1-km resolution before being downscaled to 300 m. The CCI-LC team acknowledges that accuracy assessments for other years, and detailed validation of LC changes, are currently ongoing using newly collected reference data (Defourny et al. 2021).

### Data Preparation

#### Reclassification

The global LC maps were reclassified into the following IPCC land categories: cropland, forest, grassland, wetland, settlement, shrubland, sparse vegetation, bare area and water, because change detection is only possible between these LC categories (Defourny et al. 2017). ‘Sparse vegetation’ was combined with the ‘bare’ class to further minimize false change detection between semantically similar categories.

#### Tessellation of the global land-surface

We partitioned the global land-surface into mutually exclusive grids of 18 × 18 (=324) CCI-LC pixels, corresponding to a 3 arc-minute spatial resolution. This fixed-area approach facilitates the concurrent spatio-temporal analysis of forest and non-forest LC patterns (Nowosad et al. 2019). While we prioritize the use of the 3 arc-minute resolution to discuss the majority of our results, we additionally repeated our analysis at the 6 and 30 arc-minute resolutions to address potential boundary effects and the modifiable area unit problem (Riitters 2019; Turner and Gardner 2015), thereby measuring the degree to which our findings are robust across scales.

### Data Processing

#### Identifying dominant LC transitions

We calculated the frequency of all LC transitions within each grid cell using global LC maps from 1992 and 2020 only. For each cell, we identified the most frequently occurring LC transition as the dominant LC transition. Forests are the focal LC in this study, therefore we refer to the non-forest class of the dominant LC transition as the ‘transition LC’. For example, in grids where ‘Cropland to Forest’ was the dominant LC transition, cropland is referred to as the ‘transition LC’. We also calculated the percentage of total pixel transitions represented by the dominant transition, which provides a certainty measure for attributing forest change to the identified dominant LC transition. Across 3,006,623 global land-surface grids, forests formed a part of the dominant LC transition in 1,995,644 grids (~66%).

#### Capturing annual amounts of the forest and transition LC

For each LC of the dominant transition, we captured its amount, in number of pixels, for each year spanning 1992 and 2020. We divided these pixel-counts by 324 to express each LC amount as a proportion of the grid size.

#### Calculating the unlike-adjacency of the transition LC

**Fig. 2:**
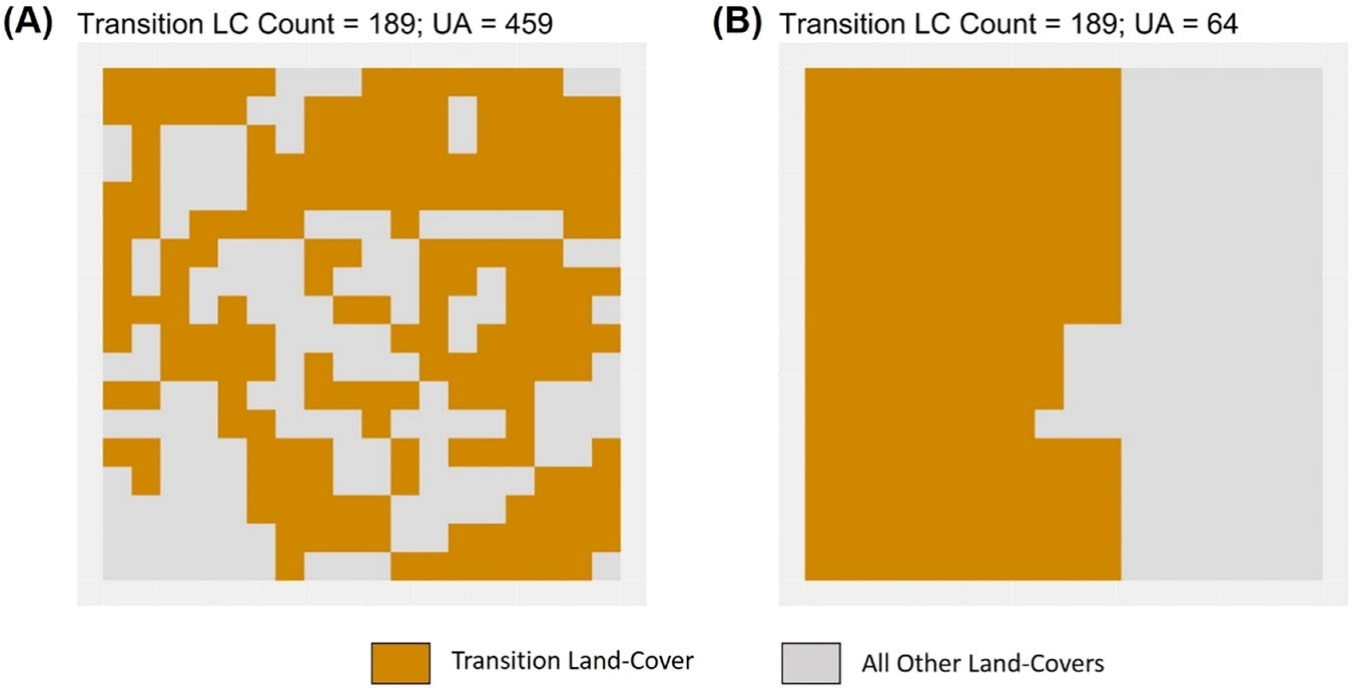
Landscapes showing fragmented (UA = 459; plot (**A**) and aggregated (UA = 64; plot (**B**) spatial arrangements for the same transition land-cover amount (189 pixels)

We quantified the spatial arrangement of the transition LC by calculating the ‘Unlike-Adjacency’ (UA) metric, illustrated in Fig. 2. UA provides a frequency measure of the number of shared edges between pixels of different classes (Turner and Gardner 2015; Hesselbarth et al. 2019); with higher values indicating increased interspersion of the two LCs (Fig. 2A), and lower values reflecting compact spatial arrangements (Fig. 2B). In our study, we calculated the UA between the transition LC and its complementary landscape (i.e. union of all other LC types, including forests) for each year of the study interval. Therefore, each value of UA represents the cumulative sum of the number of other LC types present in the spatial neighborhood of each transition LC pixel. The spatial neighborhood was defined using the Queen’s case of adjacency, with pixels located in the corners, edges and core of the grid cell having 3, 5 and 8 neighbors respectively.

#### Calculating the landscape configuration index (LCI)

**Fig. 3:**
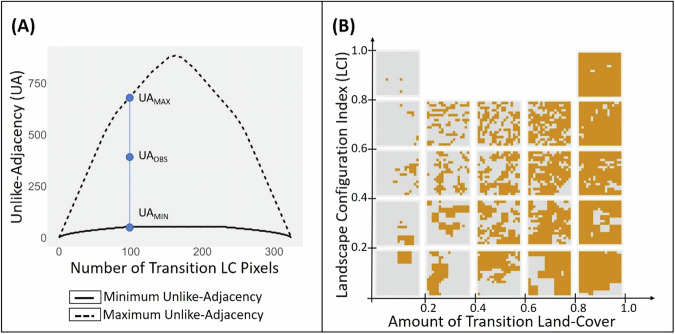
Plot of maximum (UA_max_) and minimum (UA_min_) unlike-adjacency for every possible number of transition LC pixels in the grid cell (**A**); Spatial arrangements of the transition LC for varying starting amounts and LCI values of the transition LC (**B**)

The UA is an effective metric to measure differences in fragmentation patterns that have similar number of transition LC pixels (as illustrated in Fig. 2 above). However, in cases where the number of pixels differ, the use of UA to measure fragmentation can be misleading, as illustrated in Supplementary Fig. 1 of Supplementary Appendix I. To this end, we calculated the theoretical spatial distributions that would maximize and minimize the value of UA for each possible number of transition LC pixels in the grid cell (i.e. 0–324). The method employed to ascertain these theoretical estimates is detailed in Supplementary Appendix II, and the resulting minimal and maximal UA values for the possible range of transition LC pixels is shown in Fig. 3A below.

Therefore, each observed value of UA (UA_obs_) has corresponding maximum (UA_max_) and minimum (UA_min_) UA for every possible number of transition LC pixels. We use these theoretical maximum and minimum UA values to normalize our observed UA value and construct the Landscape Configuration Index (LCI), where LCI = (UA_obs_ – UA_min_)/ (UA_max_ – UA_min_). Consequently, the values of LCI range between 0 and 1. Here, the value of ‘0’ corresponds to a configuration where the transition LC pixels are compactly arranged, while a value of ‘1’ corresponds to a state of maximum fragmentation of the transition LC. We illustrate spatial arrangements of the transition LC for varying values of its starting amount and LCI in Fig. 3B below.

#### Calculating the rate of forest change

To calculate the rate of forest change, we first identified the interval of forest change for each grid cell. The start of this interval was identified as the first year *after* which forest pixels were either lost or gained; while the end of this interval was identified as the last year after which no forest pixels were lost or gained in the grid cell. Subsequently, the rate of forest change was defined as follows:

$${Rate\; of\; Forest\; Change}=\frac{1}{{{{\rm{Duration}}\;{\rm{of}}\;{\rm{the}}\;{\rm{Interval}}\;{\rm{of}}\;{\rm{Forest}}\;{\rm{Change}}}}({\rm{Years}})}$$

This metric represents a relative rate of forest change – as opposed to an absolute rate of change - and was chosen to emphasize differences between gradual and abrupt forest change trajectories. For example, a grid cell losing 30 forest pixels over 30 years and one losing 30 forest pixels in 1 year would yield the same absolute rate of change (1 pixel/year), but would yield drastically different values for the relative rate of forest change (1/30 and 1 year^−1^ respectively).

It is important to note that the amount of forest change was calculated as the difference in forest pixel counts between the start and end of the interval of forest change, while the starting amount and LCI of the transition LC were acquired at the time-step corresponding to the start of the interval of forest change.

#### Classifying trajectories of forest change

**Fig. 4:**
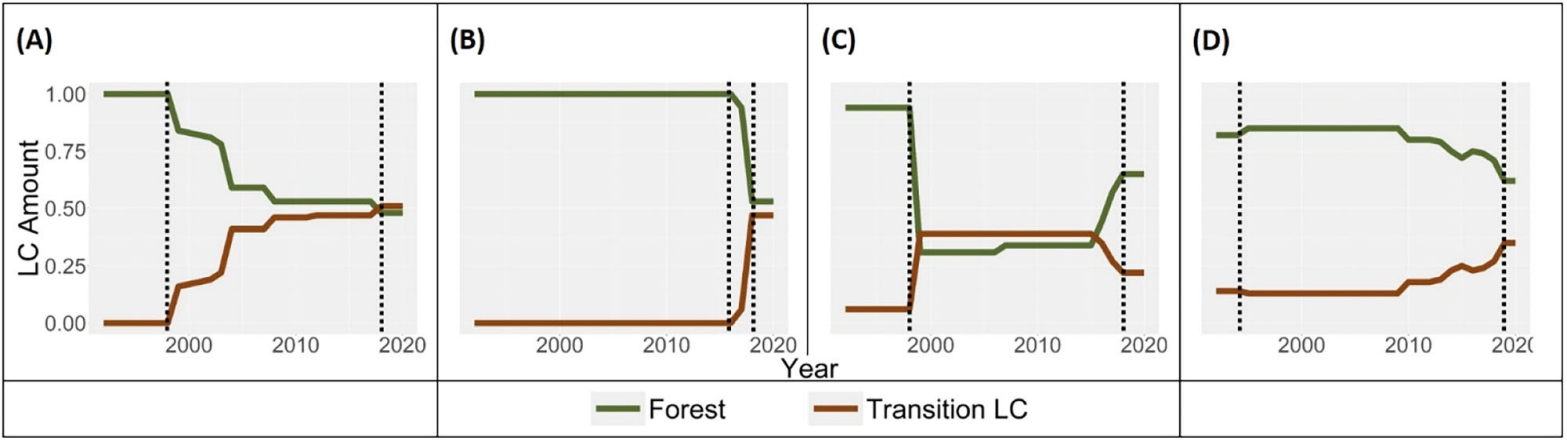
Time-series of forest and transition land-cover (LC) amounts. Plots **A** and **B** depict persistent forest loss showing gradual and abrupt rates of forest loss, respectively. Plots **C** and **D** depict non-persistent forest change, where both forest loss and gain were observed at least once over the interval of forest change. Forest and Transition LC amounts have been expressed as a proportion of the grid size. The interval of forest change has been demarcated using dotted black vertical lines

Based on the time-series of total forest extent in the grid cell, trajectories of forest change were classified as:

1. Persistent Forest Loss or Gain: Trajectories that showed a monotonically decreasing or increasing pattern of total forest extent, i.e., where forest cover changed consistently in one direction (either loss or gain) only (Fig. 4A, B).
2. Non-Persistent Forest Change: Trajectories where both total forest loss and gain were observed at least once during the interval of forest change (Fig. 4C, D).

Our analysis exclusively focuses on grids showing persistent forest loss or gain only. We excluded 370,496 grids (19% of the initial 1,995,644 grids) showing non-persistent forest change because the application of net amount or rate of forest change cannot adequately capture their complex dynamics; and also, because these trajectories typically occur in landscapes where dominant LC transition represents a smaller proportion of total transitions (Supplementary Fig. 2 of Supplementary Appendix I), reducing the certainty of attributing observed forest changes to the dominant LC transition.

#### Additional filters applied to select grids suitable for analysis

We applied the following filters to select grids suitable for our analysis:

1. We excluded 43,294 grids (~2%) where the number of LC pixels was less than 324. These grids were mostly overlying the continental coastline and were removed to ensure that all grids had the same size (see Supplementary Fig. 4 of Supplementary Appendix I).
2. We removed 1,037,035 grids (~52%) where less than 17 forest pixels were lost and gained between 1992 and 2020. This amounts to less than or equal to 5% of forest change. This was done to reduce the likelihood of including misclassified pixels and focus our analysis on landscapes that have experienced a substantial amount of forest change only.
3. We removed 47,323 (~2%) grids for which the transition LC at the start of the interval of forest transition was either absent or occupied the entire grid. These grids were removed because calculating the unlike-adjacency (and the LCI) would not be valid for these cases (see Supplementary Fig. 5 of Supplementary Appendix I).

### Data Analysis

Our analysis focused on 251,322 with persistent forest gain and 246,174 grids persistent forest loss. While net forest change and initial transition LC amounts are expressed as proportions of the grid size, we also provide area estimates in the ‘Discussion and Conclusion’ section to enable a comparison with other studies. To this end, we used the pointDistance function of the ‘raster’ package in ‘R’ to estimate the geographical distance corresponding to the length and width of each grid cell (Hijmans 2024). These estimates were divided by the number of columns and rows of the grid cell to yield the average length and width of the pixels within each grid. These average pixel area estimates were multiplied by the number of LC pixels to estimate the absolute area occupied by a LC within each grid cell.

#### Effects of transition LCs on forest change dynamics

To evaluate differences in the amount and rate of forest loss and gain among LC transition groups, we quantify the absolute difference between each group’s mean and the overall population mean, providing a standardized measure of divergence. We further assess the distributional overlap using Cliff’s Delta (Cliff 1993), a non-parametric effect-size statistic. Cliff’s Delta ranges from −1 to +1, with values near ±1 indicating minimal overlap, and values near 0 suggesting largely overlapping distributions. Group-specific summary statistics are provided in the Results section, while the Cliff’s delta values and confidence intervals are provided in Supplementary Appendix III.

#### Modeling effects on the mean amount and rate of forest change

We used Generalized Additive Models (GAMs) to study the mediating influence of initial amount and spatial arrangement of the transition LC on the mean amount and rate of forest change. GAMs were chosen as they are non-parametric and are better suited to model non-linear patterns (Hastie and Tibshirani 1990). The modeling procedure was implemented using the ‘mgcv’ package in ‘R’ (Wood 2011). The GAM models assumed a Gaussian distribution of the regression errors, and did not affect any transformation of the predicted estimates. The smoothing parameter was estimated using the ‘Restricted Maximum Likelihood’ method, while the smoothing curve was restricted to a maximum of 4 basis functions.

We developed 28 models of forest loss (7 LC classes × 2 predictors × 2 response variables) and 24 for forest gain (6 LC classes × 2 predictors × 2 response variables). For each model, we report the effective degrees of freedom, the proportion of null deviance explained, the values of the initial amount and LCI at which the fitted curves are maximized and minimized, and the mean of the real and absolute values of the average rate of change of the response variable. Here, the mean of the real values of the average rate of change captures the mean effect in the response value to a unit change in the predictor value, while the mean of the absolute values of the average rate of change captures the response sensitivity (measure of magnitude of change regardless of direction) to a unit change in the predictor value. These GAM statistics, and the statistics for the analysis at the 6 and 30 arc-minute resolutions, have been provided in Supplementary Appendix III and IV respectively.

#### Assessment of classification uncertainty

We evaluated how LC classification uncertainty effects our estimates of the amount and rate of forest change. Our analysis indicates that while absolute magnitudes may be affected, the direction and significance of key relationships remain robust. Detailed methods and results of this assessment are provided in Supplementary Appendix V.

## Results

### Effect of the LC Transition on the Amount and Rate of Forest Change

#### Forest loss

**Fig. 5:**
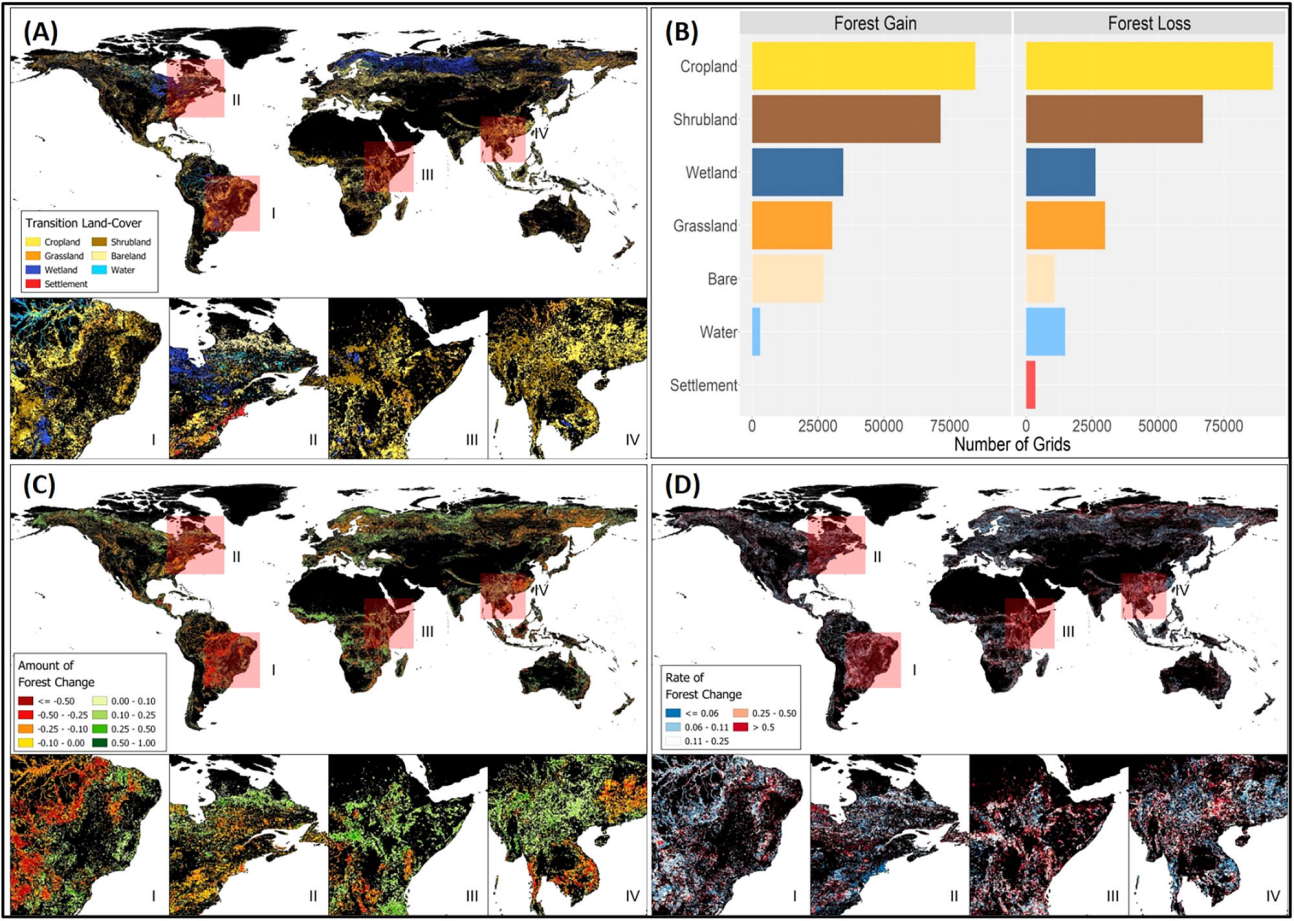
Global maps of the transition land-cover (**A**), the number of transition land-cover grids for forest gain and loss (**B**), amount of forest change (expressed as a proportion of the grid size) (**C**), and the rate of forest change (measured in year^−1^) (D)

Cropland transitions account for 38% of forest loss grids, replacing 20% ± 17% (mean ± standard deviation) of forest cover per grid cell, and exceeding the mean forest loss across all LC transitions by 2.8%. Cliff’s delta revealed small but consistent divergences between cropland and other LCs (mean of absolute Cliff Delta = 0.17). These transitions are prominently found in Central America, Brazilian Amazon, West Africa, South-East Asia and Southeastern China. Shrubland transitions represent 27% of forest loss grids, replacing 17% ± 14% of forest cover per grid cell nearly identical to the population mean (−0.3% deviation). Cliff’s Delta indicates negligible differences in its distribution compared with other LCs (mean of absolute Cliff Delta = 0.08). These transitions are prominent in the Gran Chaco, Miombo woodlands, and boreal regions of North America and Russia. Forest transitions to grasslands represent 12% of all forest loss grids, replacing an average of 16% ± 12% of forest cover per grid cell which is 2% less than the population mean. However, the Cliff’s Delta indicates minimal divergence between grassland and other LCs (mean absolute Cliff Delta = 0.08). In contrast, forest transitions to water and wetlands account for only 17% of all forest loss grids, with water replacing 13% ± 8% and wetlands replacing 14% ± 11% of forest cover per grid cell, and falling 4.6% and 3.9% below the population mean. These transitions cluster in Northern Russia, Scandinavia, Canada, and near major water bodies (e.g., Yacyretá Dam, Tonlé Sap; see Fig. 5; Table 1; Supplementary Table 1 of Supplementary Appendix III).

**Table 1:** Summary statistics for amount of and rate of forest change by transition LC

Amount of forest loss [grid proportion]							
Land-cover	Grid count	Q1	Median	Mean	Q3	SD	Mean diff^a^
Bare	10,770 (0.04)	0.077	0.123	0.165	0.204	0.124	−0.01
Settlement	3484 (0.01)	0.077	0.12	0.151	0.191	0.101	−0.024
Cropland	93,752 (0.38)	0.083	0.139	0.203	0.259	0.17	0.028
Grassland	29,969 (0.12)	0.074	0.114	0.155	0.191	0.118	−0.02
Shrubland	67,101 (0.27)	0.077	0.123	0.172	0.21	0.141	−0.003
Water	14,771 (0.06)	0.074	0.108	0.129	0.16	0.078	−0.046
Wetland	26,327 (0.11)	0.071	0.099	0.136	0.157	0.107	−0.039
**Amount of forest gain [grid proportion]**							
Bare	27,019 (0.11)	0.077	0.12	0.157	0.198	0.114	−0.005
Cropland	84,722 (0.34)	0.077	0.12	0.161	0.204	0.119	−0.001
Grassland	30,413 (0.12)	0.074	0.108	0.148	0.179	0.112	−0.014
Shrubland	71,533 (0.28)	0.077	0.12	0.165	0.21	0.126	0.003
Water	3014 (0.01)	0.077	0.12	0.162	0.204	0.123	0
Wetland	34,621 (0.14)	0.083	0.133	0.176	0.225	0.129	0.014
**Rate of forest loss [year**^**−1**^**]**							
Bare	10,770 (0.04)	0.062	0.143	0.28	0.333	0.309	0.038
Settlement	3484 (0.01)	0.038	0.043	0.053	0.05	0.065	−0.189
Cropland	93,752 (0.38)	0.059	0.1	0.241	0.25	0.294	−0.001
Grassland	29,969 (0.12)	0.059	0.111	0.26	0.333	0.291	0.018
Shrubland	67,101 (0.27)	0.059	0.111	0.245	0.333	0.278	0.003
Water	14,771 (0.06)	0.059	0.083	0.184	0.125	0.263	−0.058
Wetland	26,327 (0.11)	0.062	0.111	0.265	0.333	0.279	0.023
**Rate of forest gain [year**^**−1**^**]**							
Bare	27,019 (0.11)	0.071	0.143	0.307	0.5	0.328	−0.006
Cropland	84,722 (0.34)	0.067	0.167	0.294	0.333	0.319	−0.019
Grassland	30,413 (0.12)	0.083	0.25	0.371	0.5	0.342	0.058
Shrubland	71,533 (0.28)	0.077	0.2	0.329	0.5	0.322	0.016
Water	3014 (0.01)	0.071	0.143	0.278	0.333	0.305	−0.035
Wetland	34,621 (0.14)	0.071	0.143	0.287	0.333	0.312	−0.026

^a^Mean Diff = Land-Cover (LC) Mean – Global Mean for all LCs

Forest to bare transitions account for 4% of forest loss grids, but exhibit the fastest rate of forest loss at 0.28 ± 0.31 year^−1^. These transitions are concentrated in Northern Russia, Scandinavia, and Canada, often in proximity to forest to shrubland transitions. Meanwhile, transitions to water and wetlands show markedly different rates of forest loss, at 0.18 ± 0.26 year^−1^ and 0.27 ± 0.28 year^−1^, respectively. Among all transitions, forest-to-settlement transitions, which account for just 1% of forest loss grids, have the slowest rate of mean forest loss at 0.05 ± 0.07 year^−1^, which are more than 0.189 year^−1^ slower than the population mean. Cliff’s Delta confirmed large divergences in rate of forest loss distributions between settlement and all other LCs (mean absolute Cliff Delta = 0.78). These urban expansions are primarily observed in the Eastern U.S. and parts of Northern Europe (see Fig. 5; Table 1; Supplementary Table 1 of Supplementary Appendix III).

#### Forest gain

Cropland and shrubland transitions account for 34% and 28% of forest gain grids, followed by wetlands (14%), grasslands (12%), bare lands (11%), and water (1%). Croplands have reverted to forests in West Africa, Eastern Tanzania, Southern China, and Eastern Europe. Shrubland transitions to forests are prominent in the African Sahel, the Miombo woodlands of Mozambique, Zimbabwe, Botswana, and Angola, as well as the highlands of Northern Myanmar and Northeast India. Notably, transitions from grasslands yield 15% ± 11% mean forest gain (1.4% below the population mean), while wetland conversions to forests result in a mean forest gain of 18% ± 13% (1.4% above the population mean). Except for the grassland-wetland comparison, Cliff’s Delta indicates negligible distributional differences among all other LC transitions (mean absolute Cliff Delta = 0.03). While the grassland transitions are seen in Northern Russia, Canada and Madagascar, wetland transitions are prominently observed over North-West Russia, Scandinavia, and Canada (see Fig. 5; Table 1; Supplementary Table 2 of Supplementary Appendix III).

Grassland to forest transitions show the fastest rate of forest gain (0.37 ± 0.34 year^−1^), followed by shrubland (0.33 ± 0.32 year^−1^), bare land (0.31 ± 0.33 year^−1^), cropland (0.29 ± 0.32 year^−1^), wetland (0.29 ± 0.31 year^−1^) and water (0.28 ± 0.31 year^−1^). Grassland transitions exceed the population mean rate of gain by 0.06 year^−1^, while water transitions lag by 0.04 year^−1^. The Cliff’s Delta confirms the anomalous speeds associated with the grassland transition, which shows small divergences from the cropland, water and wetland distributions (absolute Cliff’s Delta = 0.17, 0.17 and 0.16, respectively; Table 1; Supplementary Table 2 of Supplementary Appendix III).

### Effect of Initial Amount and Spatial Arrangement on Forest Loss

#### Effect of the initial amount of the transition LC

**Fig. 6:**
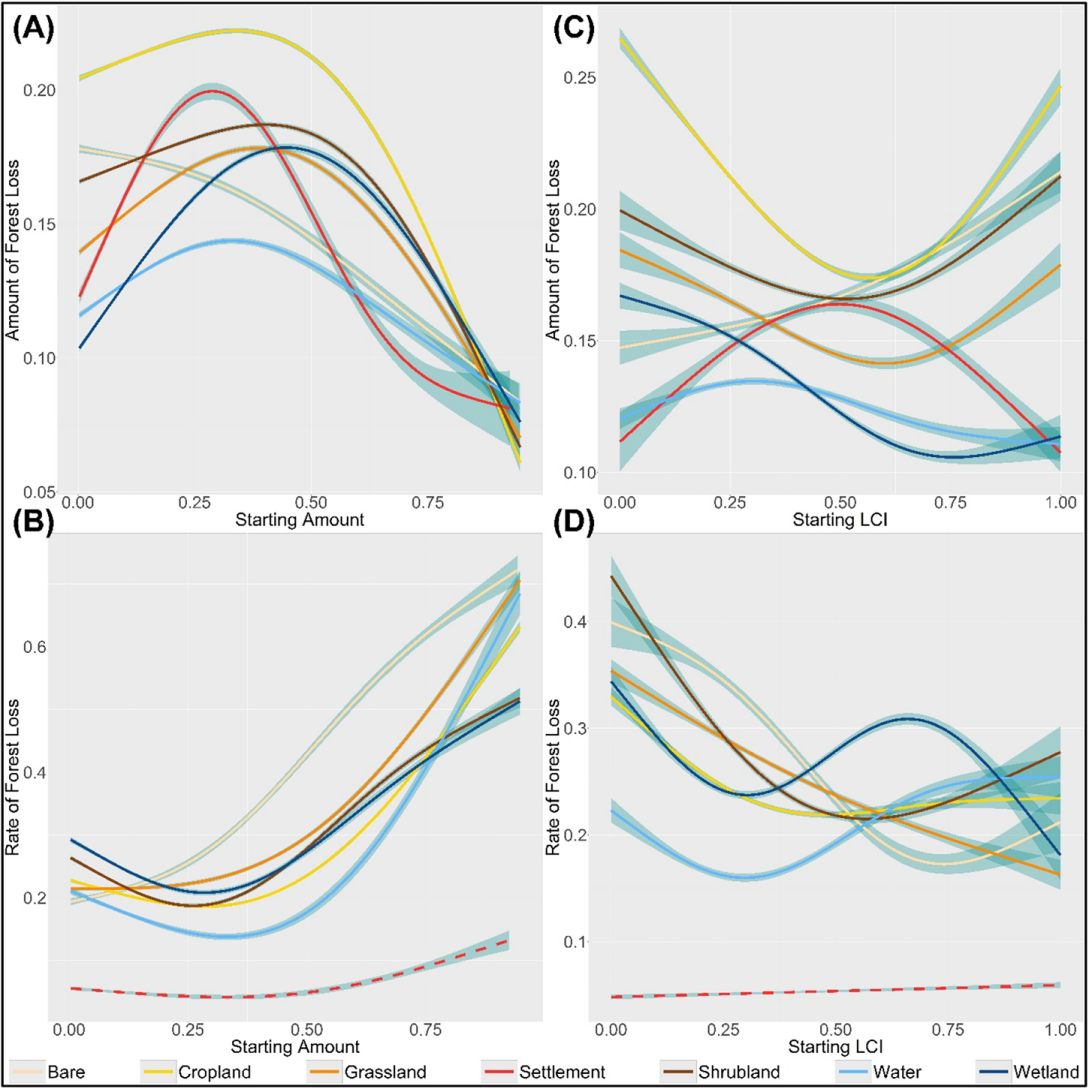
GAMs showing the effect of the starting amount and LCI of each transition LC on the mean amount (plots **A** and **C**, respectively) and rate of forest loss (plots **B** and **D**, respectively). Models with average proportion of null deviance less than 0.2 have been plotted with a dotted line. The blue ribbon around each model curve indicates the uncertainty of the model-fit

Increasing initial amounts of the transition LC are generally associated with a decrease in the mean amount of forest loss (average rate of change = −0.08) and an increase in the mean rate of forest loss (average rate of change = 0.37 year^−1^). These trends remain consistent even as the scale of analysis becomes coarser (see Supplementary Figs. 1 and 2 of Supplementary Appendix IV). Additionally, we find that the mean amount of forest loss peaks at intermediate starting amounts, i.e. when the transition LC occupies ~36% of the grid size (Fig. 6A), while the mean rate of forest loss is minimized at ~30% of the grid size (Fig. 6B). The average proportion of null deviance explained across all LC transitions models testing the effects of the initial amount on the mean amount and rate of forest loss are 0.70 and 0.67 respectively.

We find LC-specific differences in model results. Settlements, croplands, and wetlands show the highest sensitivity of mean forest loss to the changes in the initial amount of the transition LC (mean absolute rates of change = 0.22, 0.19, and 0.19, respectively), while grasslands and shrublands are relatively less sensitive (mean absolute rates of change = 0.16 and 0.15, respectively). For these models, their mean amount of forest loss is maximized when their starting amount occupies between 29% and 44% of the grid size. The bare transition provides the only exception to these patterns, showing an almost linear decrease in mean forest loss for increasing initial amounts (Fig. 6A; Supplementary Table 3 of Supplementary Appendix III). For effects on the mean rate of forest loss, we find that the bare, cropland, and grassland transitions are highly sensitive to changes in their initial amounts (mean absolute rates of change = 0.56, 0.52, and 0.52, respectively), while the shrubland and wetland transitions are relatively less sensitive (mean absolute rates of change = 0.43 and 0.41, respectively). For these transitions, the initial amount at which the mean rate of forest loss is minimized ranges between 26% and 33% of the grid size respectively (Fig. 6B; Supplementary Table 4 of Supplementary Appendix III). However, at the coarsest scale of analysis, i.e. at the 30 arc-minute resolution, the sensitivity of the mean rate of forest loss due to the wetland and shrubland transitions substantially increases (mean absolute rate of change goes from 0.43 to 0.93, and from 0.41 to 3.79 for transitions respectively; Supplementary Table 2 of Supplementary Appendix IV).

#### Effect of the initial spatial arrangement of the transition LC

The initial spatial arrangement varies according to the corresponding initial amount of the transition LC as the complementary portion of the grid delineates a hard limit on the amount of forest loss possible in the landscape. To this end, we provide boxplots depicting the distribution of the initial amount of the transition LC for different levels of LCI (see Supplementary Fig. 1 of Supplementary Appendix III). We observe that in a majority of transition LCs, LCI > 0.6 occurs predominantly when the transition LC occupies less than 12.5% of the grid cell. Therefore, we restrict the scope of our interpretation to LCI < = 0.6.

We find varying effects of the initial LCI on the mean amount of forest loss. For instance, for the cropland, grassland, shrubland and wetland transitions, we find that the mean amount of forest loss decreases with increasing fragmentation of the transition LC (mean rates of change = −0.15, −0.07, −0.05, and −0.09, respectively), while in case of the forest-to-bare and forest-to-settlement transitions increased fragmentation increases the mean amount of forest loss (mean rates of change = 0.04 and 0.08, respectively). Amongst these transitions, however, the cropland, settlement and wetland transitions indicate the maximum sensitivity to changes in their initial LCI (mean of absolute rates of change = 0.15, 0.09 and 0.09 respectively; Fig. 6C; Supplementary Table 4 of Supplementary Appendix III). While these patterns persist, at coarser scales, for most LC transitions, settlement and shrubland transitions exhibit divergent trends at the coarsest resolution (30 arc-minutes), with mean forest loss decreasing with increased fragmentation for settlements, while increasing in the case of shrublands (Fig. 3 and Supplementary Table 3 of Supplementary Appendix IV).

On the other hand, increased fragmentation generally reduces the mean rate of forest loss across all LC transitions (average rate of change = −0.16 year^−1^). The bare and shrubland transitions are most sensitive to changes in their initial LCI (mean absolute rates of change = 0.34 and 0.38, respectively), while the cropland transition is least sensitive (mean absolute rate of change = 0.19). Settlements show negligible sensitivity to their initial spatial arrangement in terms of the rate of forest loss (deviance explained = 0.06; Fig. 6D; Supplementary Table 5 of Supplementary Appendix III). The average proportion of null deviance explained across all LC transitions models testing the effects of the initial LCI on the mean amount and rate of forest loss are 0.54 and 0.56 respectively. Notably, these patterns reverse at coarser resolutions, with forest loss rates generally increasing with increasing fragmentation of the transition LC at the 30 arc-minute resolution (Fig. 4 and Supplementary Table 4 of Supplementary Appendix IV).

### Effect of Initial Amount and Spatial Arrangement on Forest Gain

#### Effect of the initial amount of the transition LC

**Fig. 7:**
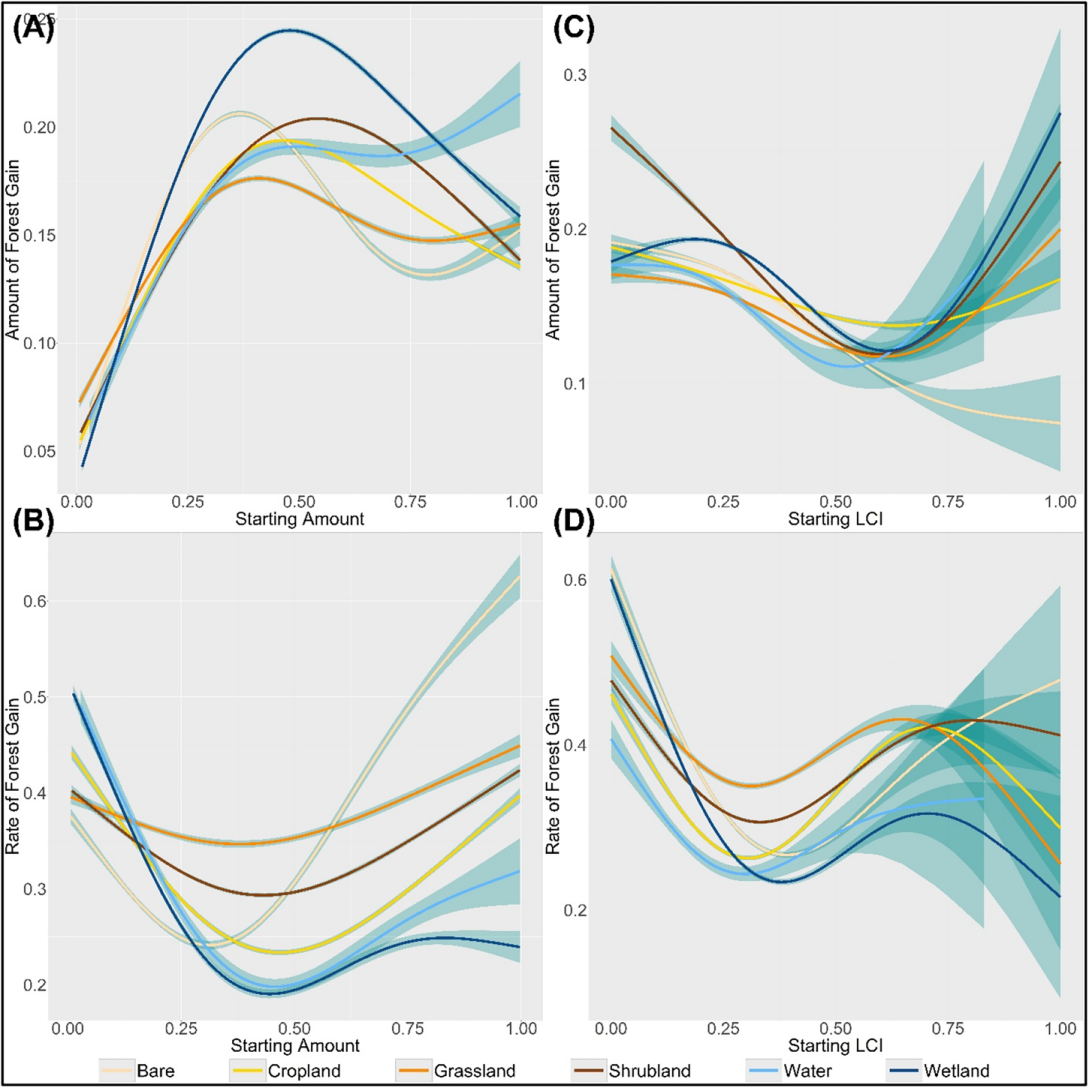
GAMs showing the effect of the starting amount and LCI of each transition LC on the mean amount (plots **A** and **B**, respectively) and rate of forest gain (plots **C** and **D**, respectively). The blue ribbon indicates the uncertainty of the model-fit

Increasing initial amounts of the transition LC generally lead to an increase in the mean amount of forest gain (average rate of change = 0.10) but a decrease in the mean rate of forest gain (average rate of change = −0.03 year^−1^; Fig. 7A, B, respectively). These trends remain consistent at coarser scales of analysis as well (Supplementary Tables 5 and 6 and Supplementary Figs. 5 and 6 of Supplementary Appendix IV). We find evidence indicating the influence of the initial amount of all transition LCs on the mean amount and rate of forest gain (average proportion of null deviance explained = 0.81 and 0.58, respectively).

We find LC-specific differences in model results. The bare, shrubland, and wetland transitions show the highest sensitivity of mean amount of forest gain to changes in their initial amounts (mean absolute rates of change = 0.25, 0.21, and 0.29, respectively), while the grassland and cropland transitions are relatively less sensitive (mean absolute rates of change = 0.14 and 0.20, respectively). For these LC transitions, the initial amount at which the mean amount of forest gain is maximized ranges from 37% for bare to 54% for shrublands. The model for the water-to-forest transition has the lowest value of explained deviance (0.43), suggesting a weaker model fit (Fig. 7A; Supplementary Table 6 of Supplementary Appendix III). The effects of the initial amount on the mean rate of forest gain vary more widely across LC transitions. The bare transition shows the highest sensitivity (mean absolute rate of change = 0.52), with a strong positive trend (average rate of change = 0.26 year^−1^), while the cropland, water, and wetland transitions show negative average rates of change (−0.04, −0.18, and −0.27 year^−1^, respectively), indicating that the mean rate of forest gain slows down with increasing initial amounts. Compared with the cropland transition, the grassland and shrubland transitions are relatively less sensitive to changes in their initial amounts (mean absolute rates of change = 0.15 and 0.24, respectively). The initial amount at which the mean rate of forest gain is minimized ranges from 31% for bare to 47% for croplands. The models for the grassland and water transitions have the lowest value of explained deviance (0.24 and 0.40, respectively), showing a weaker model fit for these transitions (Fig. 7B; Supplementary Table 7 of Supplementary Appendix III).

#### Effect of the initial spatial arrangement of the transition LC

Here again, we find that LCI > 0.6 occurs predominantly when the transition LC occupies more than 80% of the grid cell (Supplementary Fig. 2 of Supplementary Appendix III). This strong correlation between the initial amount and spatial arrangement of the transition LC necessitates our focus on LCI values ≤ 0.6.

We generally find that the mean amount and rate of forest gain decreases with increasing fragmentation of the transition LCs (average rate of change = −0.13 and −0.25 year^−1^, respectively). We, however, find LC-specific differences in model results. The bare, shrubland, and wetland transitions show the highest sensitivity of mean amount of forest gain to changes in their initial LCI (mean absolute rates of change = 0.14, 0.24, and 0.14, respectively), while the cropland and grassland transitions are less sensitive (mean absolute rates of change = 0.08 and 0.09, respectively). The water transition has the lowest value of explained deviance (0.44), indicating a weaker relationship between its LCI and the mean amount of forest gain transitions (Fig. 7C; Supplementary Table 8 of Supplementary Appendix III). The relationship between initial spatial arrangement and mean forest gain changes substantially at coarser scales of analysis, especially for the cropland, grassland and shrubland transitions. For these transitions we see, that while at fine scales, mean forest gain typically decreases with increasing fragmentation, it assumes a hump-shaped pattern at coarser scales, indicating that mean forest gain initially increases with increasing fragmentation, but starts decreasing after reaching a peak amount (Fig. 7 and Supplementary Table 7 of SupplementarySupplementary Appendix IV). For effects on the mean rate of forest gain, we notice that the wetland and bare transitions are the most sensitive to changes in their starting LCI (mean absolute rates of change = 0.72 and 0.70, respectively), while the cropland and shrubland transitions are less sensitive (mean absolute rates of change = 0.55 and 0.43, respectively). The grassland transition has the lowest amount of explained deviance (0.47), indicating a weaker effect on the mean rate of forest gain transitions (Fig. 7D; Supplementary Table 9 of Supplementary Appendix III). The average proportion of null deviance explained across all LC transitions models testing the effects of the initial LCI on the mean amount and rate of forest gain are 0.74 and 0.68 respectively. The trends for the effects of the initial spatial arrangement on mean rate of forest gain tend to remain consistent at coarser scales of analysis.

## Discussion and Conclusion

Previous studies of global forest change typically focus on average estimates of change (FAO 2020; Hansen et al. 2013), and tend to disregard the diversity of pathways that forest landscapes may take between similar starting and ending forest extents. Uncovering these trajectories, may help elucidate the role of underlying socio-economic drivers contributing to global forest loss and recovery. Additionally, regional studies have also shown how LC transitions create distinct patterns of spatial disturbances in forest landscapes, suggesting that the initial amount and spatial arrangement of the non-forest, transition LC may indicate different stages in the process of forest loss and recovery (Alencar et al. 2023; Austin et al. 2017; Arima et al. 2016). Here, we investigated the effects of different LC transitions, along with the initial amount and spatial arrangement of the transition LC, on the amount and rate of global forest change.

We find that cropland and shrubland transitions dominate forest loss, reflecting the critical role of agricultural expansion and land-degradation in driving global deforestation (Feng et al. 2022; Pendrill et al. 2022; Hosonuma et al. 2012). Shrubland transitions, however, have a higher rate of mean forest loss as compared to the cropland transition, consistent with previous observations that forest transitions to shrublands require minimal human intervention and emerge post natural disturbances and land-abandonment (Pendrill et al. 2022; Johnstone et al. 2016; Cramer et al. 2008). However, rapid forest to cropland transitions driven by commercial and export-driven agricultural expansion challenges the notion that cropland transitions are always slower (Davis et al. 2020; Austin et al. 2017). At the same time, rapid rates of forest transition to bare and wetlands, and vice-versa are mostly concentrated in the northern reaches of Asia, Europe and America, suggesting the influence of climate change in accelerating these patterns of forest loss and recovery (IPCC 2021; Post et al. 2009). The frequent occurrence of the shrubland to forest transition across the African Sahel is supported by observations of continual greening due to climate change and improved land-management practices (Brandt et al. 2015; Dardel et al. 2014). On the other hand, faster rates of forest recovery due to the grassland transition, as compared to the cropland transition, is consistent with the claim that cropland areas tend to experience higher soil degradation, have reduced seed or sapling availability in the soil seed bank, and represent a highly disturbed ecosystem where the transition to forests requires the succession process to start from scratch (Chazdon 2008; Cramer et al. 2008; Holl 1999).

Additionally, the initial amount and spatial arrangement of the transition LC also has a strong and varying effect on the dynamics of forest loss. Firstly, we find that the amount of forest loss due to the cropland and settlement transitions demonstrate a heightened sensitivity to changes in their initial amounts and spatial arrangements. These results align with previous observations that claim anthropogenic transitions exhibit threshold effects - where forest availability becomes the predominant factor limiting forest exploitation (Meyfroidt and Lambin 2011; Defries et al. 2010). More generally, we find that fragmentation of the transition LC (implying increased interspersion of the forest and the transition LC), reduces the rate of forest loss, suggesting that dispersed forest patches may either be more resilient, or less accessible to drivers of loss, or perhaps occur in areas lacking economic incentives for large-scale and rapid deforestation (Tyukavina et al. 2018; Curtis et al. 2018). These observed fragmentation effects, however, exhibit scale-dependency, with the dampening effect on the rate of forest loss observed at finer resolutions reversing at coarser scales, indicating the contingency of forest arrangement and estimates of rate of change on the scale of analysis.

On the other hand, we also find that forest gain is most responsive to changes in the initial amounts of bare lands and wetlands. This is likely due to the fact that these LCs exhibit a strong threshold effect, where their over-abundance in the landscape can substantially limit the potential for forest regrowth, while their presence in limited quantities can assist in creating favorable conditions (such as enhanced nutrient availability, moisture retention, and reduced competition from other species) for forest regeneration (Prach and Walker 2011; Zedler and Kercher 2005). However, at coarserspatial resolutions, we observe that mean amount of forest gain is maximized at intermediate levels of fragmentation, indicating that forest recovery potential might actually benefit from a moderate amount of interspersion between forest patches and the transitioning LC. Furthermore, we generally find that increased fragmentation reduces the amount and rate of forest gain. This observation is in line with previous studies that state that increased fragmentation contributes to the increased isolation of forest patches, limiting the dispersal of seeds and the movement of species, thereby negatively affecting the process of forest regrowth (Haddad et al. 2015). These findings suggest that policies aimed at reducing agricultural expansion and promoting reforestation in wetland and shrubland areas could be particularly effective in mitigating global forest loss and enhancing forest recovery. Additionally, managing fragmentation levels in landscapes where forests are surrounded by bare and settlement areas could help control the rate of forest loss.

While our findings highlight the varying influence of LC transitions, along with their initial amounts and spatial arrangements, on global forest change, they are subject to the following limitations. First, our analysis was restricted to forest landscapes with more than 16 forest pixels undergoing LC change between 1992 and 2020 to enhance the signal-to-noise ratio and avoid spurious correlations that could obscure ecologically significant patterns. Second, we focused exclusively on trajectories of persistent forest loss/gain to ensure consistency in interpreting the amount and rate of forest change. However, examining omitted trajectories - such as those driven by temporally dependent factors like seasonal pests, wildfires, or rotational agriculture - could further improve our understanding of the drivers and patterns of global forest change. Third, while the dominant transition concept allowed us to focus on the most prevalent LC changes shaping global forest change, it inevitably overlooked less frequent but potentially impactful transitions. Despite this limitation, our method captures a substantial portion of global forest change dynamics, as evidenced by the close correspondence of our estimates with those from previous studies. Overall, prior to filtering any grids as detailed in Step 2.3.7. of the ‘Materials and Methods’ section, our method estimates a net forest loss of 0.50 million km² between 1992–2020. For comparison, previous studies using the CCI-LC dataset have reported a net forest decline of 0.36 and 0.41 million km² between 1992–2015 and 1992–2018, respectively (Radwan et al. 2021; Mousivand and Arsanjani 2019). The close alignment between these estimates validates the robustness of our method in accurately capturing global forest change, notwithstanding the exclusion of some less frequently occurring LC transitions. Furthermore, our assessment of classification uncertainty confirms that our estimates of the amount and rate of forest change are robust. However, higher uncertainties for the wetland and settlement transitions warrant more cautious interpretation of these results, while also highlighting the need for adequate validation data for all LC classes.

This study provides a foundational understanding of how LC transitions, along with their initial amount and spatial arrangement, influence the spatio-temporal trajectories of forest change. This may prove useful in establishing robust linkages between remotely-sensed LC changes and the socio-economic drivers propelling global forest change. Future research could expand this work in several directions. Investigating the effects of LC transitions on forest fragmentation would be valuable in assessing the sustainability of forest-based ecosystem services. Additionally, combining high-resolution spatial and temporal data with auxiliary information on the socio-economic and biophysical drivers could enhance our understanding of the causes and constraints shaping global forest loss and recovery. Such advancements would not only refine our knowledge of forest change dynamics but also inform more effective forest conservation and management strategies.

## Supplementary information

Supplementary information_Appendix

## Data Availability

The CCI-LC data used in this study are available at the following URL: (https://cds.climate.copernicus.eu/datasets/satellite-land-cover?tab=download). All scripts used to perform the data processing and analysis will be made available upon request.

## References

[CR1] Achard F, Beuchle R, Mayaux P, Stibig HJ, Bodart C, Brink A, Carboni S, Desclée B, Donnay F, Eva HD, Lupi A, Raši R, Seliger R, Simonetti D (2014) Determination of tropical deforestation rates and related carbon losses from 1990 to 2010. Glob Change Biol 20(8):2540–2554. 10.1111/gcb.12605 10.1111/gcb.12605 PMC4312855 24753029

[CR2] Alencar L, Escada MIS, Camargo JLC (2023) Forest regeneration pathways in contrasting deforestation patterns of Amazonia. Front Environ Sci 11:1–12. 10.3389/fenvs.2023.991695

[CR3] Arima EY, Walker RT, Perz S, Souza C (2016) Explaining the fragmentation in the Brazilian Amazonian forest. J Land Use Sci 11(3):257–277. 10.1080/1747423X.2015.1027797

[CR4] Austin KG, González-Roglich M, Schaffer-Smith D, Schwantes AM, Swenson JJ (2017) Trends in size of tropical deforestation events signal increasing dominance of industrial-scale drivers. Environm Res Lett 12(5) 10.1088/1748-9326/aa6a88

[CR5] Bradshaw CJA, Sodhi NS, Peh KSH, Brook BW (2007) Global evidence that deforestation amplifies flood risk and severity in the developing world. Glob Change Biol 13(11):2379–2395. 10.1111/j.1365-2486.2007.01446.x

[CR6] Brando PM, Paolucci L, Ummenhofer CC, Ordway EM, Hartmann H, Cattau ME, Rattis L, Medjibe V, Coe MT, Balch J (2019) Droughts, wildfires, and forest carbon cycling: a pantropical synthesis. Annu Rev Earth Planet Sci 47:555–581. 10.1146/annurev-earth-082517-010235

[CR7] Brandt M, Mbow C, Diouf AA, Verger A, Samimi C, Fensholt R (2015) Ground-and satellite-based evidence of the biophysical mechanisms behind the greening Sahel. Glob Change Biol 21(4):1610–1620. 10.1111/gcb.12807 10.1111/gcb.12807 25400243

[CR8] Ceccherini G, Duveiller G, Grassi G, Lemoine G, Avitabile V, Pilli R, Cescatti A (2020) Abrupt increase in harvested forest area over Europe after 2015. Nature 583(7814):72–77. 10.1038/s41586-020-2438-y 32612223 10.1038/s41586-020-2438-y

[CR9] Chazdon RL (2008) Beyond deforestation: restoring forests and ecosystem services on degraded lands. Science 320(5882):1458–1460 18556551 10.1126/science.1155365

[CR10] Cliff N (1993) Dominance statistics: ordinal analyses to answer ordinal questions. Psychological Bull 114(3):494–509. 10.1037/0033-2909.114.3.494

[CR11] Cochrane MA (2001) Synergistic interactions between habitat fragmentation and fire in evergreen tropical forests. Conserv Biol 15(6):1515–1521. 10.1046/j.1523-1739.2001.01091.x

[CR12] Cramer VA, Hobbs RJ, Standish RJ (2008) What’s new about old fields? Land abandonment and ecosystem assembly. Trends Ecol Evol 23(2):104–112. 10.1016/j.tree.2007.10.005 18191278 10.1016/j.tree.2007.10.005

[CR13] Curtis PG, Slay CM, Harris NL, Tyukavina A, Hansen MC (2018) Classifying drivers of global forest loss. Science 361(6407):1108–1111 30213911 10.1126/science.aau3445

[CR14] Dardel C, Kergoat L, Hiernaux P, Mougin E, Grippa M, Tucker CJ (2014) Re-greening Sahel: 30 years of remote sensing data and field observations (Mali, Niger). Remote Sens Environ 140:350–364. 10.1016/j.rse.2013.09.011

[CR15] Davis KF, Koo HI, Dell’Angelo J, D’Odorico P, Estes L, Kehoe LJ, Kharratzadeh M, Kuemmerle T, Machava D, Pais A, de JR, Ribeiro N, Rulli MC, Tatlhego M (2020) Tropical forest loss enhanced by large-scale land acquisitions. Nat Geosci 13(7):482–488. 10.1038/s41561-020-0592-3

[CR16] Defries RS, Rudel T, Uriarte M, Hansen M (2010) Deforestation driven by urban population growth and agricultural trade in the twenty-first century. Nat Geosci 3(3):178–181. 10.1038/ngeo756

[CR17] Di Gregorio, A. & Jansen, L. J. M. (2000) Land Cover Classification System (LCCS). http://www.fao.org/3/x0596e/X0596e00.htm#P-1_0

[CR18] Dietzel C, Oguz H, Hemphill JJ, Clarke KC, Gazulis N (2005) Diffusion and coalescence of the Houston Metropolitan Area: Evidence supporting a new urban theory. Environ Plan B Plan Des 32(2):231–246. 10.1068/b31148

[CR19] Ewers RM, Didham RK (2006) Confounding factors in the detection of species responses to habitat fragmentation. Biol Rev Camb Philos Soc 81(1):117–142. 10.1017/S1464793105006949 16318651 10.1017/S1464793105006949

[CR20] Fahrig L (2003) Effects of habitat fragmentation on biodiversity. Annu Rev Ecol Evol Syst 34:487–515. 10.1146/132419

[CR21] FAO (2020) Global forest resources assessment 2020: main report. Rome. 10.4060/ca9825en

[CR22] Feng Y, Zeng Z, Searchinger TD, Ziegler AD, Wu J, Wang D, He X, Elsen PR, Ciais P, Xu R, Guo Z, Peng L, Tao Y, Spracklen DV, Holden J, Liu X, Zheng Y, Xu P, Chen J, Zheng C (2022) Doubling of annual forest carbon loss over the tropics during the early twenty-first century. Nat Sustain 5(5):444–451. 10.1038/s41893-022-00854-3

[CR23] Defourny P, Lamarche C, Bontemps S, De Maet T, Van Bogaert E, Moreau I, Brockmann C, Boettcher M, Kirches G, Wevers J, Santoro M (2017) Land cover CCI product user guide version 2.0. http://maps.elie.ucl.ac.be/CCI/viewer/download/ESACCI-LC-Ph2-PUGv2_2.0.pdf

[CR24] Defourny P, Lamarche C, Flasse C, Brockmann C, Boettcher M, Kirches G (2021) Product user guide and specification ICDR land cover 2016-2020 https://community.esri.com/ccqpr47374/attachments/ccqpr47374/arcgis-pro-questions/23785/1/D3.3.12-v1.1_PUGS_ICDR_LC_v2.1.x_PRODUCTS_v1.1.1_APPROVED_Ver1.pdf

[CR25] Geist HJ, Lambin EF (2001) What drives tropical deforestation? A meta-analysis of proximate and underlying causes of deforestation based on subnational case study evidence? LUCC Report Series 4. LUCC International Project Office, Louvain-la-Neuve

[CR26] Haddad NM, Brudvig LA, Clobert J, Davies KF, Gonzalez A, Holt RD, Lovejoy TE, Sexton JO, Austin MP, Collins CD, Cook WM, Damschen EI, Ewers RM, Foster BL, Jenkins CN, King AJ, Laurance WF, Levey DJ, Margules CR, Townshend JR (2015) Habitat fragmentation and its lasting impact on Earth’s ecosystems. Sci Adv 1(2):1–10. 10.1126/sciadv.1500052 10.1126/sciadv.1500052 PMC4643828 26601154

[CR27] Hansen MC, Potapov PV, Moore R, Hancher M, Turubanova SA, Tyukavina A, Thau D, Stehman SV, Goetz SJ, Loveland TR, Kommareddy A, Egorov A, Chini L, Justice CO, Townshend JR (2013) High-resolution global maps of 21st-Century forest cover change. Science 850(November):850–854. 10.1126/science.1244693 10.1126/science.1244693 24233722

[CR28] Hastie TJ, Tibshirani RJ (1990) Generalized additive models. Chapman and Hall Boca Raton, FL

[CR29] Hesselbarth MHK, Sciaini M, With KA, Wiegand K, Nowosad J (2019) landscapemetrics: an open-source R tool to calculate landscape metrics. Ecography 42:1648–1657

[CR30] Hijmans R (2024) raster: Geographic data analysis and modeling. R package version 3.6-27. https://cran.r-project.org/web/packages/raster/raster.pdf

[CR31] Holl KD (1999) Factors limiting tropical rain forest regeneration in abandoned pasture: seed rain, seed germination, microclimate, and soil. Biotropica 31(2):229–242. 10.1111/j.1744-7429.1999.tb00135.x

[CR32] Hosonuma N, Herold M, De Sy V, De Fries RS, Brockhaus M, Verchot L, Angelsen A, Romijn E (2012) An assessment of deforestation and forest degradation drivers in developing countries. Environ Res Lett 7(4), 10.1088/1748-9326/7/4/044009

[CR33] IPBES (2019) Summary for policymakers of the global assessment report on biodiversity and ecosystem services of the Intergovernmental Science-Policy Platform on Biodiversity and Ecosystem Services. IPBES secretariat, Bonn, Germany, p 56

[CR34] IPCC (2021) Climate change 2021: the physical science basis. Contribution of Working Group I to the Sixth Assessment Report of the Intergovernmental Panel on Climate Change. Cambridge University Press, Cambridge, United Kingdom and New York, pp 2391, 10.1017/9781009157896

[CR35] Johnstone JF, Allen CD, Franklin JF, Frelich LE, Harvey BJ, Higuera PE, Mack MC, Meentemeyer RK, Metz MR, Perry GLW, Schoennagel T, Turner MG (2016) Changing disturbance regimes, ecological memory, and forest resilience. Front Ecol Environ 14(7):369–378. 10.1002/fee.1311

[CR36] Lambin EF, Meyfroidt P (2011) Global land use change, economic globalization, and the looming land scarcity. Proc Natl Acad Sci USA 108(9):3465–3472. 10.1073/pnas.1100480108 21321211 10.1073/pnas.1100480108 PMC3048112

[CR37] Lambin EF, Geist HJ (2006) Land-Use and Land-Cover Change: Local Processes and Global Impacts. Springer-Verlag, Berlin, Germany

[CR38] Laso Bayas JC, See L, Georgieva I, Schepaschenko D, Danylo O, Dürauer M, Bartl H, Hofhansl F, Zadorozhniuk R, Burianchuk M, Sirbu F, Magori B, Blyshchyk K, Blyshchyk V, Rabia AH, Pawe CK, Su YF, Ahmed M, Panging K, Fritz S (2022) Drivers of tropical forest loss between 2008 and 2019. Sci Data 9(1):1–9. 10.1038/s41597-022-01227-3 35365661 10.1038/s41597-022-01227-3 PMC8976004

[CR39] Lemoine-Rodríguez R, Inostroza L, Zepp H (2020) The global homogenization of urban form. An assessment of 194 cities across time. Landsc Urban Plan 204(April):103949. 10.1016/j.landurbplan.2020.103949

[CR40] Liu X, Yu L, Sia Y, Zhang C, Lu H, Yu C, Gong P (2018) Identifying patterns and hotspots of global land cover transitions using the ESA CCI land cover dataset. Remote Sens Lett 9(10):972–981. 10.1080/2150704X.2018.1500070

[CR41] Meyfroidt P, Lambin EF (2011) Global forest transition: Prospects for an end to deforestation. In Annual Review of Environment and Resources, 36, 10.1146/annurev-environ-090710-143732

[CR42] Mousivand A, Arsanjani JJ (2019) Insights on the historical and emerging global land cover changes: the case of ESA-CCI-LC datasets. Appl Geogr 106:82–92. 10.1016/j.apgeog.2019.03.010

[CR43] Munroe DK, van Berkel DB, Verburg PH, Olson JL (2013) Alternative trajectories of land abandonment: Causes, consequences and research challenges. Curr Opin Environ Sustain 5(5):471–476. 10.1016/j.cosust.2013.06.010

[CR44] Nowosad J, Stepinski TF (2019) Stochastic, empirically informed model of landscape dynamics and its application to deforestation scenarios. Geophys Res Lett 46(23):13845–13852. 10.1029/2019GL085952

[CR45] Nowosad J, Stepinski TF, Netzel P (2019) Global assessment and mapping of changes in mesoscale landscapes: 1992–2015. Int J Appl Earth Observ Geoinf 78(October 2018):332–340. 10.1016/j.jag.2018.09.013

[CR46] Ordway EM, Asner GP (2020) Carbon declines along tropical forest edges correspond to heterogeneous effects on canopy structure and function. Proc Natl Acad Sci, 117(14), 10.1073/pnas.1914420117/-/DCSupplemental 10.1073/pnas.1914420117 PMC7149387 32229568

[CR47] Pendrill F, Gardner TA, Meyfroidt P, Persson UM, Adams J, Azevedo T, Lima MGB, Baumann M, Curtis PG, De Sy V, Garrett R, Godar J, Goldman ED, Hansen MC, Heilmayr R, Herold M, Kuemmerle T, Lathuillière MJ, Ribeiro V, … West C (2022) Disentangling the numbers behind agriculture-driven tropical deforestation. Science, 377(6611) 10.1126/science.abm9267 10.1126/science.abm9267 36074840

[CR48] Post E, Forchhammer MC, Bret-Harte MS, Callaghan TV, Christensen TR, Elberling B, Fox AD, Gilg O, Hik DS, Høye TT, Ims RA, Jeppesen E, Klein DR, Madsen J, McGuire AD, Rysgaard S, Schindler DE, Stirling I, Tamstorf MP, Aastrup P (2009) Ecological dynamics across the arctic associated with recent climate change. Science 325(5946):1355–1358. 10.1126/science.1173113 19745143 10.1126/science.1173113

[CR49] Potapov P, Turubanova S, Hansen MC, Tyukavina A, Zalles V, Khan A, Song XP, Pickens A, Shen Q, Cortez J (2022) Global maps of cropland extent and change show accelerated cropland expansion in the twenty-first century. Nat Food 3(1):19–28. 10.1038/s43016-021-00429-z 37118483 10.1038/s43016-021-00429-z

[CR50] Prach K, Walker LR (2011) Four opportunities for studies of ecological succession. Trends Ecol Evol 26(3):119–123. 10.1016/j.tree.2010.12.007 21295370 10.1016/j.tree.2010.12.007

[CR51] Precinoto RS, Prieto PV, Figueiredo M, de SL, Lorini ML (2022) Edges as hotspots and drivers of forest cover change in a tropical landscape. Perspect Ecol Conserv 20(4):314–321. 10.1016/j.pecon.2022.07.001

[CR52] Radwan TM, Blackburn GA, Whyatt JD, Atkinson PM (2021) Global land cover trajectories and transitions. Sci Rep, 11(1) 10.1038/s41598-021-92256-2 10.1038/s41598-021-92256-2 PMC8211844 34140597

[CR53] Riitters K (2019) Pattern metrics for a transdisciplinary landscape ecology. Landsc Ecol 34(9):2057–2063. 10.1007/s10980-018-0755-4

[CR54] Rindfuss RR, Entwisle B, Walsh SJ, Mena CF, Erlien CM, Gray CL (2007) Frontier land use change: synthesis, challenges, and next steps. Ann Assoc Am Geographers 97(4):739–754. 10.1111/j.1467-8306.2007.00580.x

[CR55] Rudel TK, Coomes OT, Moran E, Achard F, Angelsen A, Xu J, Lambin E (2005) Forest transitions: towards a global understanding of land use change. Glob Environ Change 15(1):23–31. 10.1016/j.gloenvcha.2004.11.001

[CR56] Silva MPS, Camara G, Escada MIS, Modesto de Souza RC (2008) Remote-sensing image mining: Detecting agents of land-use change in tropical forest areas. Int J Remote Sens 29(16):4803–4822. 10.1080/01431160801950634

[CR57] Stevens N, Lehmann CER, Murphy BP, Durigan G (2017) Savanna woody encroachment is widespread across three continents. Glob Change Biol 23(1):235–244. 10.1111/gcb.13409 10.1111/gcb.13409 27371937

[CR58] Sturm J, Santos MJ, Schmid B, Damm A (2022) Satellite data reveal differential responses of Swiss forests to unprecedented 2018 drought. Glob Change Biol 28(9):2956–2978. 10.1111/gcb.16136 10.1111/gcb.16136 PMC9310759 35182091

[CR59] Turner MG, Gardner RH (2015) Landscape ecology in theory and practice: pattern and process, 2nd ed. Springer-Verlag, New York, NY

[CR60] Tyukavina A, Hansen MC, Potapov P, Parker D, Okpa C, Stehman SV, Kommareddy I, Turubanova S (2018) Congo Basin forest loss dominated by increasing smallholder clearing. Sci Adv 4(11):1–12. 10.1126/sciadv.aat2993 10.1126/sciadv.aat2993 PMC6221539 30417092

[CR61] van Vliet J, de Groot HLF, Rietveld P, Verburg PH (2015) Manifestations and underlying drivers of agricultural land use change in Europe. Landsc Urban Plan 133:24–36. 10.1016/j.landurbplan.2014.09.001

[CR62] Watson JEM, Evans T, Venter O, Williams B, Tulloch A, Stewart C, Thompson I, Ray JC, Murray K, Salazar A, McAlpine C, Potapov P, Walston J, Robinson JG, Painter M, Wilkie D, Filardi C, Laurance WF, Houghton RA, Lindenmayer D (2018) The exceptional value of intact forest ecosystems. Nat Ecol Evol 2(4):599–610. 10.1038/s41559-018-0490-x 29483681 10.1038/s41559-018-0490-x

[CR63] Wood SN (2011) Fast stable restricted maximum likelihood and marginal likelihood estimation of semiparametric generalized linear models. J R Stat Soc (B) 73(Issue 1):3–36

[CR64] Zedler JB, Kercher S (2005) Wetland resources: status, trends, ecosystem services, and restorability. Annu Rev Environ Resour 30:39–74. 10.1146/annurev.energy.30.050504.144248

