# Supplementary material for: The Effect of Dominant Land-Cover Transitions in Shaping Trajectories of Global Forest Change
Source: Environ Manage. 2025 Aug 5;75(10):2826–40. doi: 10.1007/s00267-025-02245-8 (PMC12457455; doi:10.1007/s00267-025-02245-8)
Supplement: Supplementary file 1 — Supplementary information_Appendix [file 267_2025_2245_MOESM1_ESM.docx]

**Appendix I**

**
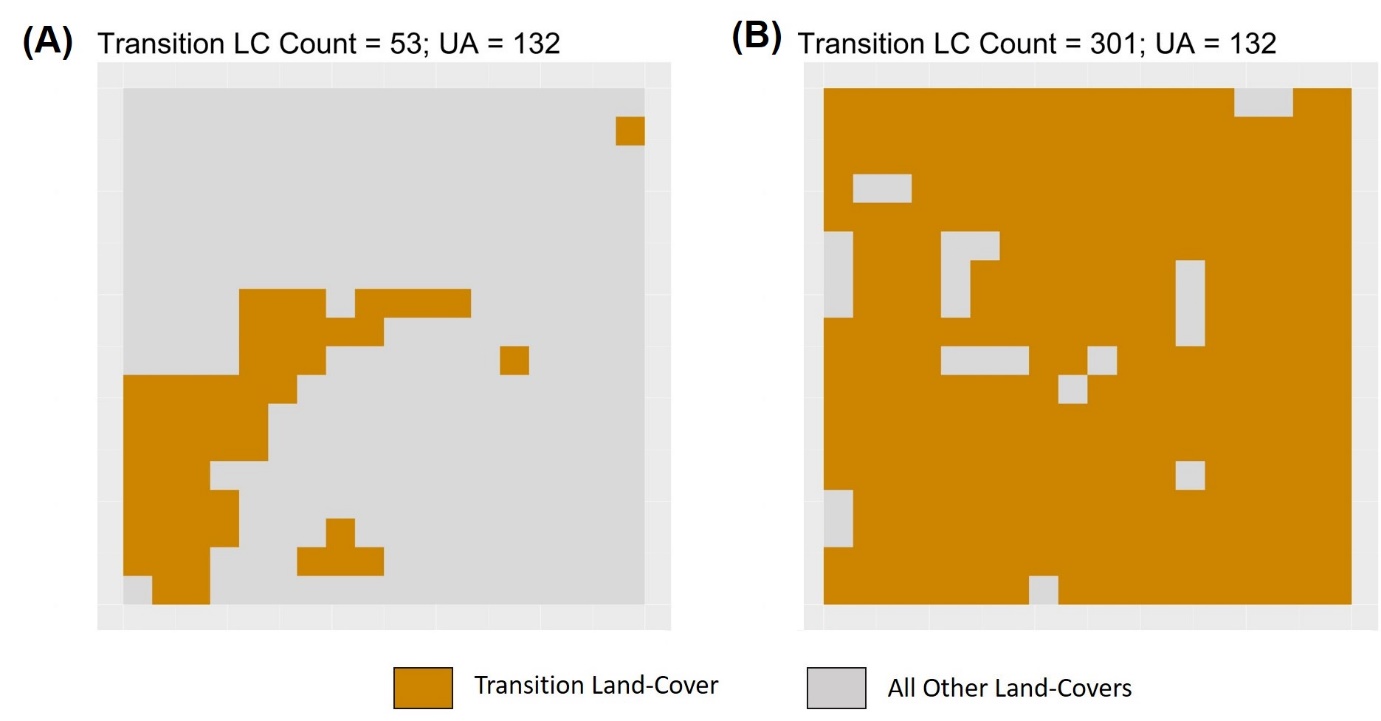
**

Figure 1: Landscapes showing the same value of unlike-adjacency (UA = 132) for low (53 pixels; plot A) and high (301 pixels; plot B) amounts of the transition LC. This figure highlights the problem of using the raw unlike-adjacency values as a measure of spatial arrangement, since similar values of UA can be got from vastly different spatial arrangements of the transition LC.


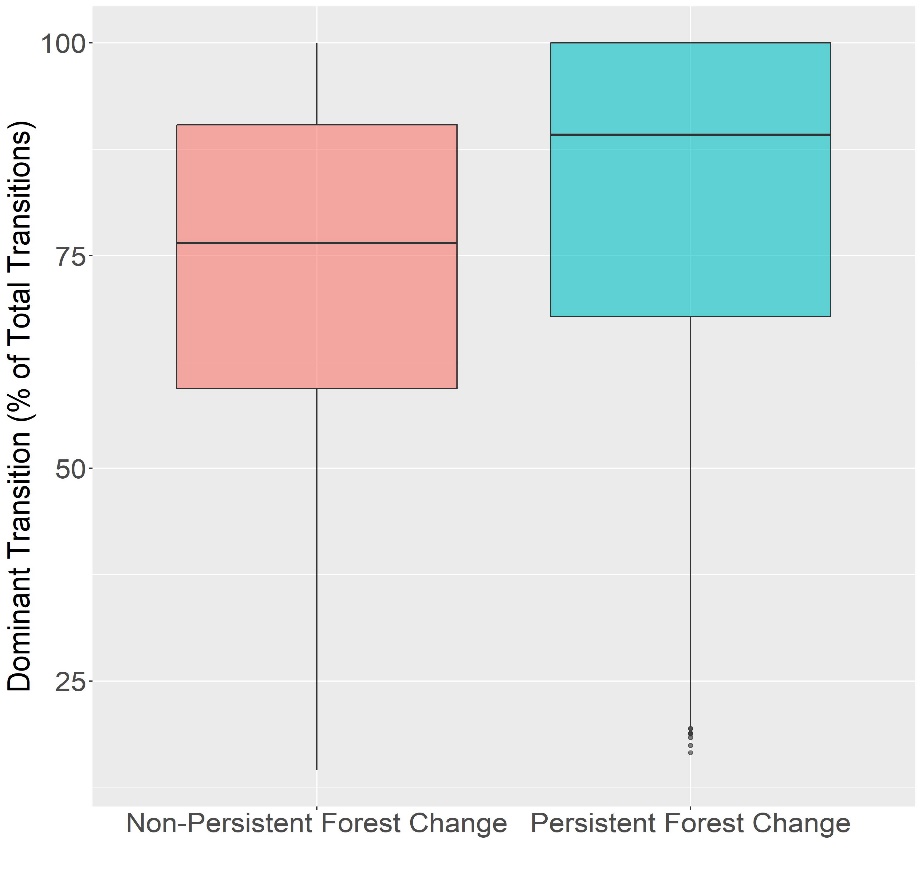


Figure 2: Boxplot showing the distribution of the prevalence of the dominant land-cover transitions, i.e. the percentage of the total LC transitions between 1992 and 2020 represented by the dominant LC transition, for grids with persistent and non-persistent forest change trajectories.


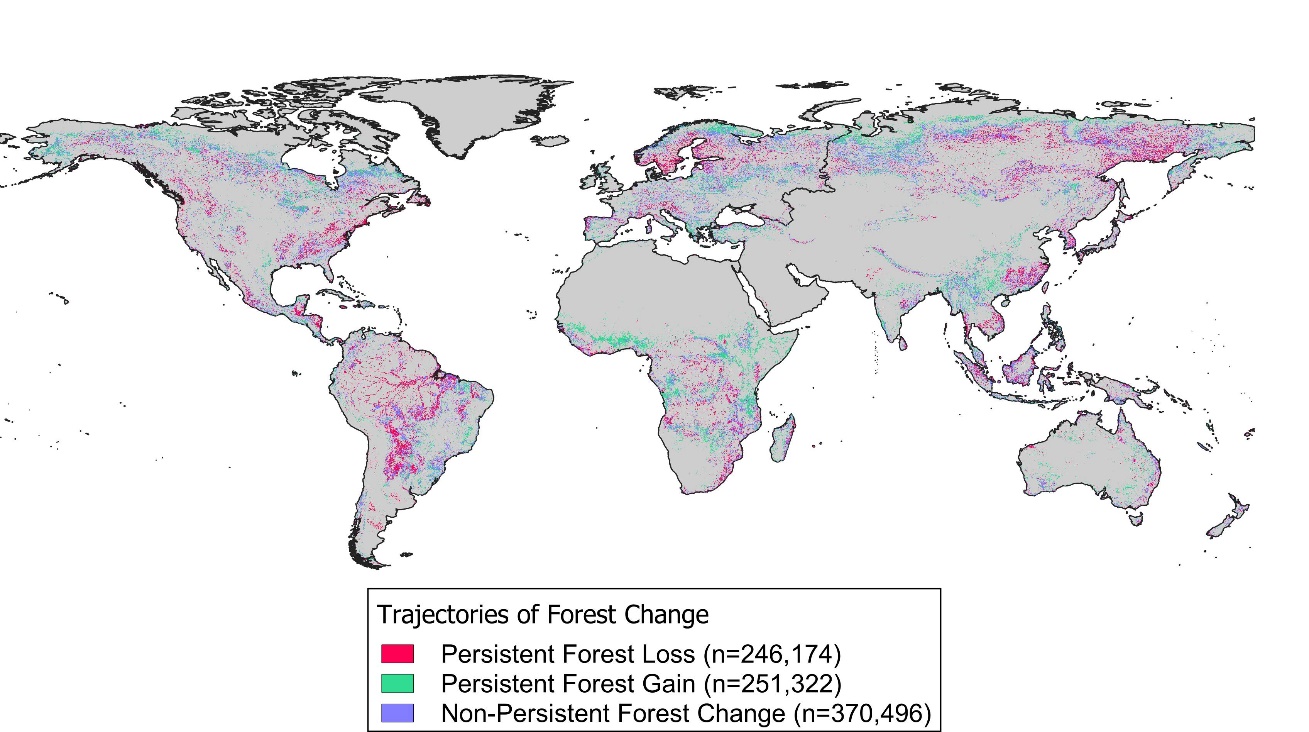


Figure 3: Global map of Forest Change Trajectories, where more than 16 forest pixels were gained or lost between 1992 and 2020. Grids with non-persistent forest change trajectories were not considered in our final analysis.

**
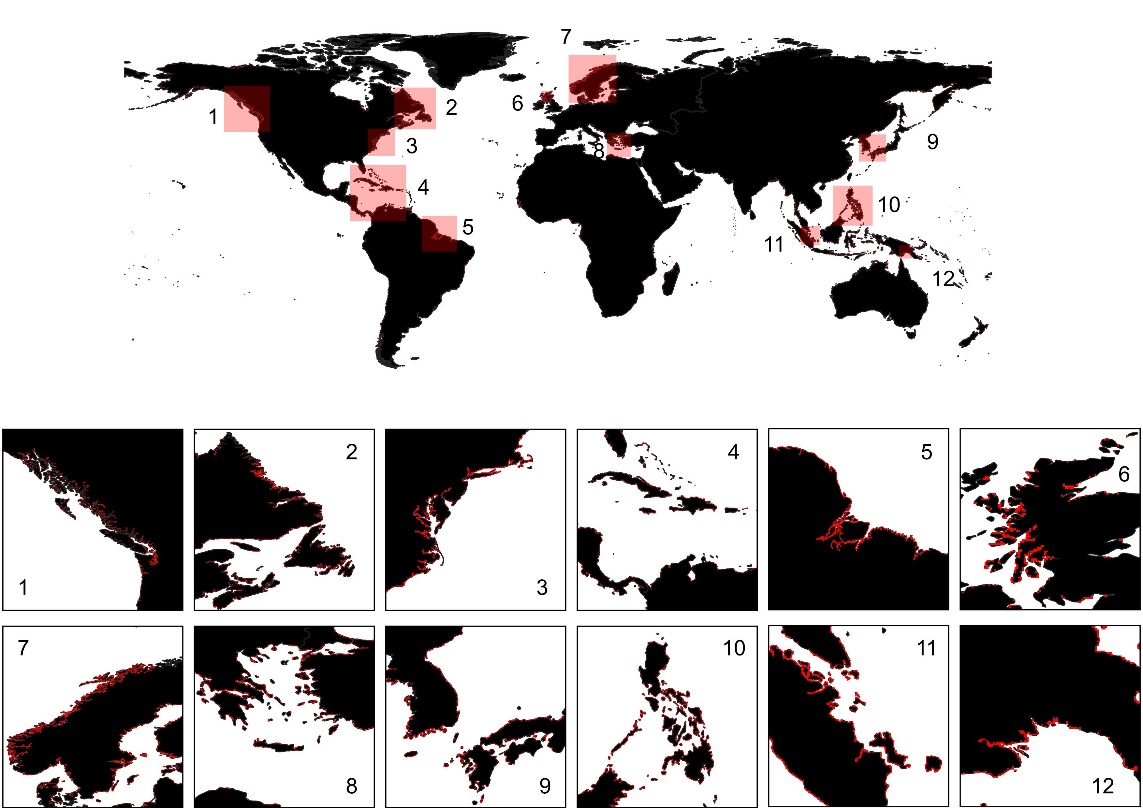
**

Figure 4: Grids (shown in ‘Red’) where the total number of pixels is not equal to 324. These grids were excluded from the analysis as they do not conform to the grid size that been used as a standard in evaluating the proportional amount and spatial arrangement of the transition LCs. Large-Scale Insets [1-12] showing coastlines near Vancouver Island, Canada (1), Newfoundland and Labrador provinces of Canada (2), New York, Delaware and Virginia of the United States of America (3), Caribbean Islands (4), the Amazon Delta (5), North-Western Scotland (6), the Scandinavian Peninsula (7), Greece and Turkey (8), Kyushu, Japan and South Korea (9), the Philippines (10), Southern Malaysia and Sumatra, Indonesia (11), and the South-East Coast of Papua New Guinea (12)

**
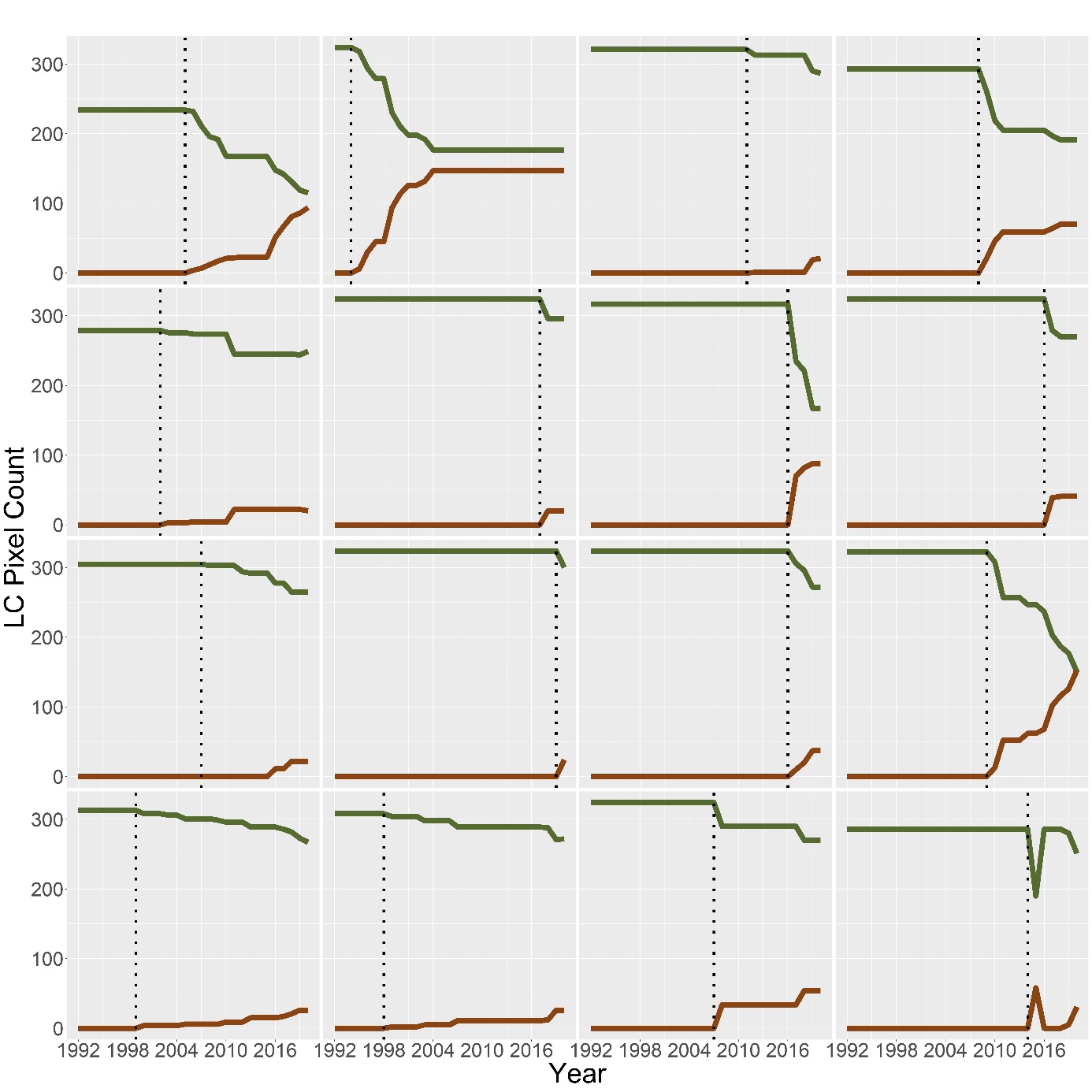
**

Figure 5: Time-Series for 16 grids showing the pixel-amount of forest (shown in Green) and the transition LC (shown in Red). For each of these grids, the value of the transition LC at the starting point of the interval of forest transition is zero. The starting point of the interval of transition is shown with a dotted vertical line. These grids (47,323 in all) were excluded from our analysis since it would be invalid to consider their starting amount and spatial arrangement.

**Appendix II**

**Calculating the Theoretical Maximum and Minimum UA:**

The following document details the steps taken in order to calculate the theoretical maximum and minimum Unlike-Adjacency (UA) for all possible values of pixel-count in a 18x18 landscape. Here, we assume that our landscape can be occupied by two classes only – a transition class ‘A’ that is expanding in a landscape previously occupied by LC ‘B’. The assumption of a binary landscape implies that the pixel-counts for the two classes are complementary and exhaustive, i.e., the sum of the number of pixels of the two classes will be 324 which is equal to the size of our grid.

Before we detail the steps taken to calculate the maximum and minimum UA, it is important to bear in mind that pixels lying in the 4 corners of our grid have 3 neighboring pixels, those lying along the edges of the grid and not in the corners have 5 neighboring pixels and those lying in the ‘core’ of the grid have 8 neighboring pixels.

**Calculating Maximum Unlike-Adjacency:**

In order to calculate maximum UA, we start with the configuration that is shown in Figure 1 below. Here LC ‘A’ is represented by ▲, and LC ‘B’ is represented by ‘blank’ cells. This starting configuration maximizes the UA between the two LCs, as each pixel of LC ‘A’ is surrounded by 8 pixels of LC ‘B’ (which is the maximum number of neighboring pixels possible), and would apply when we have less than or equal to 64 pixels of LC ‘A’ to distribute relative to LC ‘B’ for maximizing the UA between the two LCs. Therefore, given ***n*** pixels of LC ‘A’, where ***n*** ranges between 1 and 64, the overall maximum UA = 8 x ***n***.


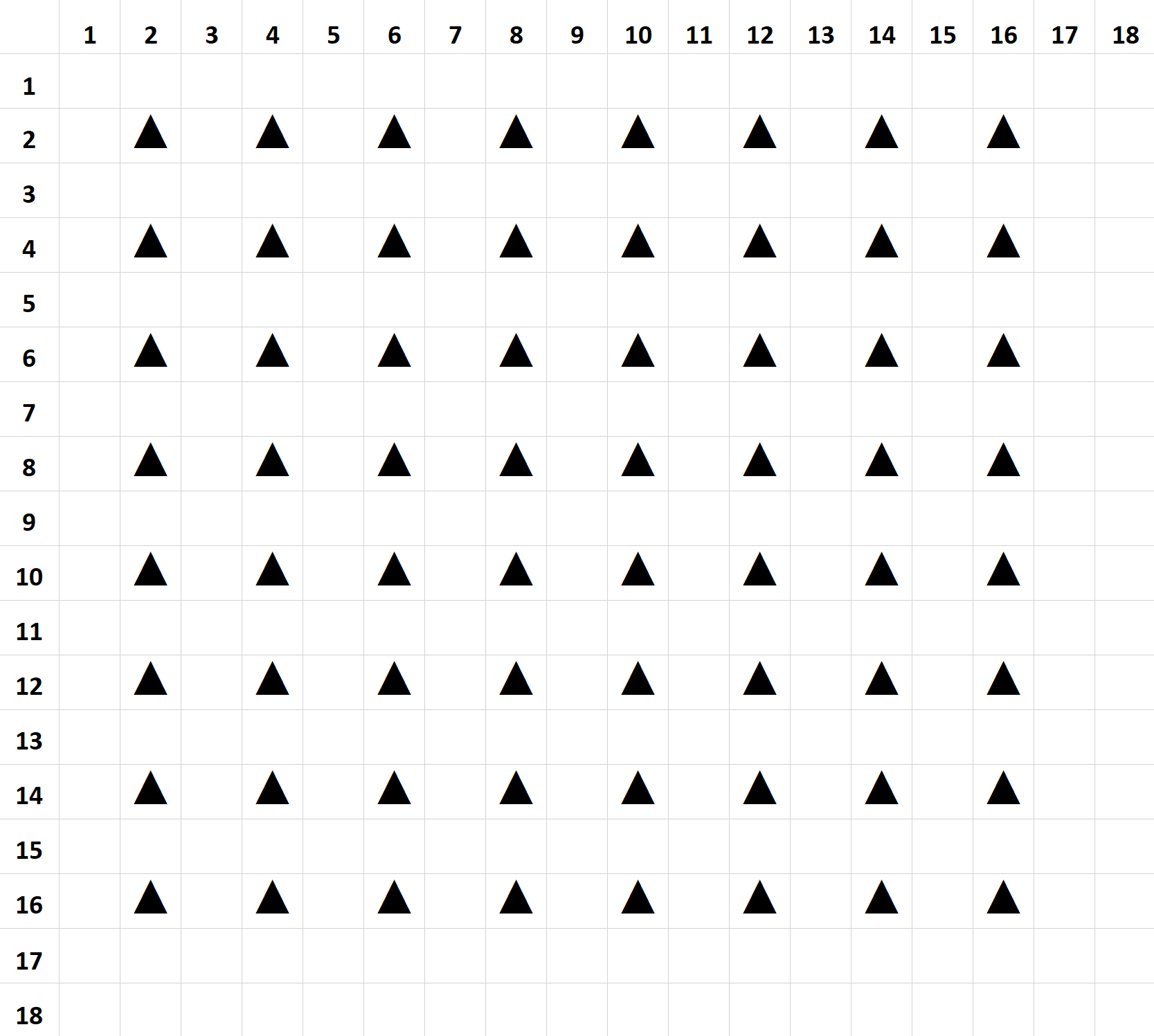


Figure 1: Starting Configuration to Maximize UA

Beyond this starting configuration, we observe that there are two differing sorting strategies for positioning pixels of class ‘A’ relative to class ‘B’ with the objective of maximizing UA. These sorting schemes produce slightly varying estimates of maximum UA for different values of the pixel-count of class ‘A’ and have been illustrated in Figures 2 and 3 below.

Each of the figures shown below are accompanied by a legend which indicates the different *levels* of grid-positions (indicated by symbols like ▲, ●, etc.) that should be *sequentially* occupied with the objective of maximizing the UA. For each legend-unit, we have also indicated the increment to overall UA that would be contributed by every additional pixel (*Adjacency/Pixel*), the number of pixels available at each level (*Number of Pixels*), the total UA increment possible if all available positions were to be occupied (*Total Adjacency*), and finally a column to indicate the cumulative sum of pixels up to that level (*Total Number of Pixels*).

To illustrate the utility of this sorting schema, consider the following two examples as they would apply to the sorting scheme represented in Figure 2 below.

**Example 1**: If we wanted to distribute 70 pixels of class ‘A’ in the landscape, then we would occupy the 64 positions indicated by ▲ (also shown in Figure 1 above) and thereafter, the 6 remaining pixels should be slotted in any 6 out of the 16 positions indicated by ●. With each added pixel of LC ‘A’, at the locations specified by ●, our overall UA is increased by a value of 5 (the *adjacency/pixel* value indicated in the legend corresponding to ●). Therefore, when distributing 70 pixels of the transition LC ‘A’, in a landscape previously occupied by LC ‘B’ and with the objective maximizing the UA between the two LCs, our overall UA = (64 x 8) + (6 x 5) = 542.

**Example 2:** If we wanted to distribute 160 pixels of class ‘A’ in the landscape, then we would occupy the 64 positions indicated by ▲, followed by the 16 positions indicated by ●, followed by the 64 positions indicated by ■, followed by the 9 positions indicated by ֎, and finally, the 7 remaining pixels should be slotted in any 7 out of the 9 positions indicated by ♣. Therefore, in this case, our overall UA = (64 x 8) + (16 x 5) + (64 x 4) + (9 x 3) + (7 x 1) = 882.

**
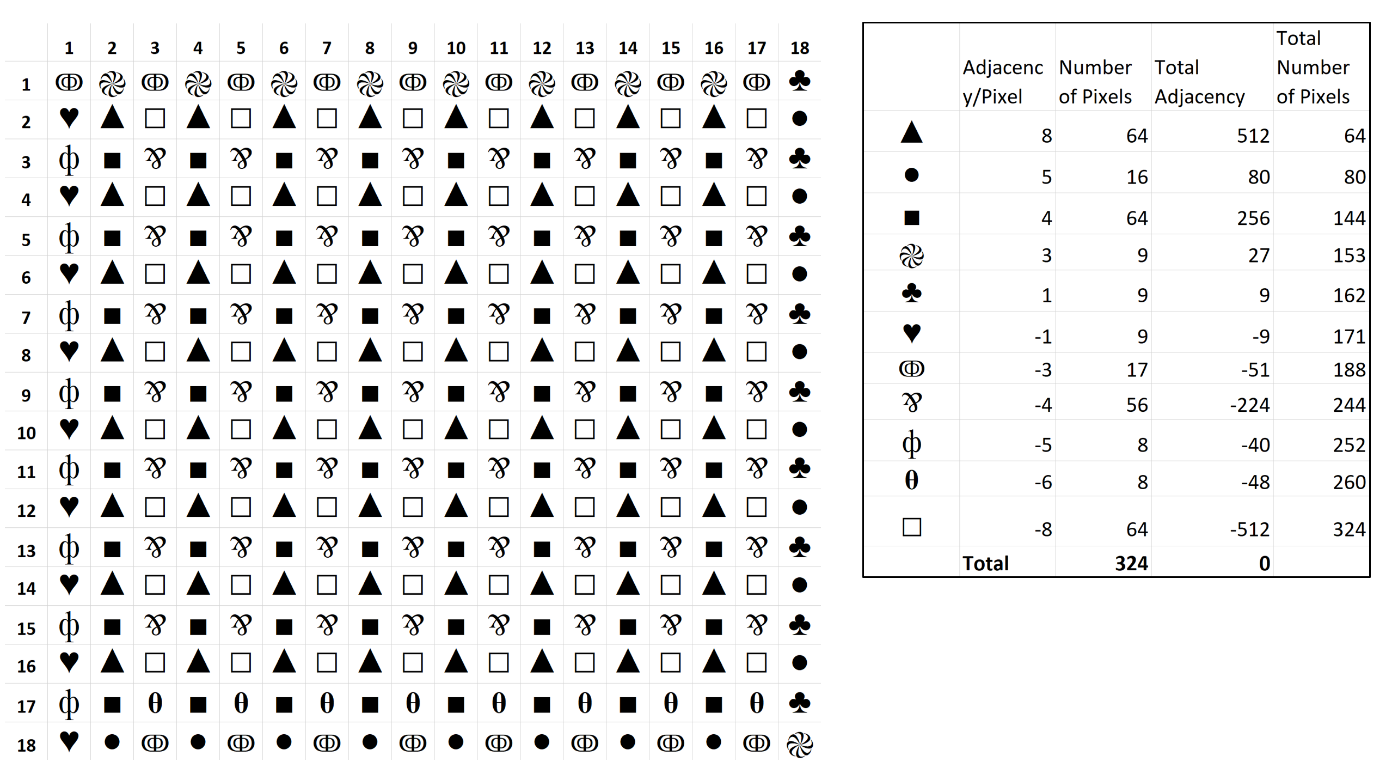
**

Figure 2: Schematic Representation for the Sorting Algorithm 1 for Maximizing Unlike-Adjacency

**
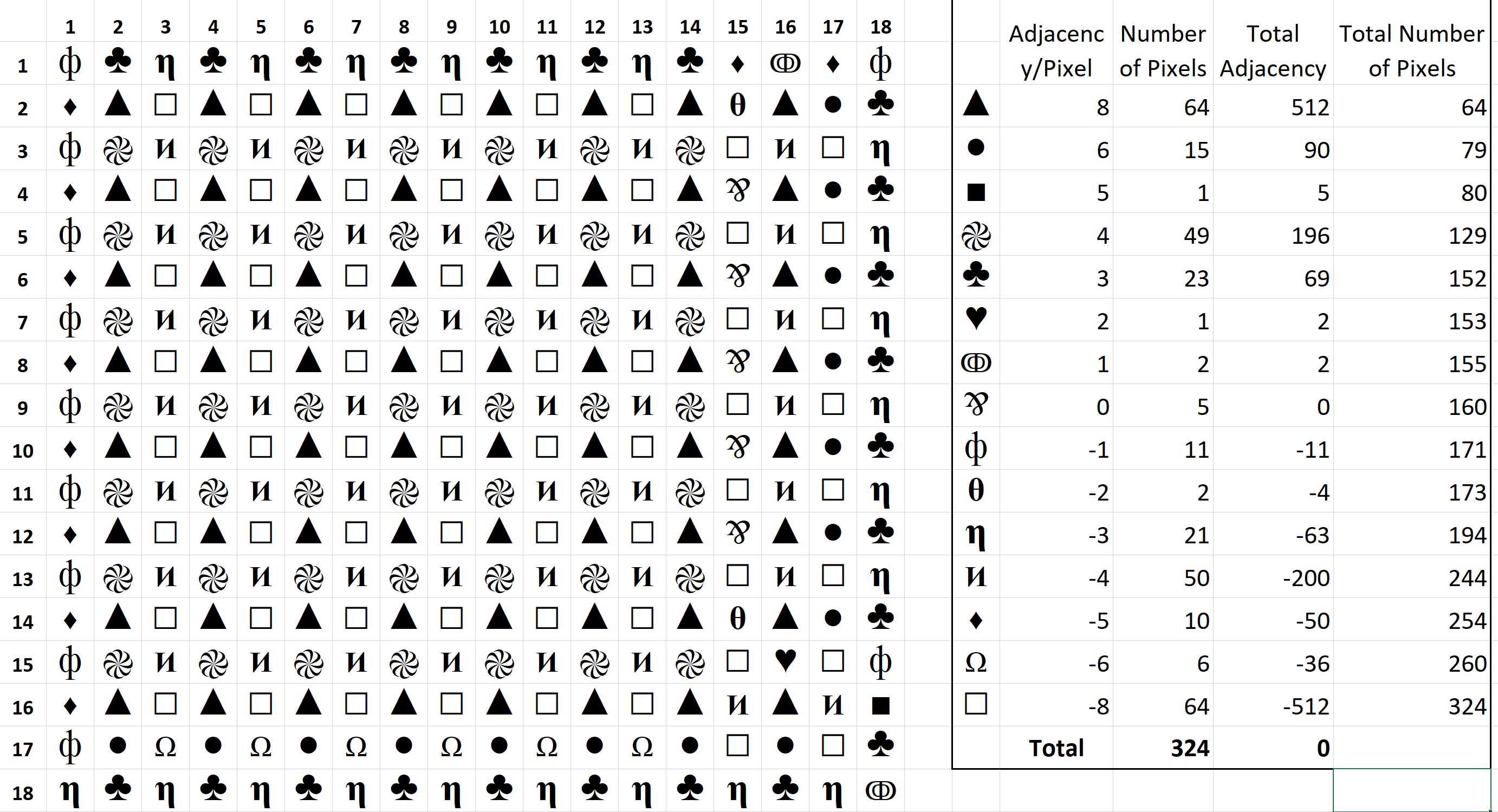
**

Figure 3: Schematic Representation for the Sorting Algorithm 2 for Maximizing Unlike-Adjacency

These two sorting schemas produce comparable but slightly differing estimates of UA. In Figure 4, we plotted the difference between these two estimates against all values of the pixel-count of class ‘A’. These differences arise due to boundary or edge effects and depend on whether the optimization of maximum UA is desired in the short run (when the amount of LC ‘A’ is small) or in the long-run (when the amount of LC ‘A’ is large). Here, we can see that sorting algorithm 2 produces larger estimates of UA when the pixel-count of class ‘A’ lies between 65 and 144 pixels, while sorting algorithm 1 produces larger estimates of UA when the pixel-count of class ‘A’ lies between 145 and 261 pixels. At all other points on the interval the two sorting schemes produce equivalent estimates of UA. We use the maximum of the two values at each pixel-count of class ‘A’ to estimate our theoretical maximum UA.


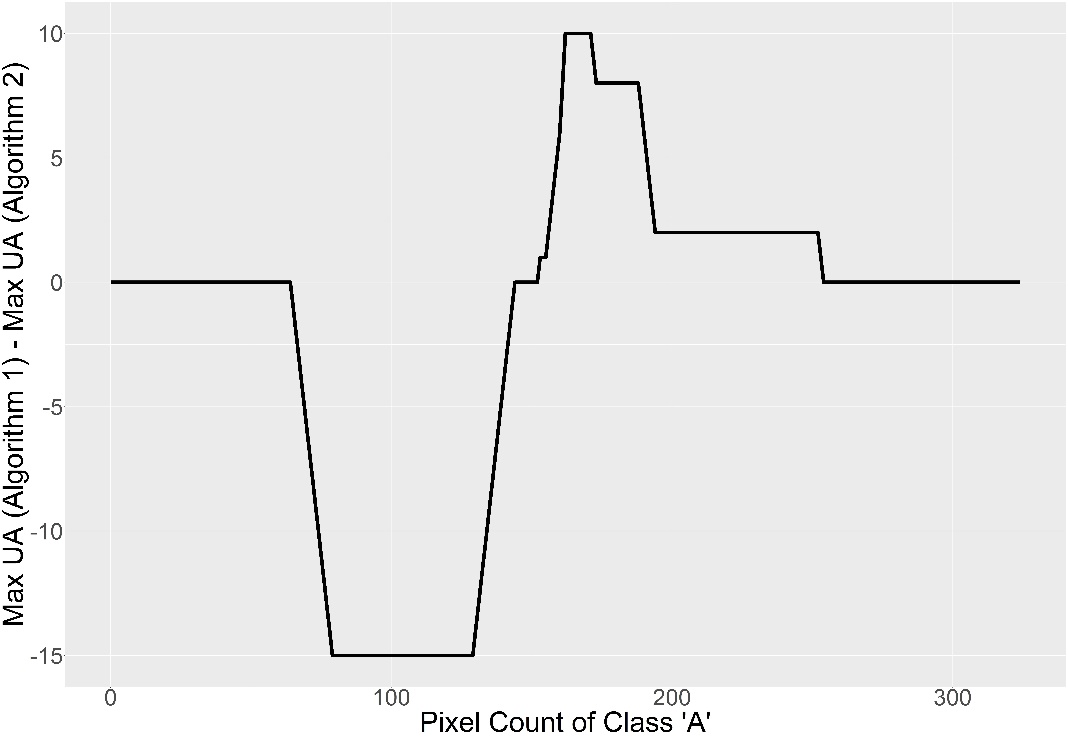


Figure 4: Plot of the difference in the maximum unlike-adjacency estimates produced by sorting algorithm 1 and 2 for every possible number of pixels of land-cover ‘A’ possible in the grid cell.

**Calculating Minimum Unlike-Adjacency:**

Here, we would like to start by introducing the spatial configuration shown in Figure 5 below. Here LC ‘A’ occupies positions demarcated by symbols (■, ●, ▲), while LC ‘B’ occupies ‘blank’ cells. We start with this configuration because we believe it is intuitively understood that such a spatial distribution minimizes the number of shared edges between the two LCs, and hence the overall UA. Here, we see that the edge pixels of LC ‘A’ represented by ■ and ● are lying adjacent to 2 and 3 pixels each of LC ‘B’ respectively. Further pixels of LC ‘A’ represented by ▲ share no edges with LC ‘B’. Therefore, the overall UA for such a configuration would be (2 x 2) + ((18-2) x 3) = 52. If an additional pixel of class ‘A’ were to be added to the configuration of Figure 5, and be placed at position Row=1, Column=6, the overall UA would increase to a value of 53. Thereafter, all additional pixels if sequentially placed in Column=6 and at Row positions 2-17 will maintain the overall UA at a constant value of 53. The final pixel of Column=6 when added at Row=18 would return us back to the configuration shown in Figure 5 above, albeit with one added column, and to an overall UA of 52.


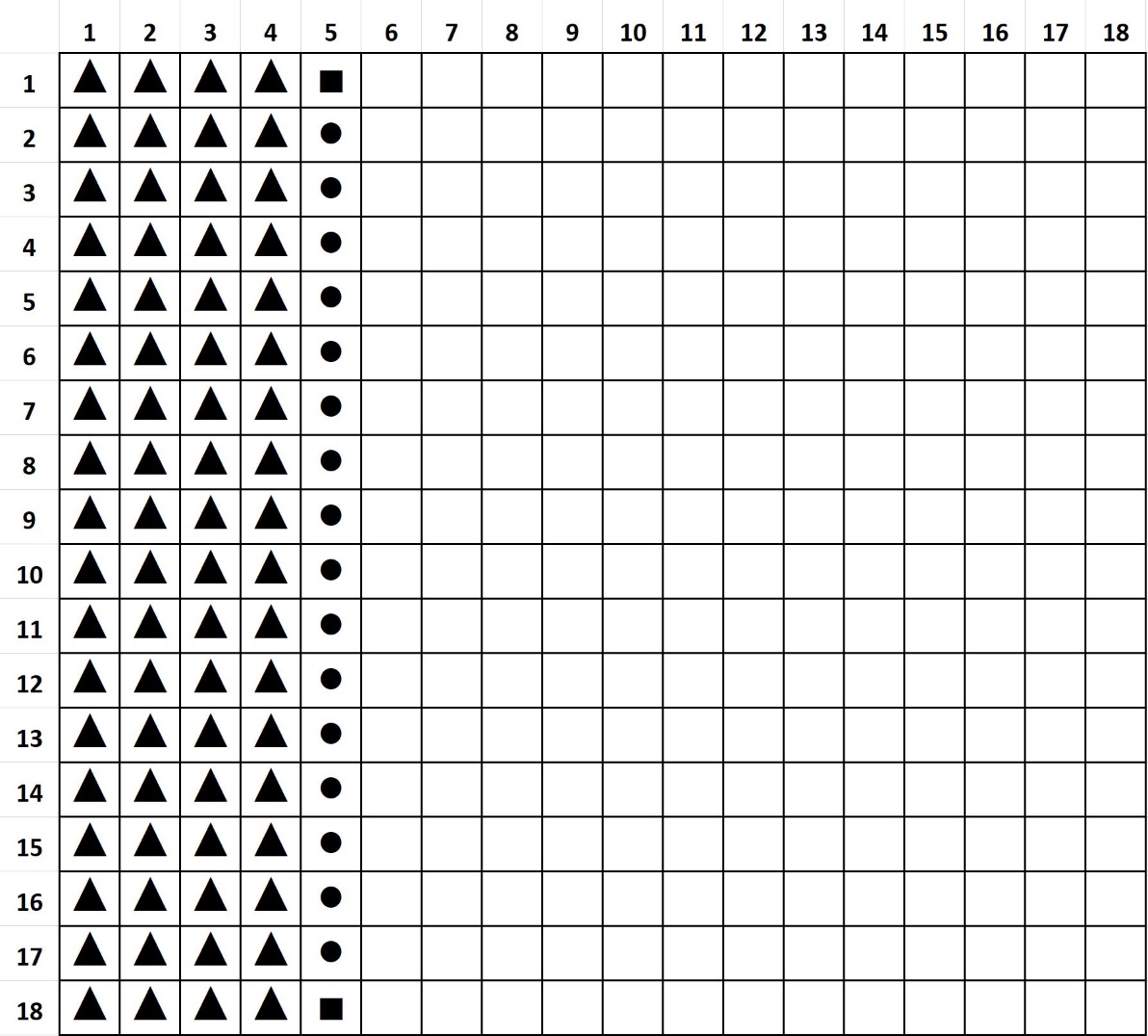


Figure 5: Configurational Representation of Theoretical Segmentation between two LCs that minimizes UA

Now, in order to even have the starting configuration as shown in Figure 5, we require a minimum of 18 pixels of LC ‘A’ to occupy an entire row or column in our 18 x 18 grid. Therefore, we now consider the optimum spatial configurations of the two LCs starting from extremely small values of LC ‘A’.

The spatial configuration in order to minimize the UA when we have 1 pixel of LC ‘A’ is shown in Figure 6 below. In this configuration the UA between the two classes would be equal to the minimum possible value of 3.

**
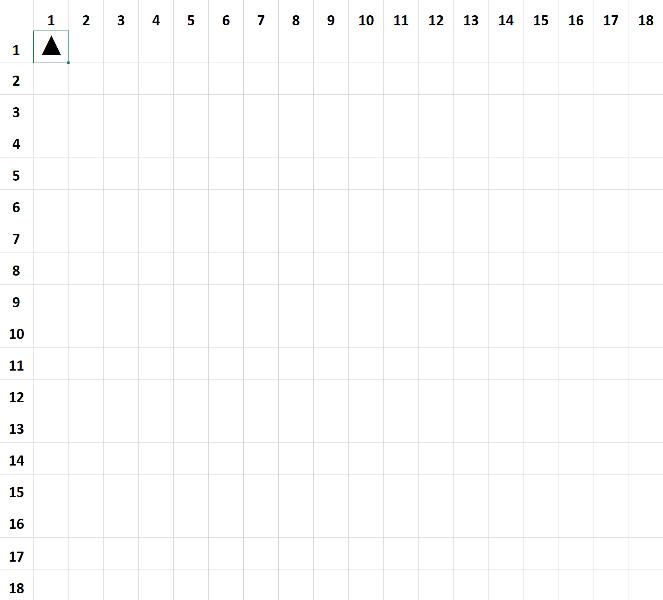
**

Figure 6: Starting Configuration to Minimize UA

Thereafter, as the number of pixels of class ‘A’ keeps increasing in the landscape, the configuration of class ‘A’ starts tracing a ‘step-wise’ pattern in the landscape, which accomplishes the objective of minimizing the overall UA. This ‘step-wise’ pattern is shown in Figure 7 below for when the count of class ‘A’ increases from 1 to 36 pixels.


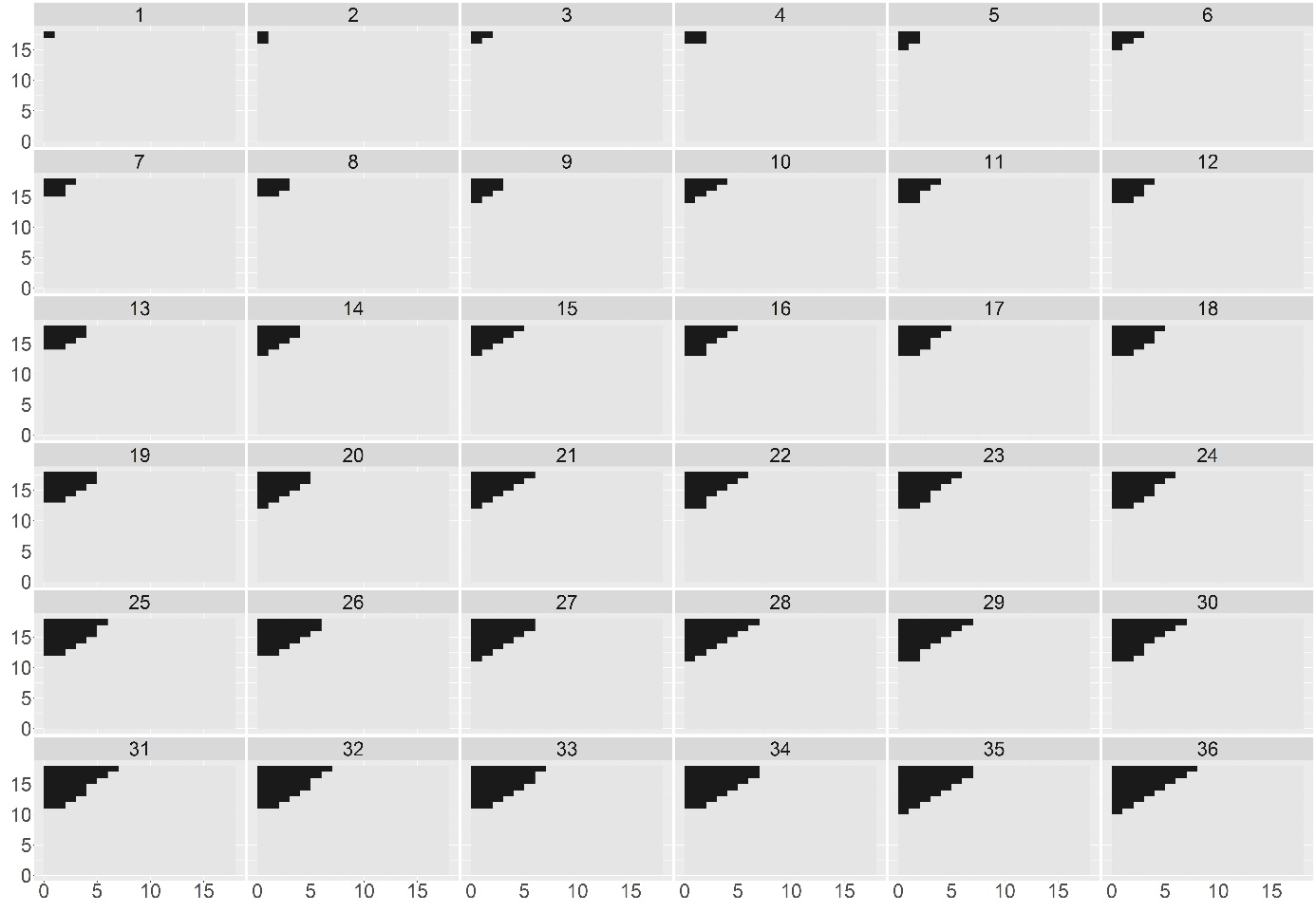


Figure 7: Configuration of Class ‘A’ to Minimize UA for Pixel-Count between 1 and 36 pixels

Now, in order to find the distribution pattern that would minimize the overall UA for all pixel-counts of LC ‘A’, we need to locate the threshold of pixel-count at which the UA generated from this ‘step-wise’ pattern exceeds the theoretical limit of UA = 52 that was explained in the context of Figure 5. To this end, we observe that a perfect ‘step-wise’ pattern can be represented as a sum of ***n*** consecutive natural numbers as shown in Figure 8 below.

| 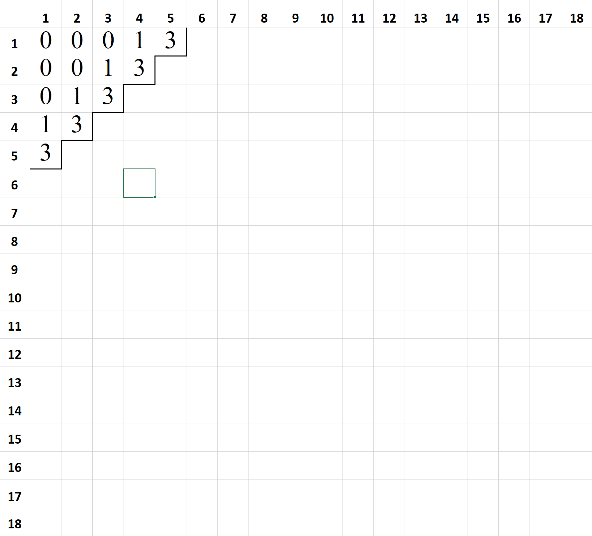 | 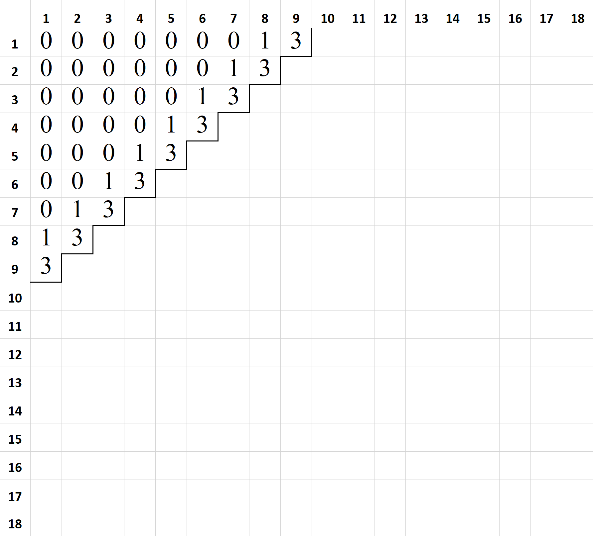 |
| --- | --- |

Figure 8: Representation of the UA for a 5-step and a 9-step pattern

In Figure 8, we see a perfect ‘step-wise’ pattern of 5 and 9 steps that accounts for the distribution of 15 and 45 pixels respectively. The UA of each pixel is annotated at the pixel positions shown in the figure, and provides illustrative proof that the overall UA of a perfect ‘step-wise’ pattern of ***n*** steps is equal to (3 x n) + (n - 1). Now, perfect ‘step-wise’ patterns are applicable when pixel counts form a sum of ***n*** consecutive natural numbers, i.e., when pixel-count is an element of the set of ***P*** = [1,3,6,10,15,21,28,36,45,55…]. For when the pixel-count is an intermediary value - lying in-between the numbers of set ***P*** - the overall UA can be estimated by using the following procedure:

1. Identify the pixel-count in set ***P*** that is closest to, but smaller than, the pixel-count for which the overall UA needs to be estimated.
2. Calculate the corresponding number of steps (**NSteps**), and the UA for the pixel-count (**Base UA**) identified in Step 1.
3. Calculate the difference between the pixel-count under consideration, and the pixel-count identified from set ***P***.
4. If the difference in Step 3 is less than or equal to (**NSteps** -1) then overall UA is equal to **Base UA** + 2, else overall UA is equal to **Base UA** + 3.

Using this procedure to calculate the overall UA for ‘step-wise’ distributions of pixels of LC ‘A’, we can identify the threshold value at which the overall UA would surpass the theoretical limit of UA=52. In the case of 18 x 18 grids, this threshold value of pixel-count = 91 pixels. This means that when pixel-count of LC ‘A’ lies between 1-91 pixels, LC ‘A’ should be distributed in a manner closely approximating a ‘step-wise’ pattern as illustrated in Figure 7. Furthermore, it is important to bear in mind that given limitations of a binary landscape the hypothetical distribution of pixels in the landscape (with the objective of minimizing the overall UA) would be symmetrical in nature. This implies that when pixel-count of LC ‘A’ lies between 91 and 233 (324 - 91) pixels, the pixels of LC ‘A’ should be distributed in a manner similar to what is described in association with Figure 5, with the overall UA fluctuating between values of 52 and 53. At all other values of pixel-counts, i.e., when pixel-count of LC ‘A’ lies between 1-91 or between 234-323 the spatial distribution of LC ‘A’ should follow a ‘step-wise’ pattern.

**Appendix III**

| **Table 1:** Cliff’s Test Statistics for the Amount and Rate of Forest Loss | | | | | | |
| --- | --- | --- | --- | --- | --- | --- |
| **Variable** | **Group1** | **Group2** | **Cliff_Delta** | **CI_lower** | **CI_upper** | **Magnitude*** |
| Amount | Bare | Settlement | 0.035 | 0.014 | 0.057 | Negligible |
| Amount | Bare | Cropland | -0.11 | -0.121 | -0.099 | Negligible |
| Amount | Bare | Grassland | 0.045 | 0.032 | 0.058 | Negligible |
| Amount | Bare | Shrubland | -0.008 | -0.019 | 0.004 | Negligible |
| Amount | Bare | Water | 0.125 | 0.11 | 0.139 | Negligible |
| Amount | Bare | Wetland | 0.16 | 0.147 | 0.173 | Small |
| Amount | Settlement | Cropland | -0.146 | -0.163 | -0.128 | Negligible |
| Amount | Settlement | Grassland | 0.012 | -0.008 | 0.031 | Negligible |
| Amount | Settlement | Shrubland | -0.042 | -0.061 | -0.023 | Negligible |
| Amount | Settlement | Water | 0.093 | 0.07 | 0.115 | Negligible |
| Amount | Settlement | Wetland | 0.13 | 0.11 | 0.151 | Negligible |
| Amount | Cropland | Grassland | 0.152 | 0.145 | 0.159 | Small |
| Amount | Cropland | Shrubland | 0.101 | 0.095 | 0.106 | Negligible |
| Amount | Cropland | Water | 0.232 | 0.224 | 0.241 | Small |
| Amount | Cropland | Wetland | 0.259 | 0.251 | 0.266 | Small |
| Amount | Grassland | Shrubland | -0.052 | -0.06 | -0.044 | Negligible |
| Amount | Grassland | Water | 0.076 | 0.065 | 0.087 | Negligible |
| Amount | Grassland | Wetland | 0.117 | 0.107 | 0.126 | Negligible |
| Amount | Shrubland | Water | 0.13 | 0.121 | 0.139 | Negligible |
| Amount | Shrubland | Wetland | 0.166 | 0.158 | 0.174 | Small |
| Amount | Water | Wetland | 0.053 | 0.042 | 0.064 | Negligible |
| Rate | Bare | Settlement | 0.785 | 0.773 | 0.796 | Large |
| Rate | Bare | Cropland | 0.078 | 0.066 | 0.09 | Negligible |
| Rate | Bare | Grassland | 0.022 | 0.009 | 0.035 | Negligible |
| Rate | Bare | Shrubland | 0.044 | 0.031 | 0.056 | Negligible |
| Rate | Bare | Water | 0.193 | 0.179 | 0.208 | Small |
| Rate | Bare | Wetland | -0.001 | -0.014 | 0.012 | Negligible |
| Rate | Settlement | Cropland | -0.775 | -0.785 | -0.765 | Large |
| Rate | Settlement | Grassland | -0.801 | -0.811 | -0.79 | Large |
| Rate | Settlement | Shrubland | -0.805 | -0.815 | -0.794 | Large |
| Rate | Settlement | Water | -0.738 | -0.75 | -0.725 | Large |
| Rate | Settlement | Wetland | -0.794 | -0.804 | -0.784 | Large |
| Rate | Cropland | Grassland | -0.054 | -0.062 | -0.046 | Negligible |
| Rate | Cropland | Shrubland | -0.032 | -0.038 | -0.026 | Negligible |
| Rate | Cropland | Water | 0.12 | 0.11 | 0.129 | Negligible |
| Rate | Cropland | Wetland | -0.076 | -0.084 | -0.068 | Negligible |
| Rate | Grassland | Shrubland | 0.021 | 0.013 | 0.029 | Negligible |
| Rate | Grassland | Water | 0.168 | 0.157 | 0.179 | Small |
| Rate | Grassland | Wetland | -0.023 | -0.033 | -0.014 | Negligible |
| Rate | Shrubland | Water | 0.143 | 0.134 | 0.153 | Negligible |
| Rate | Shrubland | Wetland | -0.045 | -0.054 | -0.037 | Negligible |
| Rate | Water | Wetland | -0.188 | -0.199 | -0.176 | Small |
| *Magnitude is “Negligible” when abs(Cliff_Delta) <0.147; “Small” when 0.147≤abs(Cliff Delta)<0.33; “Medium” when 0.33≤abs(Cliff Delta)<0.474; and “Large” when abs(Cliff Delta) ≥ 0.474. | | | | | | |

| **Table 2:** Cliff’s Test Statistics for the Amount and Rate of Forest Gain | | | | | | |
| --- | --- | --- | --- | --- | --- | --- |
| **Variable** | **Group1** | **Group2** | **Cliff_Delta** | **CI_lower** | **CI_upper** | **Magnitude*** |
| Amount | Bare | Cropland | -0.016 | -0.024 | -0.008 | Negligible |
| Amount | Bare | Grassland | 0.064 | 0.055 | 0.073 | Negligible |
| Amount | Bare | Shrubland | -0.024 | -0.032 | -0.016 | Negligible |
| Amount | Bare | Water | -0.008 | -0.03 | 0.014 | Negligible |
| Amount | Bare | Wetland | -0.089 | -0.098 | -0.08 | Negligible |
| Amount | Cropland | Grassland | 0.08 | 0.072 | 0.087 | Negligible |
| Amount | Cropland | Shrubland | -0.008 | -0.014 | -0.003 | Negligible |
| Amount | Cropland | Water | 0.008 | -0.013 | 0.029 | Negligible |
| Amount | Cropland | Wetland | -0.073 | -0.08 | -0.066 | Negligible |
| Amount | Grassland | Shrubland | -0.087 | -0.094 | -0.079 | Negligible |
| Amount | Grassland | Water | -0.071 | -0.093 | -0.049 | Negligible |
| Amount | Grassland | Wetland | -0.152 | -0.161 | -0.143 | Small |
| Amount | Shrubland | Water | 0.016 | -0.005 | 0.037 | Negligible |
| Amount | Shrubland | Wetland | -0.064 | -0.071 | -0.056 | Negligible |
| Amount | Water | Wetland | -0.08 | -0.102 | -0.059 | Negligible |
| Rate | Bare | Cropland | 0.036 | 0.029 | 0.044 | Negligible |
| Rate | Bare | Grassland | -0.134 | -0.144 | -0.125 | Negligible |
| Rate | Bare | Shrubland | -0.062 | -0.07 | -0.054 | Negligible |
| Rate | Bare | Water | 0.024 | 0.003 | 0.045 | Negligible |
| Rate | Bare | Wetland | 0.019 | 0.01 | 0.028 | Negligible |
| Rate | Cropland | Grassland | -0.167 | -0.174 | -0.16 | Small |
| Rate | Cropland | Shrubland | -0.096 | -0.102 | -0.091 | Negligible |
| Rate | Cropland | Water | -0.01 | -0.029 | 0.01 | Negligible |
| Rate | Cropland | Wetland | -0.016 | -0.023 | -0.009 | Negligible |
| Rate | Grassland | Shrubland | 0.073 | 0.066 | 0.081 | Negligible |
| Rate | Grassland | Water | 0.173 | 0.153 | 0.193 | Small |
| Rate | Grassland | Wetland | 0.161 | 0.152 | 0.169 | Small |
| Rate | Shrubland | Water | 0.095 | 0.076 | 0.115 | Negligible |
| Rate | Shrubland | Wetland | 0.086 | 0.079 | 0.093 | Negligible |
| Rate | Water | Wetland | -0.006 | -0.027 | 0.015 | Negligible |
| *Magnitude is “Negligible” when abs(Cliff_Delta) <0.147; “Small” when 0.147≤abs(Cliff Delta)<0.33; “Medium” when 0.33≤abs(Cliff Delta)<0.474; and “Large” when abs(Cliff Delta) ≥ 0.474. | | | | | | |

**
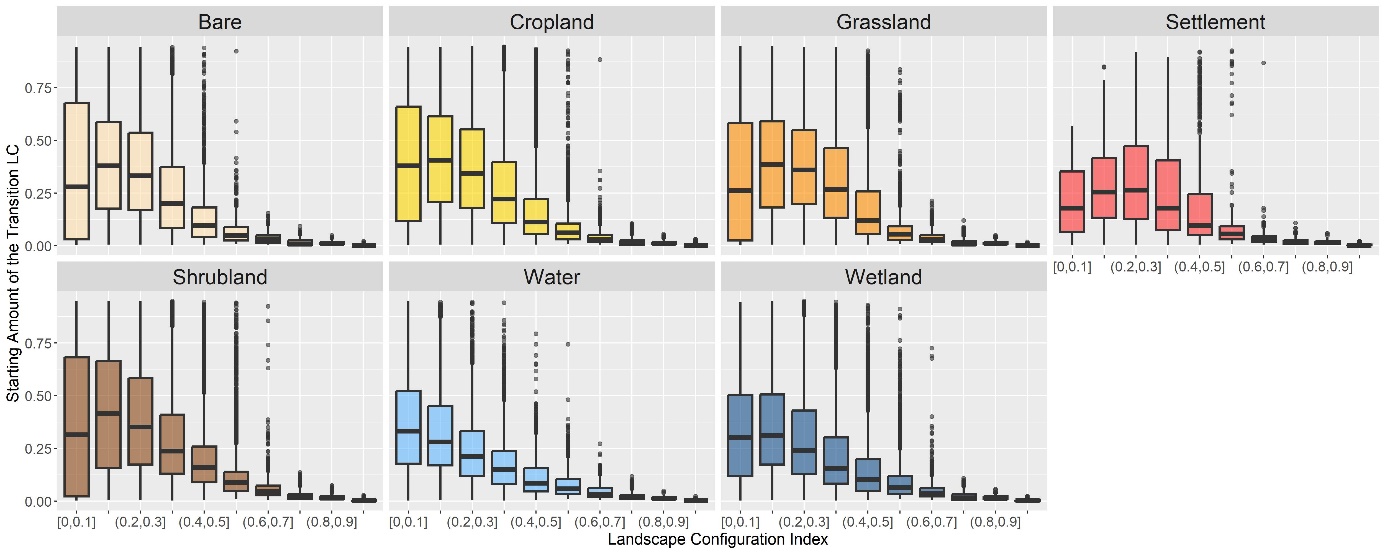
**

Figure 1: Boxplots depicting the distribution of the starting LC amount for different levels of starting LCI for grids with persistent forest loss. The LCI values, depicted on the x-axis, have been binned using the following thresholds: [0, 0.1, 0.2, 0.3, 0.4, 0.5, 0.6, 0.7, 0.8, 0.9, 1.0].

**
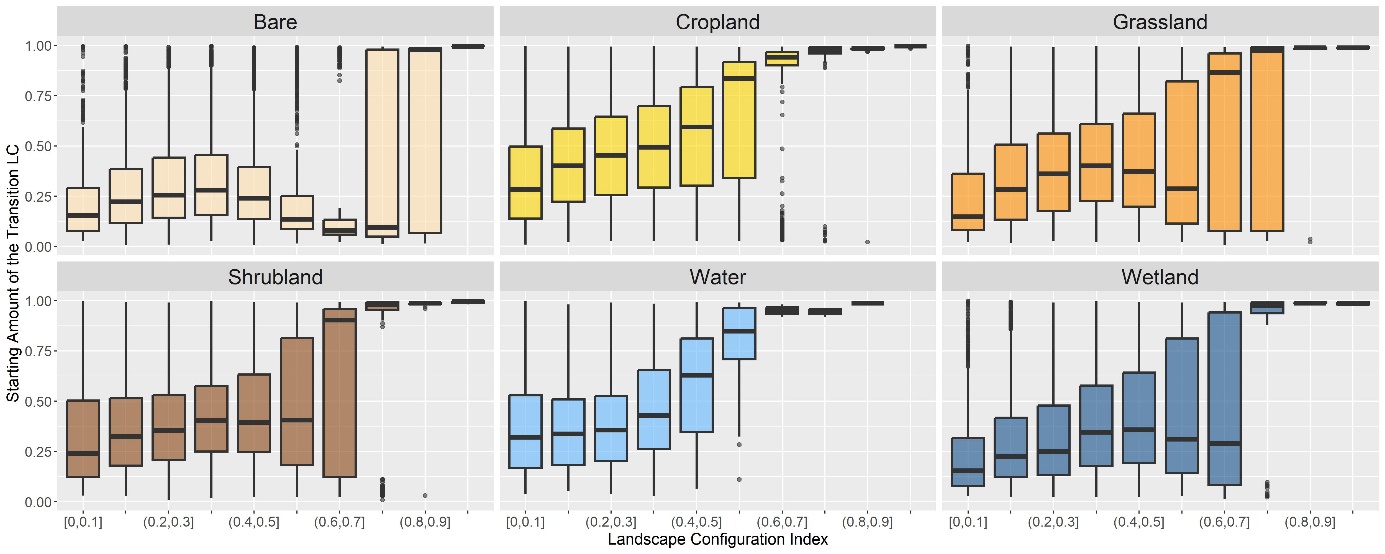
**

Figure 2: Boxplots depicting the distribution of the starting LC amount for different levels of starting LCI for grids with persistent forest gain. The LCI values, depicted on the x-axis, have been binned using the following thresholds: [0, 0.1, 0.2, 0.3, 0.4, 0.5, 0.6, 0.7, 0.8, 0.9, 1.0].

**GAM Statistics for the Effect of Starting Amount on the Mean Amount of Forest Loss**

**
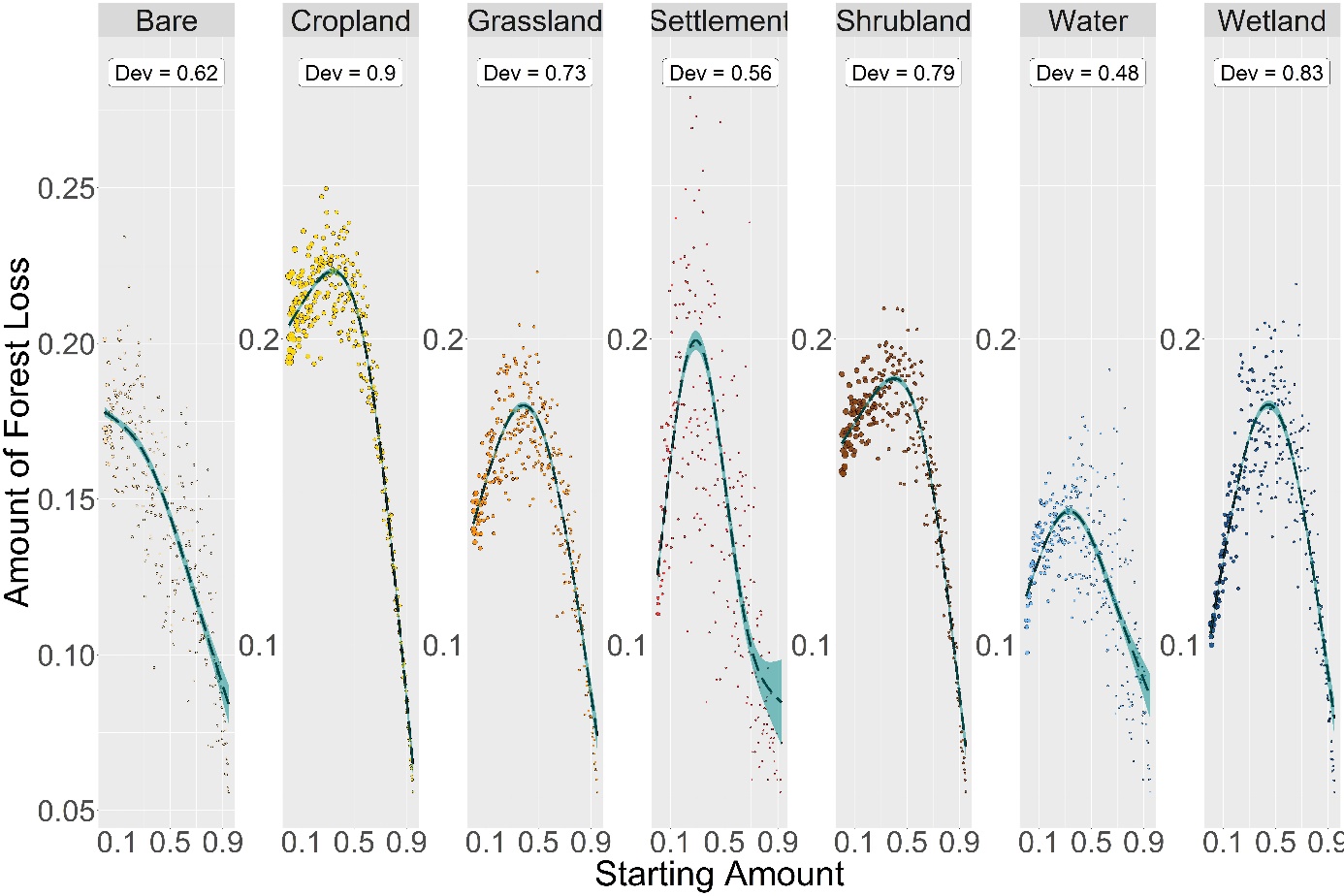
**

Figure 3: GAMs for the mean effect of the starting amount (expressed as a percentage of the grid size) on the mean amount of forest loss. Dev = the proportion of deviance explained.

| **Table 3**: GAM Statistics for Effects of Starting Amount on Mean Amount of Forest Loss | | | | | | | |
| --- | --- | --- | --- | --- | --- | --- | --- |
| **Land-Cover** | **Number of Grids** | **EDF** | **Deviance** | **Fitted Values: Minimum** | **Fitted Values: Maximum** | **Mean of the Real Rate of Change** | **Mean of Absolute Rate of Change** |
| Bare | 10770 | 2.639 | 0.617 | 0.944 | 0.003 | -0.1 | 0.1 |
| Cropland | 93752 | 2.99 | 0.896 | 0.948 | 0.34 | -0.152 | 0.189 |
| Grassland | 29969 | 2.979 | 0.734 | 0.948 | 0.386 | -0.073 | 0.155 |
| Settlement | 3484 | 2.97 | 0.564 | 0.926 | 0.287 | -0.045 | 0.219 |
| Shrubland | 67101 | 2.987 | 0.787 | 0.948 | 0.401 | -0.105 | 0.15 |
| Water | 14771 | 2.926 | 0.478 | 0.948 | 0.33 | -0.034 | 0.093 |
| Wetland | 26327 | 2.981 | 0.832 | 0.948 | 0.444 | -0.029 | 0.188 |
| EDF = Effective Degrees of Freedom; Deviance = The proportion of the null deviance explained by the model | | | | | | | |

**GAM Statistics for the Effect of Starting Amount on the Mean Rate of Forest Loss**

**
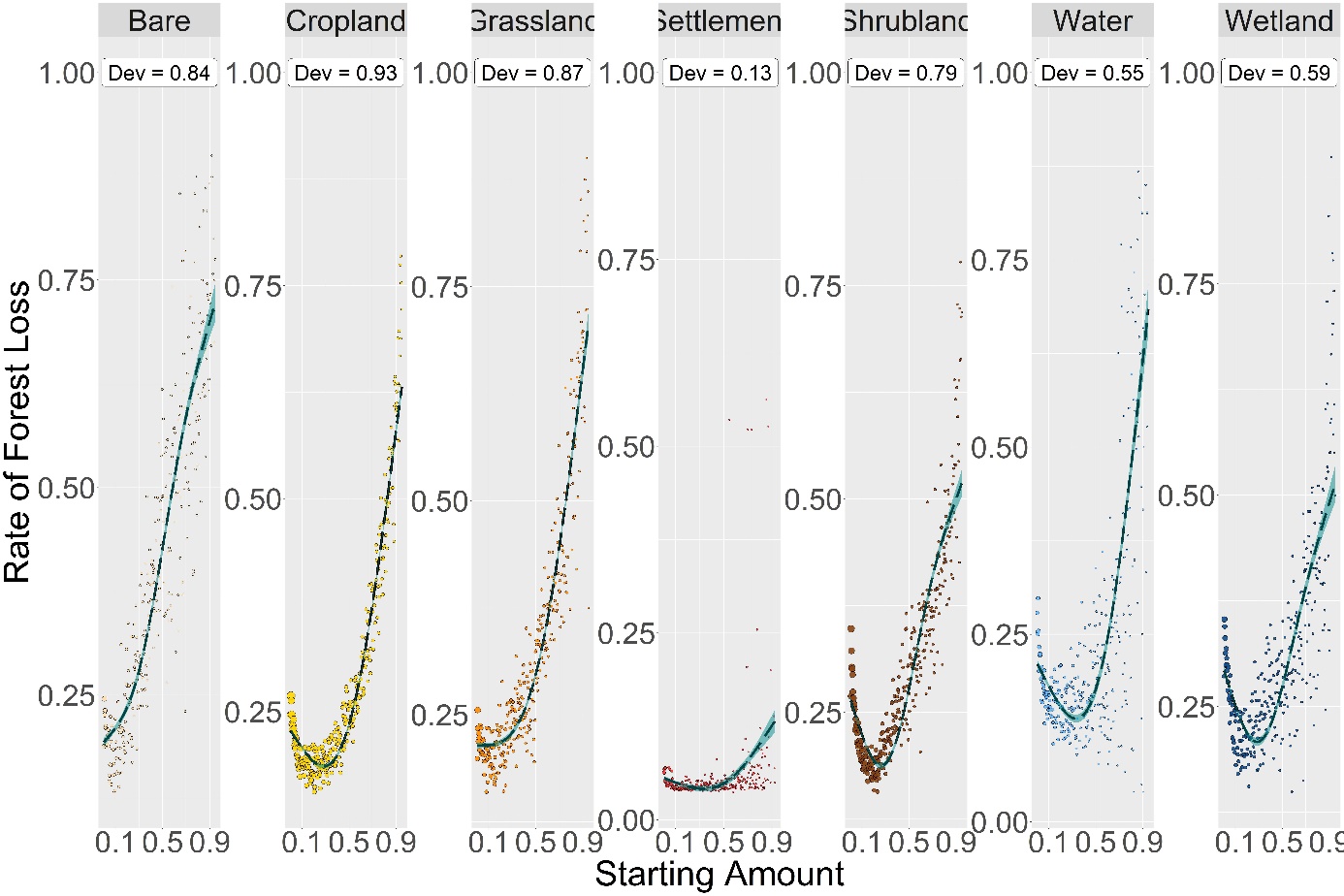
**

Figure 4: GAMs for the mean effect of the starting amount (expressed as a percentage of the grid size) on the mean rate of forest loss. Dev = the proportion of deviance explained.

| **Table 4**: GAM Statistics for Effects of Starting Amount on Mean Rate of Forest Loss | | | | | | | |
| --- | --- | --- | --- | --- | --- | --- | --- |
| **Land-Cover** | **Number of Grids** | **EDF** | **Deviance** | **Fitted Values: Minimum** | **Fitted Values: Maximum** | **Mean of Average Rate of Change** | **Mean of Absolute Rate of Change** |
| Bare | 10770 | 2.903 | 0.844 | 0.003 | 0.944 | 0.562 | 0.562 |
| Cropland | 93752 | 2.99 | 0.931 | 0.284 | 0.948 | 0.427 | 0.515 |
| Grassland | 29969 | 2.966 | 0.875 | 0.003 | 0.948 | 0.52 | 0.52 |
| Settlement | 3484 | 2.674 | 0.132 | 0.333 | 0.926 | 0.075 | 0.107 |
| Shrubland | 67101 | 2.979 | 0.79 | 0.262 | 0.948 | 0.269 | 0.431 |
| Water | 14771 | 2.954 | 0.55 | 0.333 | 0.948 | 0.498 | 0.653 |
| Wetland | 26327 | 2.954 | 0.593 | 0.284 | 0.948 | 0.233 | 0.412 |
| EDF = Effective Degrees of Freedom; Deviance = The proportion of the null deviance explained by the model | | | | | | | |

**GAM Statistics for the Effect of Starting Configuration on the Mean Amount of Forest Loss**


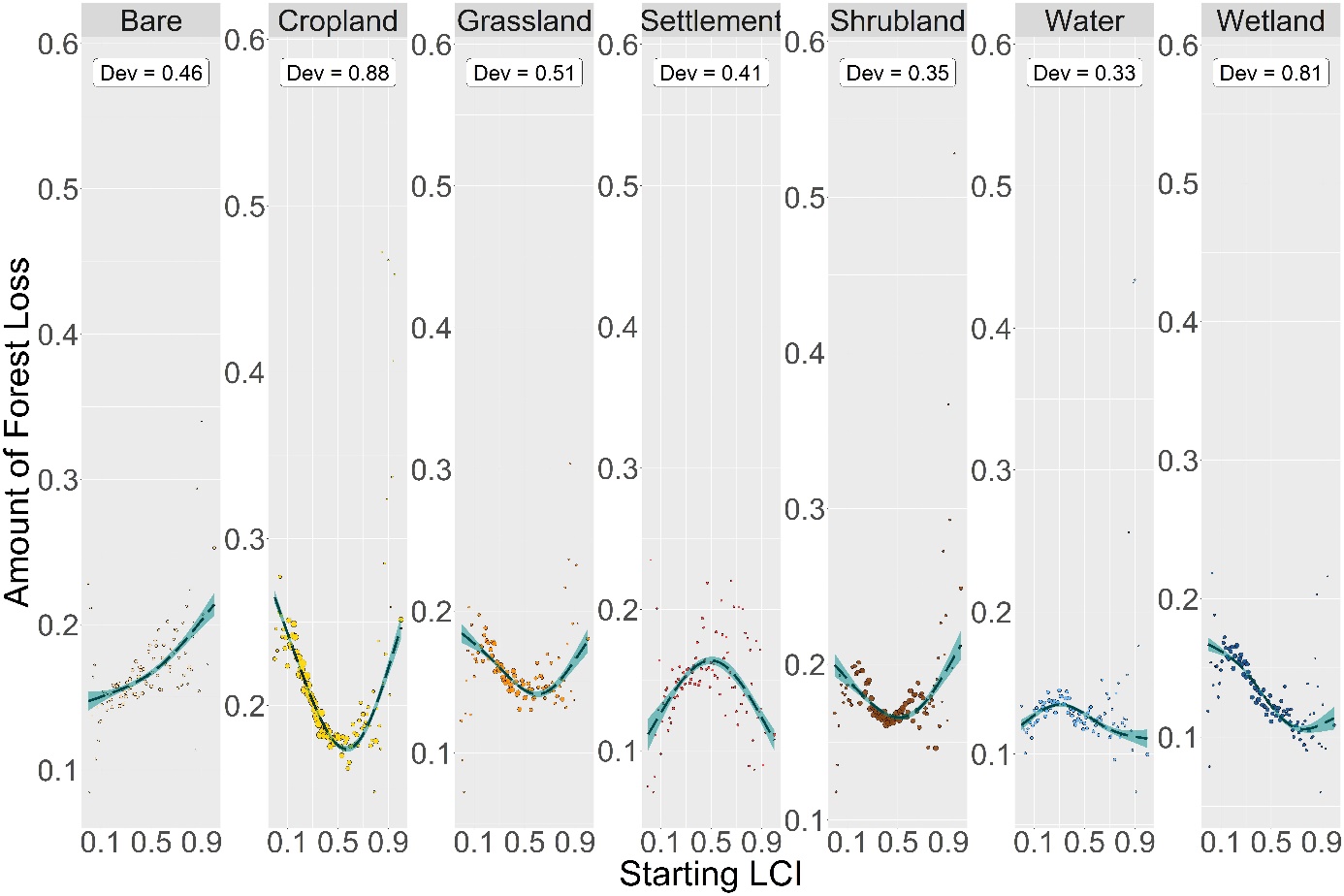


Figure 5: GAMs for the mean effect of the starting configuration on the mean amount of forest loss. Dev = the proportion of deviance explained.

| **Table 5**: GAM Statistics for Effects of Starting Configuration on Mean Amount of Forest Loss | | | | | | | |
| --- | --- | --- | --- | --- | --- | --- | --- |
| **Land-Cover** | **Number of Grids** | **EDF** | **Deviance** | **Fitted Values: Minimum** | **Fitted Values: Maximum** | **Mean of Average Rate of Change*** | **Mean of Absolute Rate of Change*** |
| Bare | 10770 | 2.177 | 0.464 | 0 | 1 | 0.044 | 0.044 |
| Cropland | 93752 | 2.969 | 0.882 | 0.57 | 0 | -0.151 | 0.153 |
| Grassland | 29969 | 2.844 | 0.506 | 0.6 | 0 | -0.072 | 0.072 |
| Settlement | 3484 | 2.773 | 0.412 | 1 | 0.5 | 0.079 | 0.09 |
| Shrubland | 67101 | 2.795 | 0.353 | 0.51 | 1 | -0.053 | 0.059 |
| Water | 14771 | 2.811 | 0.33 | 1 | 0.31 | 0.003 | 0.044 |
| Wetland | 26327 | 2.857 | 0.807 | 0.76 | 0 | -0.092 | 0.092 |
| EDF = Effective Degrees of Freedom; Deviance = The proportion of the null deviance explained by the model  *The metrics for the mean of the real and absolute average rate of change have been calculated over the LCI interval between 0 and 0.6 only. | | | | | | | |

**GAM Statistics for the Effect of Starting Configuration on the Mean Rate of Forest Loss**


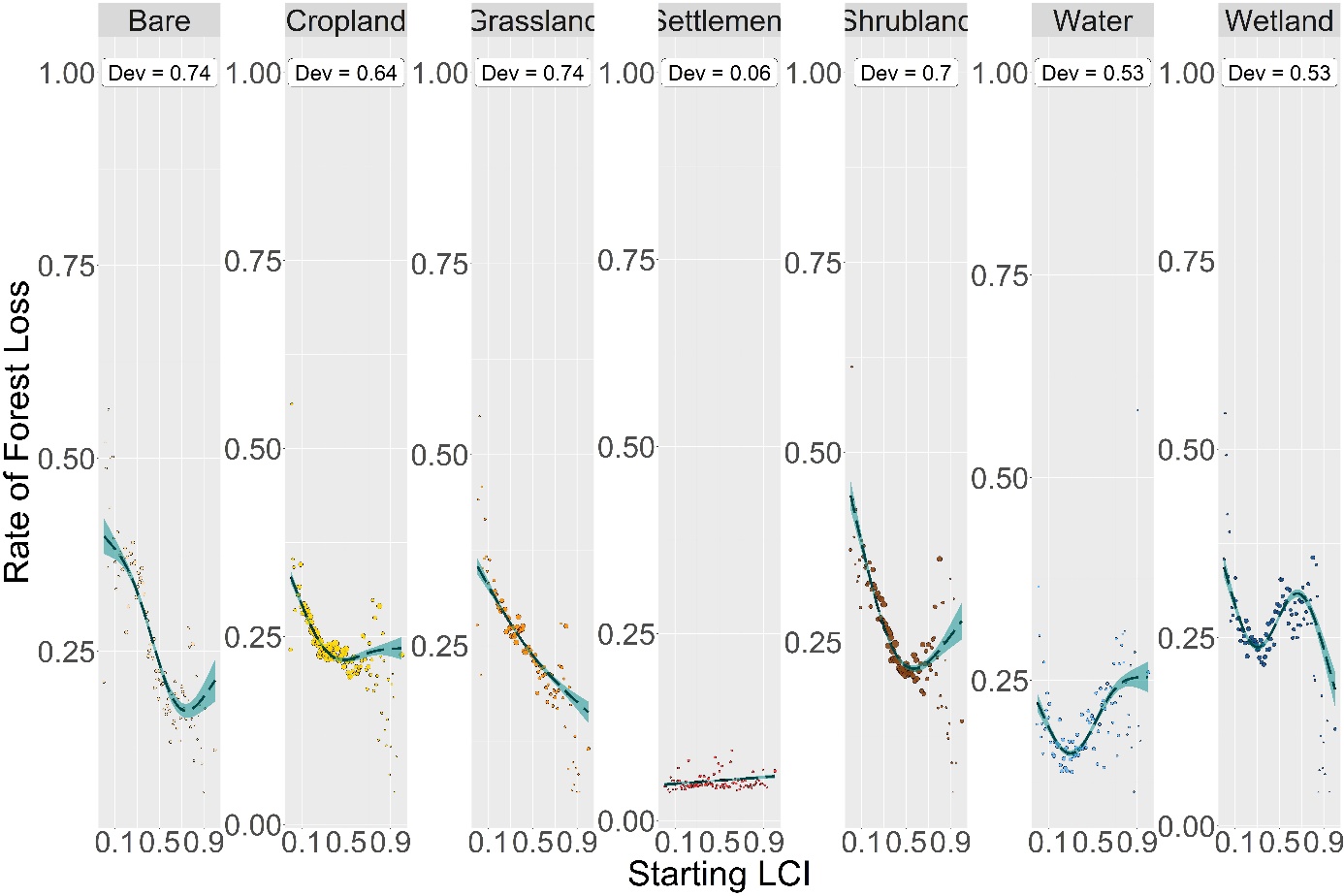


Figure 6: GAMs for the mean effect of the starting configuration on the mean rate of forest loss. Dev = the proportion of deviance explained.

| **Table 6**: GAM Statistics for Effects of Starting Configuration on Mean Rate of Forest Loss | | | | | | | |
| --- | --- | --- | --- | --- | --- | --- | --- |
| **Land-Cover** | **Number of Grids** | **EDF** | **Deviance** | **Fitted Values: Minimum** | **Fitted Values: Maximum** | **Mean of Average Rate of Change*** | **Mean of Absolute Rate of Change*** |
| Bare | 10770 | 2.861 | 0.737 | 0.74 | 0 | -0.341 | 0.341 |
| Cropland | 93752 | 2.885 | 0.637 | 0.48 | 0 | -0.178 | 0.191 |
| Grassland | 29969 | 2.118 | 0.741 | 1 | 0 | -0.224 | 0.224 |
| Settlement | 3484 | 1 | 0.059 | 0 | 1 | 0.011 | 0.011 |
| Shrubland | 67101 | 2.883 | 0.697 | 0.57 | 0 | -0.379 | 0.381 |
| Water | 14771 | 2.922 | 0.535 | 0.3 | 1 | -0.007 | 0.204 |
| Wetland | 26327 | 2.97 | 0.526 | 1 | 0 | -0.067 | 0.288 |
| EDF = Effective Degrees of Freedom; Deviance = The proportion of the null deviance explained by the model  *The metrics for the mean of the real and absolute average rate of change have been calculated over the LCI interval between 0 and 0.6 only. | | | | | | | |

**GAM Statistics for the Effect of Starting Amount on the Mean Amount of Forest Gain**

**
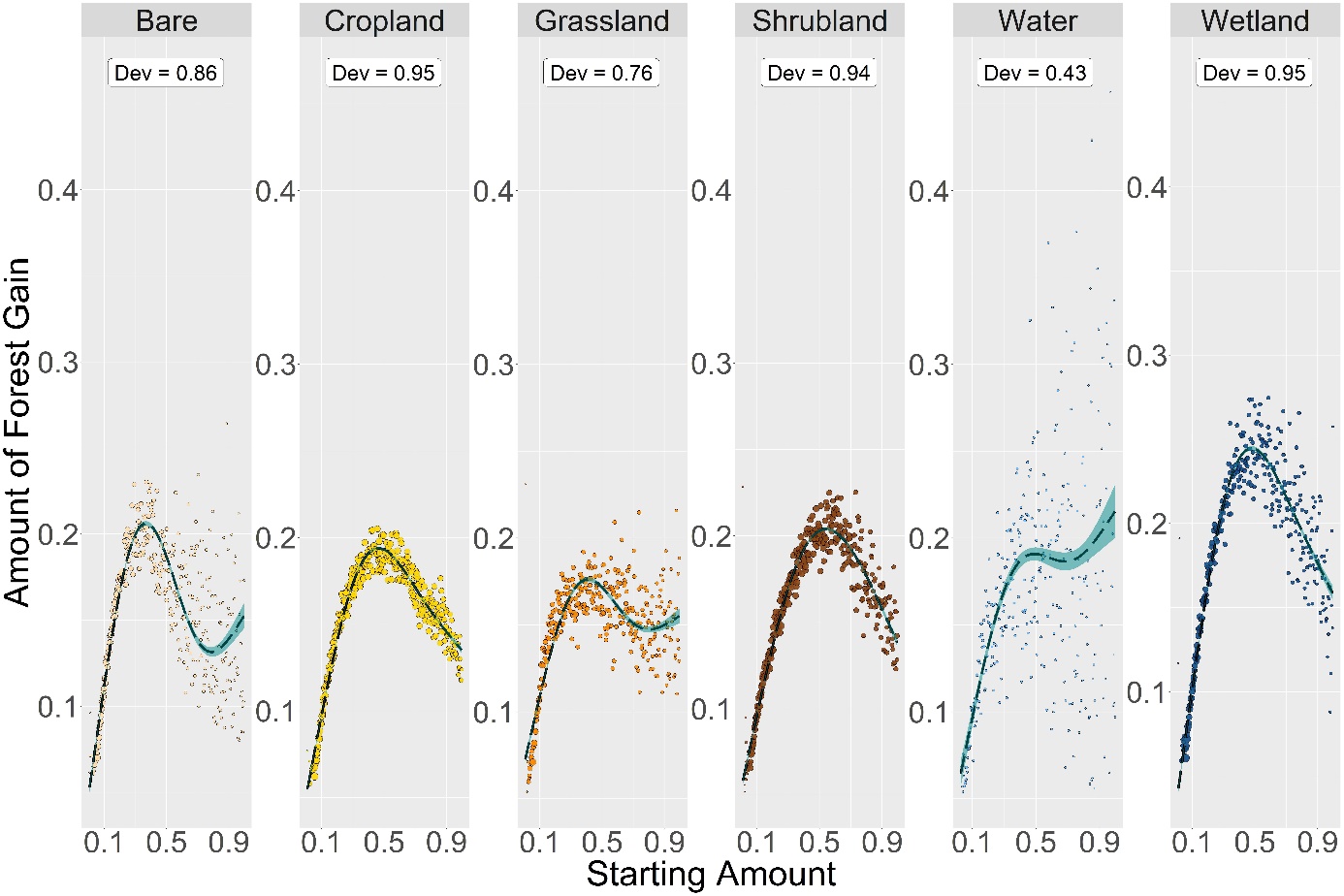
**

Figure 7: GAMs for the mean effect of the starting amount (expressed as a percentage of the grid size) on the mean amount of forest gain. Dev = the proportion of deviance explained.

| **Table 7**: GAM Statistics for Effects of Starting Amount on Mean Amount of Forest Gain | | | | | | | |
| --- | --- | --- | --- | --- | --- | --- | --- |
| **Land-Cover** | **Number of Grids** | **EDF** | **Deviance** | **Fitted Values: Minimum** | **Fitted Values: Maximum** | **Mean of Average Rate of Change** | **Mean of Absolute Rate of Change** |
| Bare | 27019 | 2.995 | 0.862 | 0.006 | 0.367 | 0.099 | 0.25 |
| Cropland | 84722 | 2.998 | 0.948 | 0.009 | 0.466 | 0.077 | 0.197 |
| Grassland | 30413 | 2.99 | 0.757 | 0.006 | 0.41 | 0.08 | 0.139 |
| Shrubland | 71533 | 2.995 | 0.942 | 0.009 | 0.54 | 0.078 | 0.212 |
| Water | 3014 | 2.897 | 0.429 | 0.028 | 0.997 | 0.153 | 0.163 |
| Wetland | 34621 | 2.995 | 0.948 | 0.012 | 0.478 | 0.116 | 0.291 |
| EDF = Effective Degrees of Freedom; Deviance = The proportion of the null deviance explained by the model | | | | | | | |

**GAM Statistics for the Effect of Starting Amount on the Mean Rate of Forest Gain**

**
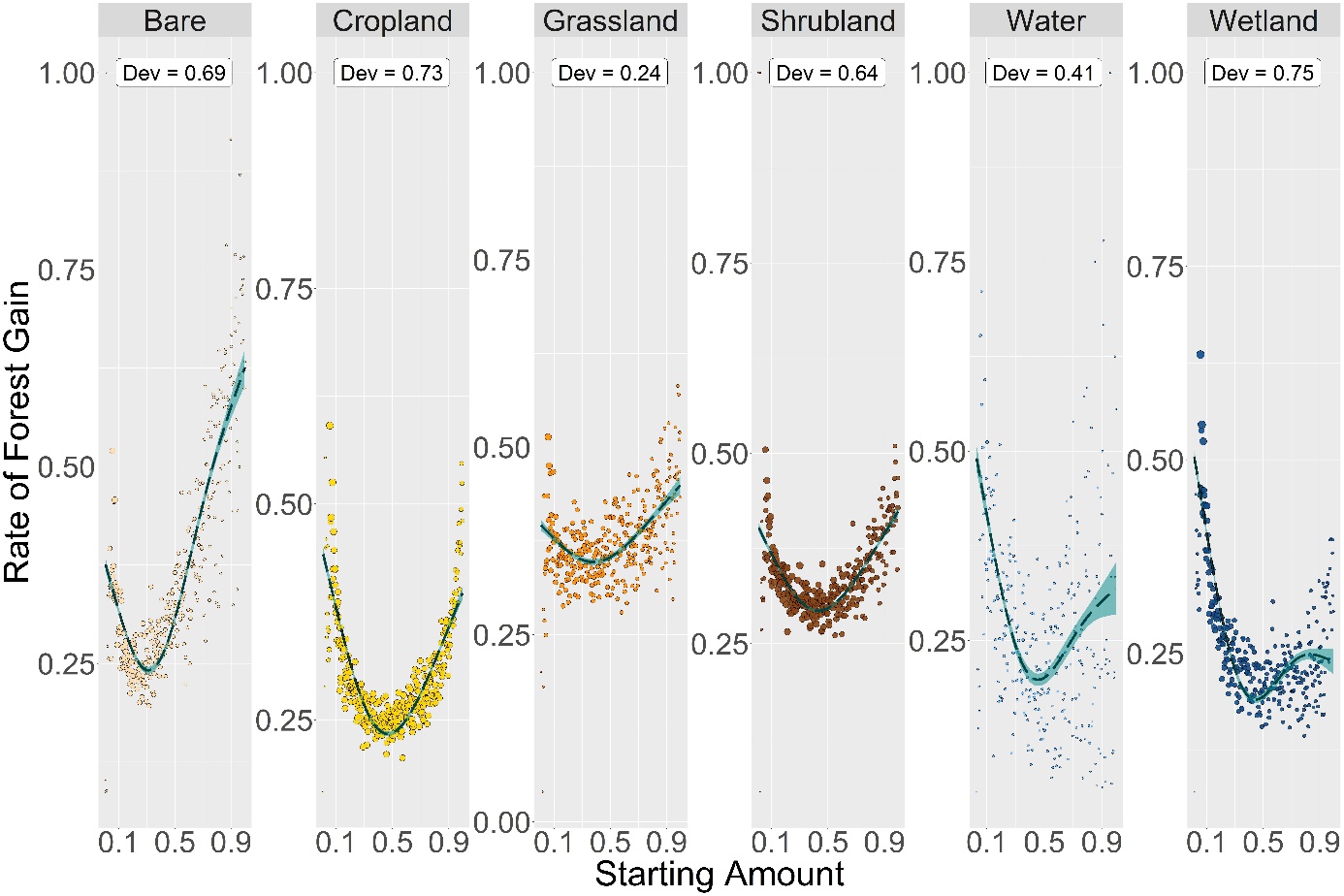
**

Figure 8: GAMs for the mean effect of the starting amount (expressed as a percentage of the grid size) on the mean rate of forest gain. Dev = the proportion of deviance explained.

| **Table 8**: GAM Statistics for Effects of Starting Amount on Mean Rate of Forest Gain | | | | | | | |
| --- | --- | --- | --- | --- | --- | --- | --- |
| **Land-Cover** | **Number of Grids** | **EDF** | **Deviance** | **Fitted Values: Minimum** | **Fitted Values: Maximum** | **Mean of Average Rate of Change** | **Mean of Absolute Rate of Change** |
| Bare | 27019 | 2.974 | 0.695 | 0.312 | 0.997 | 0.255 | 0.523 |
| Cropland | 84722 | 2.983 | 0.729 | 0.469 | 0.009 | -0.039 | 0.373 |
| Grassland | 30413 | 2.83 | 0.239 | 0.38 | 0.997 | 0.056 | 0.153 |
| Shrubland | 71533 | 2.972 | 0.64 | 0.432 | 0.997 | 0.024 | 0.241 |
| Water | 3014 | 2.917 | 0.405 | 0.457 | 0.028 | -0.179 | 0.427 |
| Wetland | 34621 | 2.979 | 0.751 | 0.444 | 0.012 | -0.265 | 0.384 |
| EDF = Effective Degrees of Freedom; Deviance = The proportion of the null deviance explained by the model | | | | | | | |

**GAM Statistics for the Effect of Starting Configuration on the Mean Amount of Forest Gain**


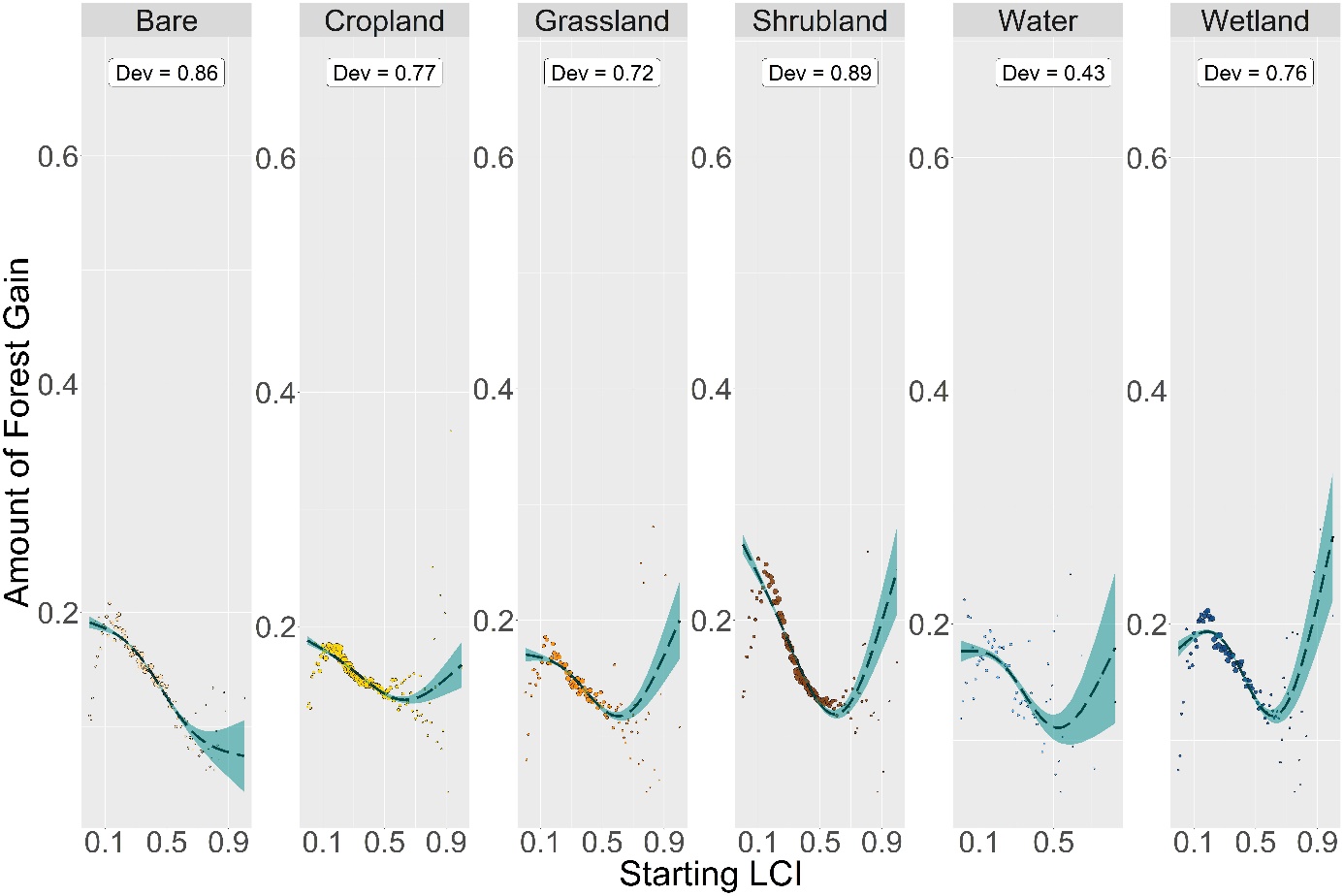


Figure 9: GAMs for the mean effect of the starting configuration on the mean amount of forest gain. Dev = the proportion of deviance explained.

| **Table 9**: GAM Statistics for Effects of Starting Configuration on Mean Amount of Forest Gain | | | | | | | |
| --- | --- | --- | --- | --- | --- | --- | --- |
| **Land-Cover** | **Number of Grids** | **EDF** | **Deviance** | **Fitted Values: Minimum** | **Fitted Values: Maximum** | **Mean of Average Rate of Change*** | **Mean of Absolute Rate of Change*** |
| Bare | 27019 | 2.713 | 0.864 | 1 | 0 | -0.142 | 0.142 |
| Cropland | 84722 | 2.79 | 0.771 | 0.63 | 0 | -0.084 | 0.084 |
| Grassland | 30413 | 2.878 | 0.72 | 0.6 | 1 | -0.089 | 0.089 |
| Shrubland | 71533 | 2.915 | 0.886 | 0.6 | 0 | -0.244 | 0.244 |
| Water | 3014 | 2.687 | 0.435 | 0.53 | 0.83 | -0.11 | 0.121 |
| Wetland | 34621 | 2.929 | 0.761 | 0.61 | 1 | -0.096 | 0.144 |
| EDF = Effective Degrees of Freedom; Deviance = The proportion of the null deviance explained by the model  *The metrics for the mean of the real and absolute average rate of change have been calculated over the LCI interval between 0 and 0.6 only. | | | | | | | |

**GAM Statistics for the Effect of Starting Configuration on the Mean Rate of Forest Gain**


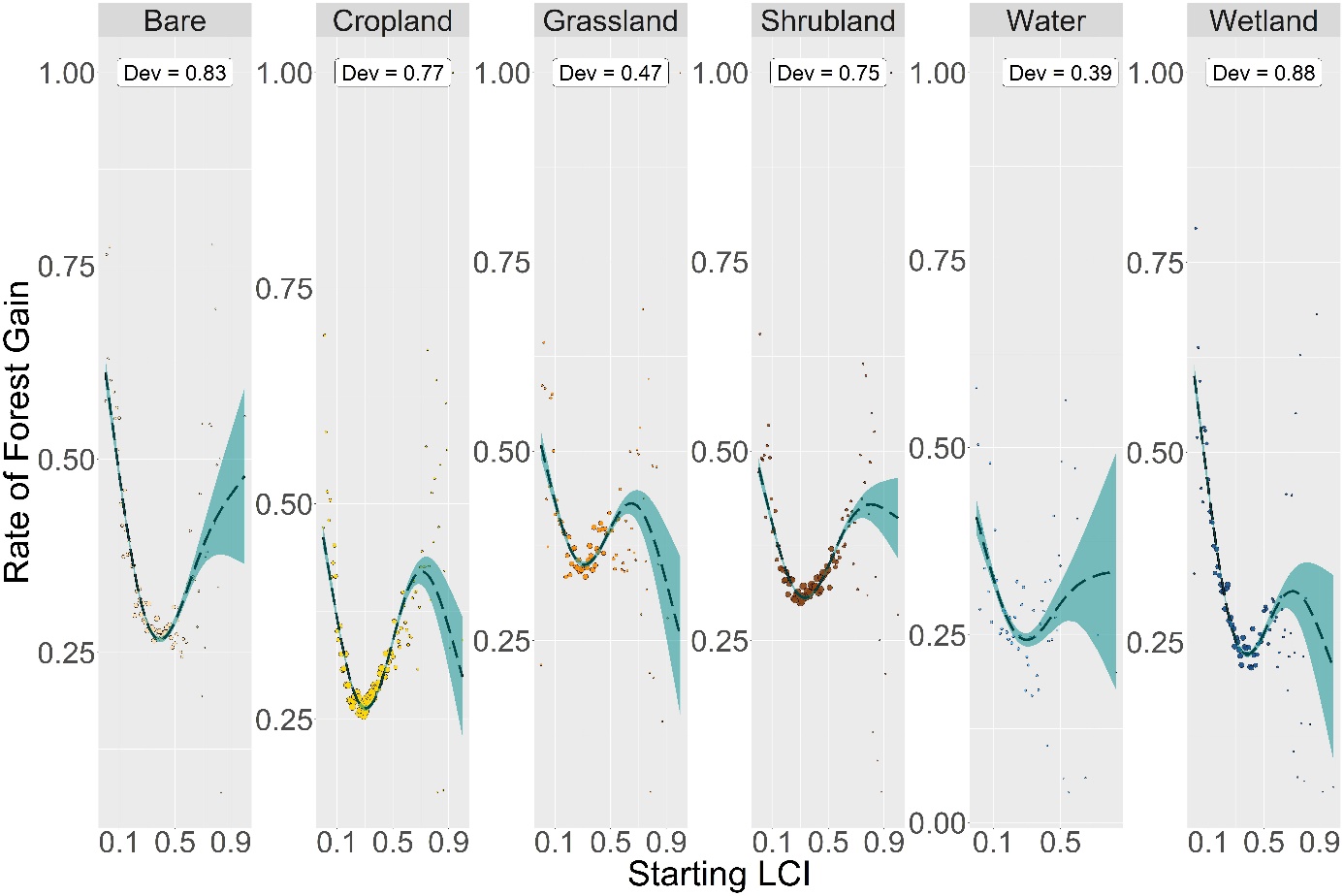


Figure 10: GAMs for the mean effect of the starting configuration on the mean rate of forest gain. Dev = the proportion of deviance explained.

| **Table 10**: GAM Statistics for Effects of Starting Configuration on Mean Rate of Forest Gain | | | | | | | |
| --- | --- | --- | --- | --- | --- | --- | --- |
| **Land-Cover** | **Number of Grids** | **EDF** | **Deviance** | **Fitted Values: Minimum** | **Fitted Values: Maximum** | **Mean of Average Rate of Change*** | **Mean of Absolute Rate of Change*** |
| Bare | 27019 | 2.912 | 0.832 | 0.39 | 0 | -0.458 | 0.696 |
| Cropland | 84722 | 2.97 | 0.771 | 0.3 | 0 | -0.107 | 0.554 |
| Grassland | 30413 | 2.903 | 0.473 | 1 | 0 | -0.135 | 0.392 |
| Shrubland | 71533 | 2.956 | 0.75 | 0.33 | 0 | -0.147 | 0.425 |
| Water | 3014 | 2.547 | 0.391 | 0.3 | 0 | -0.171 | 0.403 |
| Wetland | 34621 | 2.931 | 0.881 | 1 | 0 | -0.503 | 0.721 |
| EDF = Effective Degrees of Freedom; Deviance = The proportion of the null deviance explained by the model  *The metrics for the mean of the real and absolute average rate of change have been calculated over the LCI interval between 0 and 0.6 only. | | | | | | | |

**Appendix IV**

**Multi-Scale GAM Statistics for the Effect of Starting Amount on the Mean Amount of Forest Loss**

**
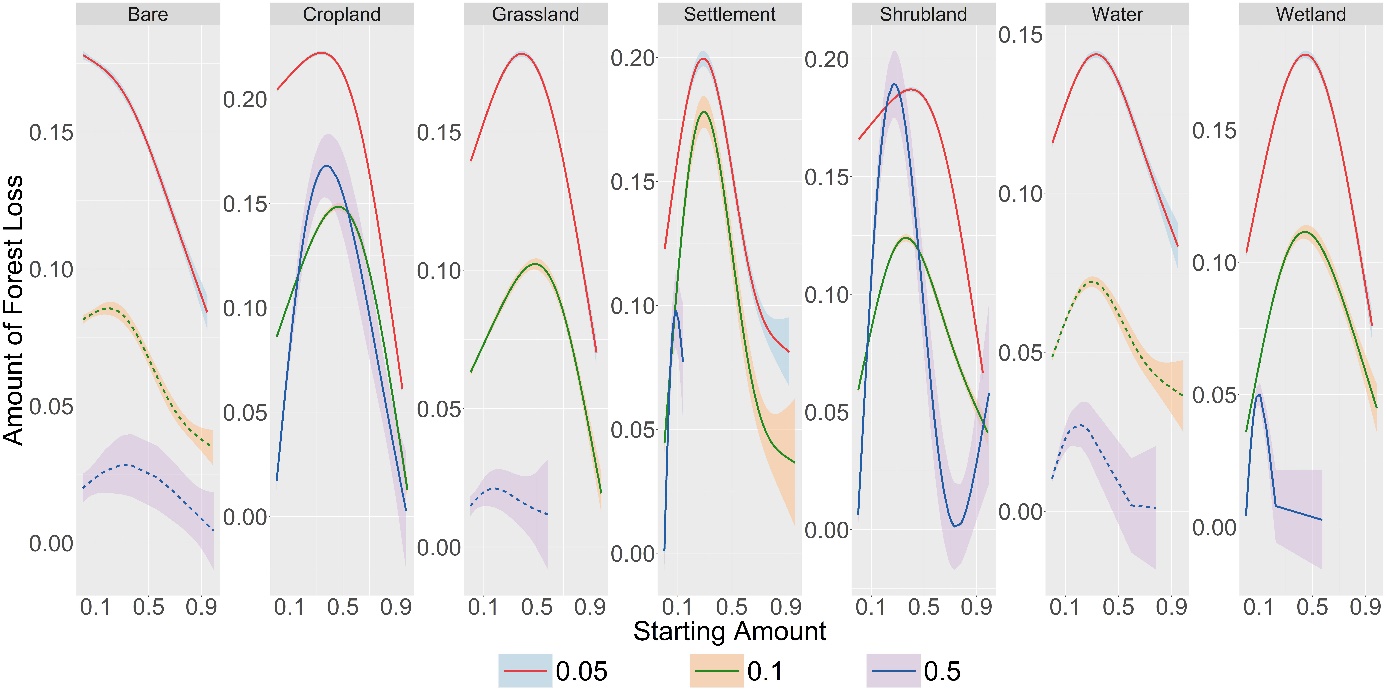
**

Figure 1: GAMs for the mean effect of the starting amount on the mean amount of forest loss for the 0.05, 0.1 and 0.5 degree-decimal resolutions (which are equivalent to the 3, 6 and 30 arc-minute resolutions, respectively)

| **Table 1**: Multi-Scale GAM Statistics for Effects of Starting Amount on Mean Amount of Forest Loss | | | | | | | | |
| --- | --- | --- | --- | --- | --- | --- | --- | --- |
| **Grid Size** | **Land-Cover** | **Number of Grids** | **EDF** | **Deviance** | **Fitted Values: Minimum** | **Fitted Values: Maximum** | **Mean of the Real Rate of Change** | **Mean of Absolute Rate of Change** |
| 3 arc-minute | Bare | 10770 | 2.639 | 0.617 | 0.944 | 0.003 | -0.1 | 0.1 |
| 3 arc-minute | Cropland | 93752 | 2.99 | 0.896 | 0.948 | 0.34 | -0.152 | 0.189 |
| 3 arc-minute | Grassland | 29969 | 2.979 | 0.734 | 0.948 | 0.386 | -0.073 | 0.155 |
| 3 arc-minute | Settlement | 3484 | 2.97 | 0.564 | 0.926 | 0.287 | -0.045 | 0.219 |
| 3 arc-minute | Shrubland | 67101 | 2.987 | 0.787 | 0.948 | 0.401 | -0.105 | 0.15 |
| 3 arc-minute | Water | 14771 | 2.926 | 0.478 | 0.948 | 0.33 | -0.034 | 0.093 |
| 3 arc-minute | Wetland | 26327 | 2.981 | 0.832 | 0.948 | 0.444 | -0.029 | 0.188 |
| 6 arc-minute | Bare | 3586 | 2.765 | 0.155 | 0.986 | 0.194 | -0.044 | 0.054 |
| 6 arc-minute | Cropland | 28395 | 2.984 | 0.51 | 0.986 | 0.465 | -0.074 | 0.201 |
| 6 arc-minute | Grassland | 9212 | 2.944 | 0.229 | 0.984 | 0.486 | -0.031 | 0.117 |
| 6 arc-minute | Settlement | 1124 | 2.959 | 0.439 | 0.969 | 0.292 | 0.2 | 0.4 |
| 6 arc-minute | Shrubland | 23387 | 2.98 | 0.491 | 0.986 | 0.362 | -0.018 | 0.15 |
| 6 arc-minute | Water | 6423 | 2.855 | 0.162 | 0.985 | 0.301 | 0.011 | 0.067 |
| 6 arc-minute | Wetland | 11356 | 2.937 | 0.49 | 0.001 | 0.443 | 0.039 | 0.148 |
| 30 arc-minute | Bare | 82 | 1.62 | 0.057 | 0.992 | 0.305 | 0.004 | 0.039 |
| 30 arc-minute | Cropland | 799 | 2.785 | 0.351 | 0.975 | 0.391 | 0.344 | 0.486 |
| 30 arc-minute | Grassland | 153 | 1.572 | 0.023 | 0.584 | 0.178 | 0.029 | 0.042 |
| 30 arc-minute | Settlement | 38 | 2.286 | 0.715 | 0 | 0.087 | 1.085 | 1.225 |
| 30 arc-minute | Shrubland | 548 | 2.95 | 0.437 | 0.733 | 0.269 | 0.58 | 0.697 |
| 30 arc-minute | Water | 361 | 2.341 | 0.146 | 0.782 | 0.216 | 0.115 | 0.12 |
| 30 arc-minute | Wetland | 401 | 2.936 | 0.697 | 0.574 | 0.104 | 0.537 | 0.585 |
| EDF = Effective Degrees of Freedom; Deviance = The proportion of the null deviance explained by the model | | | | | | | | |

**Multi-Scale GAM Statistics for the Effect of Starting Amount on the Mean Rate of Forest Loss**

**
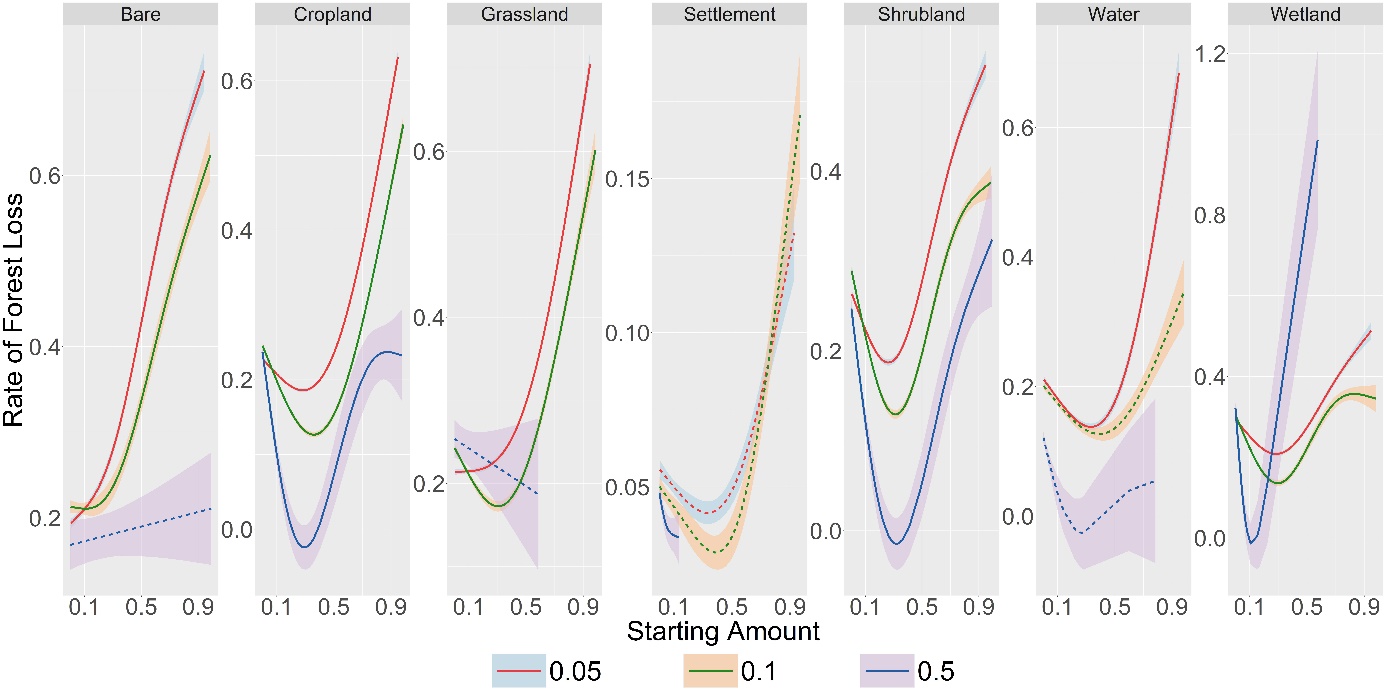
**

Figure 2: GAMs for the mean effect of the starting amount on the mean rate of forest loss for the 0.05, 0.1 and 0.5 degree-decimal resolutions (which are equivalent to the 3, 6 and 30 arc-minute resolutions, respectively)

| **Table 2**: Multi-Scale GAM Statistics for Effects of Starting Amount on Mean Rate of Forest Loss | | | | | | | | |
| --- | --- | --- | --- | --- | --- | --- | --- | --- |
| **Grid Size** | **Land-Cover** | **Number of Grids** | **EDF** | **Deviance** | **Fitted Values: Minimum** | **Fitted Values: Maximum** | **Mean of the Real Rate of Change** | **Mean of Absolute Rate of Change** |
| 3 arc-minute | Bare | 10770 | 2.903 | 0.844 | 0.003 | 0.944 | 0.562 | 0.562 |
| 3 arc-minute | Cropland | 93752 | 2.99 | 0.931 | 0.284 | 0.948 | 0.427 | 0.515 |
| 3 arc-minute | Grassland | 29969 | 2.966 | 0.875 | 0.003 | 0.948 | 0.52 | 0.52 |
| 3 arc-minute | Settlement | 3484 | 2.674 | 0.132 | 0.333 | 0.926 | 0.075 | 0.107 |
| 3 arc-minute | Shrubland | 67101 | 2.979 | 0.79 | 0.262 | 0.948 | 0.269 | 0.431 |
| 3 arc-minute | Water | 14771 | 2.954 | 0.55 | 0.333 | 0.948 | 0.498 | 0.653 |
| 3 arc-minute | Wetland | 26327 | 2.954 | 0.593 | 0.284 | 0.948 | 0.233 | 0.412 |
| 6 arc-minute | Bare | 3586 | 2.751 | 0.334 | 0.106 | 0.986 | 0.388 | 0.393 |
| 6 arc-minute | Cropland | 28395 | 2.984 | 0.605 | 0.36 | 0.986 | 0.3 | 0.542 |
| 6 arc-minute | Grassland | 9212 | 2.939 | 0.33 | 0.305 | 0.984 | 0.325 | 0.48 |
| 6 arc-minute | Settlement | 1124 | 2.768 | 0.12 | 0.39 | 0.969 | 0.025 | 0.106 |
| 6 arc-minute | Shrubland | 23387 | 2.984 | 0.427 | 0.311 | 0.986 | 0.098 | 0.425 |
| 6 arc-minute | Water | 6423 | 2.703 | 0.078 | 0.39 | 0.985 | -0.006 | 0.234 |
| 6 arc-minute | Wetland | 11356 | 2.967 | 0.295 | 0.295 | 0.829 | 0 | 0.435 |
| 30 arc-minute | Bare | 82 | 1 | 0.008 | 0 | 0.992 | 0.043 | 0.043 |
| 30 arc-minute | Cropland | 799 | 2.853 | 0.282 | 0.304 | 0 | -0.762 | 0.923 |
| 30 arc-minute | Grassland | 153 | 1 | 0.007 | 0.584 | 0 | -0.114 | 0.114 |
| 30 arc-minute | Settlement | 38 | 1.619 | 0.257 | 0.137 | 0 | -0.165 | 0.165 |
| 30 arc-minute | Shrubland | 548 | 2.829 | 0.286 | 0.322 | 0.995 | -0.668 | 0.928 |
| 30 arc-minute | Water | 361 | 2.146 | 0.159 | 0.277 | 0 | -0.806 | 0.816 |
| 30 arc-minute | Wetland | 401 | 2.735 | 0.496 | 0.107 | 0.574 | -3.563 | 3.793 |
| EDF = Effective Degrees of Freedom; Deviance = The proportion of the null deviance explained by the model | | | | | | | | |

**Multi-Scale GAM Statistics for the Effect of Starting Configuration on the Mean Amount of Forest Loss**

**
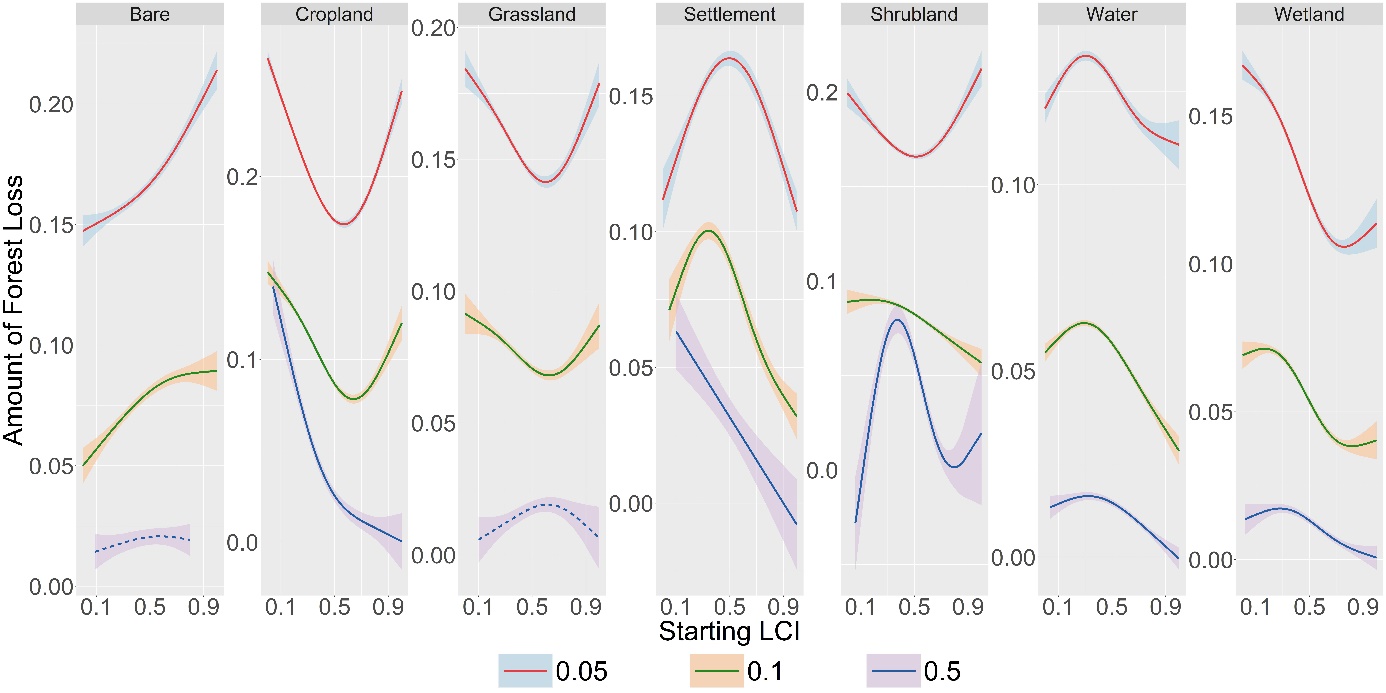
**

Figure 3: GAMs for the mean effect of the starting configuration on the mean amount of forest loss for the 0.05, 0.1 and 0.5 degree-decimal resolutions (which are equivalent to the 3, 6 and 30 arc-minute resolutions, respectively)

| **Table 3**: Multi-Scale GAM Statistics for Effects of Starting Configuration on Mean Amount of Forest Loss | | | | | | | | |
| --- | --- | --- | --- | --- | --- | --- | --- | --- |
| **Grid Size** | **Land-Cover** | **Number of Grids** | **EDF** | **Deviance** | **Fitted Values: Minimum** | **Fitted Values: Maximum** | **Mean of the Real Rate of Change*** | **Mean of Absolute Rate of Change*** |
| 3 arc-minute | Bare | 10770 | 2.177 | 0.464 | 0 | 1 | 0.044 | 0.044 |
| 3 arc-minute | Cropland | 93752 | 2.969 | 0.882 | 0.57 | 0 | -0.151 | 0.153 |
| 3 arc-minute | Grassland | 29969 | 2.844 | 0.506 | 0.6 | 0 | -0.072 | 0.072 |
| 3 arc-minute | Settlement | 3484 | 2.773 | 0.412 | 1 | 0.5 | 0.079 | 0.09 |
| 3 arc-minute | Shrubland | 67101 | 2.795 | 0.353 | 0.51 | 1 | -0.053 | 0.059 |
| 3 arc-minute | Water | 14771 | 2.811 | 0.33 | 1 | 0.31 | 0.003 | 0.044 |
| 3 arc-minute | Wetland | 26327 | 2.857 | 0.807 | 0.76 | 0 | -0.092 | 0.092 |
| 6 arc-minute | Bare | 3586 | 1.947 | 0.271 | 0 | 1 | 0.058 | 0.058 |
| 6 arc-minute | Cropland | 28395 | 2.911 | 0.753 | 0.64 | 0 | -0.115 | 0.115 |
| 6 arc-minute | Grassland | 9212 | 2.603 | 0.273 | 0.63 | 0 | -0.039 | 0.039 |
| 6 arc-minute | Settlement | 1124 | 2.8 | 0.519 | 1 | 0.34 | 0.001 | 0.098 |
| 6 arc-minute | Shrubland | 23387 | 2.215 | 0.388 | 1 | 0.18 | -0.019 | 0.022 |
| 6 arc-minute | Water | 6423 | 2.842 | 0.664 | 1 | 0.29 | -0.005 | 0.032 |
| 6 arc-minute | Wetland | 11356 | 2.839 | 0.745 | 0.82 | 0.17 | -0.039 | 0.047 |
| 30 arc-minute | Bare | 82 | 1.331 | 0.024 | 0.09 | 0.57 | 0.011 | 0.012 |
| 30 arc-minute | Cropland | 799 | 2.603 | 0.568 | 1 | 0.04 | -0.208 | 0.208 |
| 30 arc-minute | Grassland | 153 | 1.94 | 0.071 | 0.1 | 0.61 | 0.026 | 0.026 |
| 30 arc-minute | Settlement | 38 | 1 | 0.206 | 1 | 0.1 | -0.079 | 0.079 |
| 30 arc-minute | Shrubland | 548 | 2.856 | 0.422 | 0.06 | 0.37 | 0.079 | 0.273 |
| 30 arc-minute | Water | 361 | 2.427 | 0.379 | 1 | 0.32 | -0.004 | 0.013 |
| 30 arc-minute | Wetland | 401 | 2.472 | 0.326 | 1 | 0.28 | -0.011 | 0.019 |
| EDF = Effective Degrees of Freedom; Deviance = The proportion of the null deviance explained by the model. *The metrics for the mean of the real and absolute average rate of change have been calculated over the LCI interval between 0 and 0.6 only. | | | | | | | | |

**Multi-Scale GAM Statistics for the Effect of Starting Configuration on the Mean Rate of Forest Loss**

**
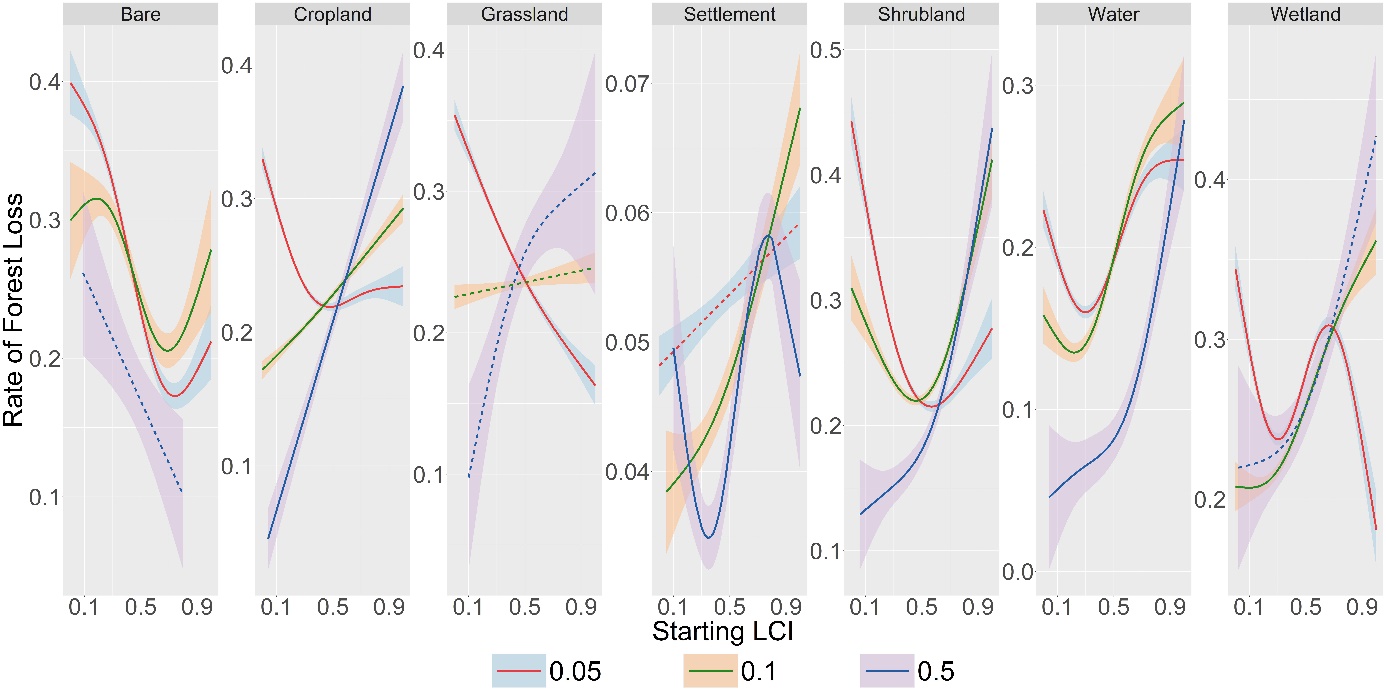
**

Figure 4: GAMs for the mean effect of the starting configuration on the mean rate of forest loss for the 0.05, 0.1 and 0.5 degree-decimal resolutions (which are equivalent to the 3, 6 and 30 arc-minute resolutions, respectively)

| **Table 4**: Multi-Scale GAM Statistics for Effects of Starting Configuration on Mean Rate of Forest Loss | | | | | | | | |
| --- | --- | --- | --- | --- | --- | --- | --- | --- |
| **Grid Size** | **Land-Cover** | **Number of Grids** | **EDF** | **Deviance** | **Fitted Values: Minimum** | **Fitted Values: Maximum** | **Mean of the Real Rate of Change*** | **Mean of Absolute Rate of Change*** |
| 3 arc-minute | Bare | 10770 | 2.861 | 0.737 | 0.74 | 0 | -0.341 | 0.341 |
| 3 arc-minute | Cropland | 93752 | 2.885 | 0.637 | 0.48 | 0 | -0.178 | 0.191 |
| 3 arc-minute | Grassland | 29969 | 2.118 | 0.741 | 1 | 0 | -0.224 | 0.224 |
| 3 arc-minute | Settlement | 3484 | 1 | 0.059 | 0 | 1 | 0.011 | 0.011 |
| 3 arc-minute | Shrubland | 67101 | 2.883 | 0.697 | 0.57 | 0 | -0.379 | 0.381 |
| 3 arc-minute | Water | 14771 | 2.922 | 0.535 | 0.3 | 1 | -0.007 | 0.204 |
| 3 arc-minute | Wetland | 26327 | 2.97 | 0.526 | 1 | 0 | -0.067 | 0.288 |
| 6 arc-minute | Bare | 3586 | 2.703 | 0.296 | 0.69 | 0.19 | -0.142 | 0.194 |
| 6 arc-minute | Cropland | 28395 | 1.407 | 0.539 | 0 | 1 | 0.115 | 0.115 |
| 6 arc-minute | Grassland | 9212 | 1.002 | 0.014 | 0 | 1 | 0.021 | 0.021 |
| 6 arc-minute | Settlement | 1124 | 1.771 | 0.279 | 0.05 | 1 | 0.023 | 0.023 |
| 6 arc-minute | Shrubland | 23387 | 2.811 | 0.438 | 0.46 | 1 | -0.124 | 0.176 |
| 6 arc-minute | Water | 6423 | 2.81 | 0.606 | 0.22 | 1 | 0.116 | 0.193 |
| 6 arc-minute | Wetland | 11356 | 2.598 | 0.724 | 0.1 | 1 | 0.127 | 0.129 |
| 30 arc-minute | Bare | 82 | 1 | 0.056 | 0.8 | 0.09 | -0.226 | 0.226 |
| 30 arc-minute | Cropland | 799 | 1 | 0.407 | 0.04 | 1 | 0.353 | 0.353 |
| 30 arc-minute | Grassland | 153 | 1.653 | 0.119 | 0.1 | 1 | 0.335 | 0.335 |
| 30 arc-minute | Settlement | 38 | 2.82 | 0.553 | 0.35 | 0.78 | 0.01 | 0.044 |
| 30 arc-minute | Shrubland | 548 | 2.225 | 0.332 | 0.06 | 1 | 0.151 | 0.151 |
| 30 arc-minute | Water | 361 | 2.21 | 0.256 | 0.04 | 1 | 0.099 | 0.099 |
| 30 arc-minute | Wetland | 401 | 1.759 | 0.146 | 0.02 | 1 | 0.123 | 0.123 |
| EDF = Effective Degrees of Freedom; Deviance = The proportion of the null deviance explained by the model. *The metrics for the mean of the real and absolute average rate of change have been calculated over the LCI interval between 0 and 0.6 only. | | | | | | | | |

**Multi-Scale GAM Statistics for the Effect of Starting Amount on the Mean Amount of Forest Gain**

**
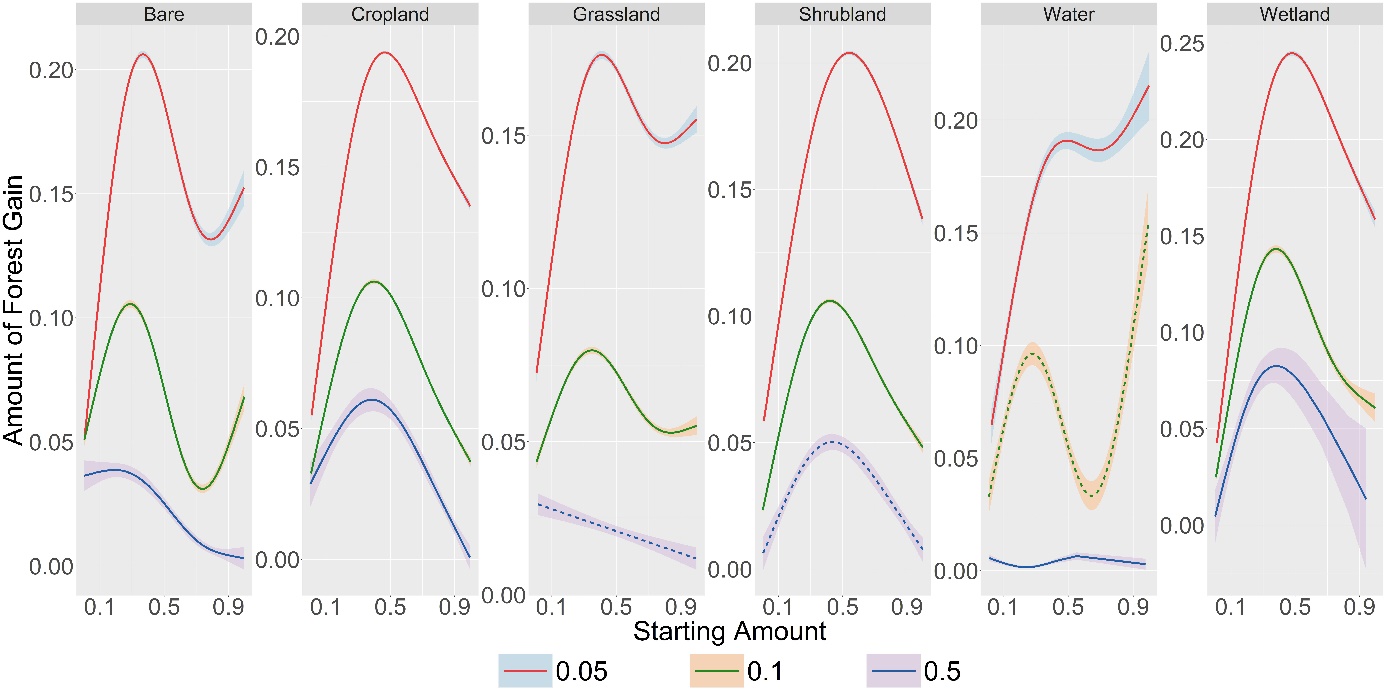
**

Figure 5: GAMs for the mean effect of the starting amount on the mean amount of forest gain for the 0.05, 0.1 and 0.5 degree-decimal resolutions (which are equivalent to the 3, 6 and 30 arc-minute resolutions, respectively)

| **Table 5**: Multi-Scale GAM Statistics for Effects of Starting Amount on Mean Amount of Forest Gain | | | | | | | | |
| --- | --- | --- | --- | --- | --- | --- | --- | --- |
| **Grid Size** | **Land-Cover** | **Number of Grids** | **EDF** | **Deviance** | **Fitted Values: Minimum** | **Fitted Values: Maximum** | **Mean of the Real Rate of Change** | **Mean of Absolute Rate of Change** |
| 3 arc-minute | Bare | 27019 | 2.995 | 0.862 | 0.006 | 0.367 | 0.099 | 0.25 |
| 3 arc-minute | Cropland | 84722 | 2.998 | 0.948 | 0.009 | 0.466 | 0.077 | 0.197 |
| 3 arc-minute | Grassland | 30413 | 2.99 | 0.757 | 0.006 | 0.41 | 0.08 | 0.139 |
| 3 arc-minute | Shrubland | 71533 | 2.995 | 0.942 | 0.009 | 0.54 | 0.078 | 0.212 |
| 3 arc-minute | Water | 3014 | 2.897 | 0.429 | 0.028 | 0.997 | 0.153 | 0.163 |
| 3 arc-minute | Wetland | 34621 | 2.995 | 0.948 | 0.012 | 0.478 | 0.116 | 0.291 |
| 6 arc-minute | Bare | 8877 | 2.994 | 0.465 | 0.738 | 0.288 | 0.013 | 0.166 |
| 6 arc-minute | Cropland | 26413 | 2.995 | 0.685 | 0.006 | 0.399 | 0.004 | 0.143 |
| 6 arc-minute | Grassland | 11712 | 2.979 | 0.223 | 0.006 | 0.349 | 0.012 | 0.066 |
| 6 arc-minute | Shrubland | 26185 | 2.993 | 0.618 | 0.002 | 0.421 | 0.024 | 0.14 |
| 6 arc-minute | Water | 719 | 2.967 | 0.133 | 0.012 | 0.991 | 0.073 | 0.229 |
| 6 arc-minute | Wetland | 7852 | 2.988 | 0.544 | 0.006 | 0.38 | 0.04 | 0.207 |
| 30 arc-minute | Bare | 295 | 2.679 | 0.301 | 0.997 | 0.19 | -0.039 | 0.041 |
| 30 arc-minute | Cropland | 381 | 2.755 | 0.267 | 0.995 | 0.384 | -0.067 | 0.104 |
| 30 arc-minute | Grassland | 237 | 1 | 0.038 | 0.994 | 0.014 | -0.018 | 0.018 |
| 30 arc-minute | Shrubland | 677 | 2.795 | 0.118 | 0.005 | 0.436 | 0.004 | 0.089 |
| 30 arc-minute | Water | 28 | 2.788 | 0.361 | 0.221 | 0.552 | 0.002 | 0.014 |
| 30 arc-minute | Wetland | 76 | 2.527 | 0.243 | 0.001 | 0.383 | 0.159 | 0.193 |
| EDF = Effective Degrees of Freedom; Deviance = The proportion of the null deviance explained by the model | | | | | | | | |

**Multi-Scale GAM Statistics for the Effect of Starting Amount on the Mean Rate of Forest Gain**

**
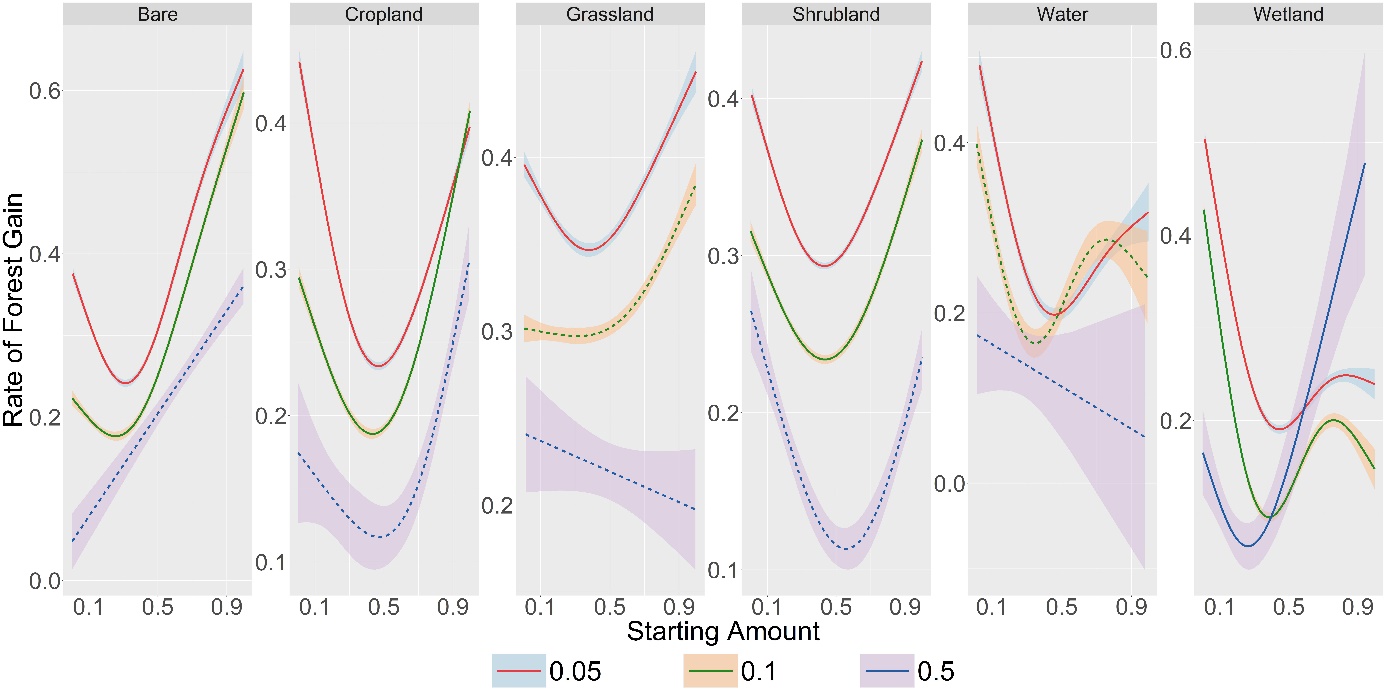
**

Figure 6: GAMs for the mean effect of the starting amount on the mean rate of forest gain for the 0.05, 0.1 and 0.5 degree-decimal resolutions (which are equivalent to the 3, 6 and 30 arc-minute resolutions, respectively)

| **Table 6**: Multi-Scale GAM Statistics for Effects of Starting Amount on Mean Rate of Forest Gain | | | | | | | | |
| --- | --- | --- | --- | --- | --- | --- | --- | --- |
| **Grid Size** | **Land-Cover** | **Number of Grids** | **EDF** | **Deviance** | **Fitted Values: Minimum** | **Fitted Values: Maximum** | **Mean of the Real Rate of Change** | **Mean of Absolute Rate of Change** |
| 3 arc-minute | Bare | 27019 | 2.974 | 0.695 | 0.312 | 0.997 | 0.255 | 0.523 |
| 3 arc-minute | Cropland | 84722 | 2.983 | 0.729 | 0.469 | 0.009 | -0.039 | 0.373 |
| 3 arc-minute | Grassland | 30413 | 2.83 | 0.239 | 0.38 | 0.997 | 0.056 | 0.153 |
| 3 arc-minute | Shrubland | 71533 | 2.972 | 0.64 | 0.432 | 0.997 | 0.024 | 0.241 |
| 3 arc-minute | Water | 3014 | 2.917 | 0.405 | 0.457 | 0.028 | -0.179 | 0.427 |
| 3 arc-minute | Wetland | 34621 | 2.979 | 0.751 | 0.444 | 0.012 | -0.265 | 0.384 |
| 6 arc-minute | Bare | 8877 | 2.943 | 0.491 | 0.255 | 0.999 | 0.37 | 0.465 |
| 6 arc-minute | Cropland | 26413 | 2.973 | 0.474 | 0.434 | 0.999 | 0.115 | 0.33 |
| 6 arc-minute | Grassland | 11712 | 2.57 | 0.054 | 0.314 | 0.998 | 0.084 | 0.093 |
| 6 arc-minute | Shrubland | 26185 | 2.948 | 0.271 | 0.435 | 0.999 | 0.06 | 0.222 |
| 6 arc-minute | Water | 719 | 2.88 | 0.094 | 0.347 | 0.012 | -0.203 | 0.474 |
| 6 arc-minute | Wetland | 7852 | 2.987 | 0.526 | 0.385 | 0.006 | -0.284 | 0.501 |
| 30 arc-minute | Bare | 295 | 1 | 0.139 | 0.002 | 0.997 | 0.315 | 0.315 |
| 30 arc-minute | Cropland | 381 | 2.413 | 0.094 | 0.477 | 0.995 | 0.273 | 0.339 |
| 30 arc-minute | Grassland | 237 | 1 | 0.003 | 0.994 | 0.014 | -0.044 | 0.044 |
| 30 arc-minute | Shrubland | 677 | 2.671 | 0.07 | 0.556 | 0.005 | -0.027 | 0.291 |
| 30 arc-minute | Water | 28 | 1 | 0.014 | 0.975 | 0.014 | -0.125 | 0.125 |
| 30 arc-minute | Wetland | 76 | 2.44 | 0.201 | 0.265 | 0.941 | -0.07 | 0.374 |
| EDF = Effective Degrees of Freedom; Deviance = The proportion of the null deviance explained by the model | | | | | | | | |

**Multi-Scale GAM Statistics for the Effect of Starting Configuration on the Mean Amount of Forest Gain**

**
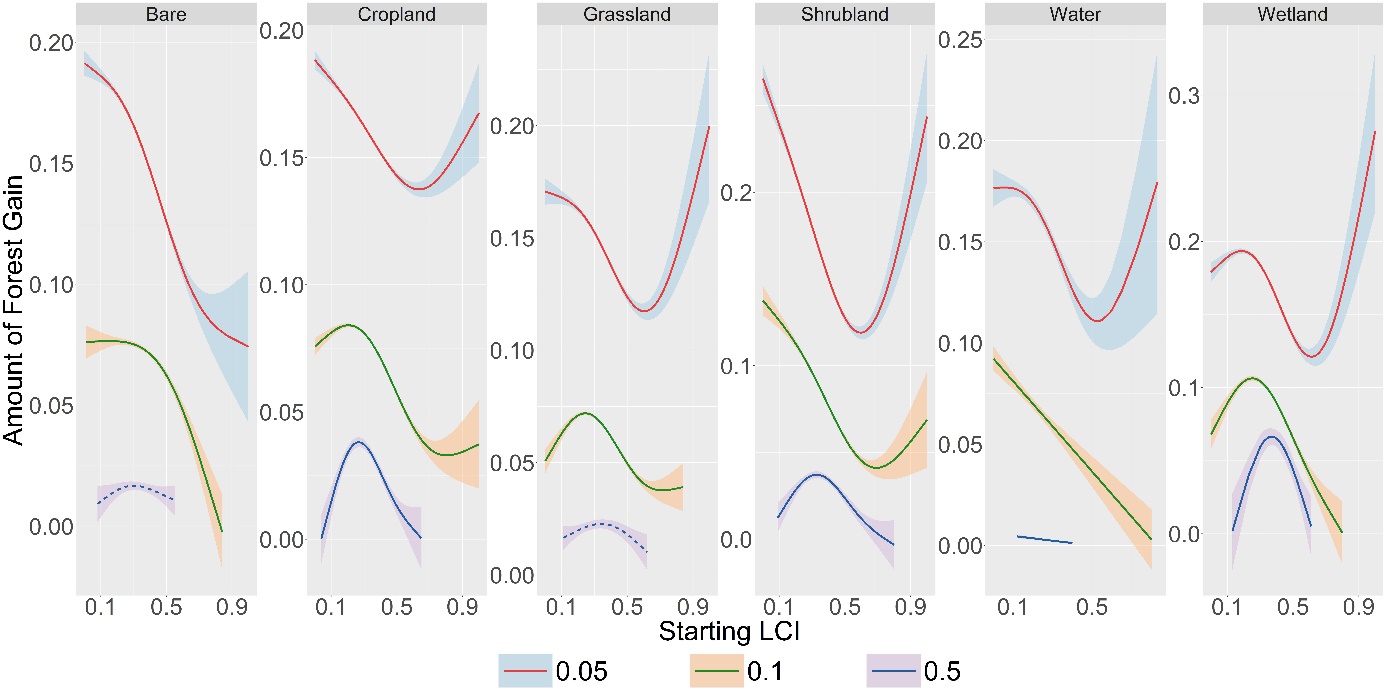
**

Figure 7: GAMs for the mean effect of the starting configuration on the mean amount of forest gain for the 0.05, 0.1 and 0.5 degree-decimal resolutions (which are equivalent to the 3, 6 and 30 arc-minute resolutions, respectively)

| **Table 7**: Multi-Scale GAM Statistics for Effects of Starting Configuration on Mean Amount of Forest Gain | | | | | | | | |
| --- | --- | --- | --- | --- | --- | --- | --- | --- |
| **Grid Size** | **Land-Cover** | **Number of Grids** | **EDF** | **Deviance** | **Fitted Values: Minimum** | **Fitted Values: Maximum** | **Mean of the Real Rate of Change** | **Mean of Absolute Rate of Change** |
| 3 arc-minute | Bare | 27019 | 2.713 | 0.864 | 1 | 0 | -0.142 | 0.142 |
| 3 arc-minute | Cropland | 84722 | 2.79 | 0.771 | 0.63 | 0 | -0.084 | 0.084 |
| 3 arc-minute | Grassland | 30413 | 2.878 | 0.72 | 0.6 | 1 | -0.089 | 0.089 |
| 3 arc-minute | Shrubland | 71533 | 2.915 | 0.886 | 0.6 | 0 | -0.244 | 0.244 |
| 3 arc-minute | Water | 3014 | 2.687 | 0.435 | 0.53 | 0.83 | -0.11 | 0.121 |
| 3 arc-minute | Wetland | 34621 | 2.929 | 0.761 | 0.61 | 1 | -0.096 | 0.144 |
| 6 arc-minute | Bare | 8877 | 2.636 | 0.473 | 0.84 | 0.15 | -0.048 | 0.049 |
| 6 arc-minute | Cropland | 26413 | 2.902 | 0.841 | 0.8 | 0.2 | -0.053 | 0.081 |
| 6 arc-minute | Grassland | 11712 | 2.909 | 0.738 | 0.71 | 0.24 | -0.017 | 0.087 |
| 6 arc-minute | Shrubland | 26185 | 2.796 | 0.852 | 0.69 | 0 | -0.154 | 0.154 |
| 6 arc-minute | Water | 719 | 1 | 0.283 | 0.8 | 0 | -0.112 | 0.112 |
| 6 arc-minute | Wetland | 7852 | 2.84 | 0.734 | 0.8 | 0.25 | -0.05 | 0.172 |
| 30 arc-minute | Bare | 295 | 1.77 | 0.061 | 0.08 | 0.3 | -0.001 | 0.026 |
| 30 arc-minute | Cropland | 381 | 2.784 | 0.462 | 0.04 | 0.27 | -0.007 | 0.122 |
| 30 arc-minute | Grassland | 237 | 1.941 | 0.081 | 0.62 | 0.34 | -0.006 | 0.033 |
| 30 arc-minute | Shrubland | 677 | 2.685 | 0.331 | 0.8 | 0.33 | -0.004 | 0.097 |
| 30 arc-minute | Water | 28 | 1 | 0.239 | 0.4 | 0.12 | -0.012 | 0.012 |
| 30 arc-minute | Wetland | 76 | 2.404 | 0.363 | 0.13 | 0.37 | -0.074 | 0.204 |
| EDF = Effective Degrees of Freedom; Deviance = The proportion of the null deviance explained by the model | | | | | | | | |

**Multi-Scale GAM Statistics for the Effect of Starting Configuration on the Mean Rate of Forest Gain**

**
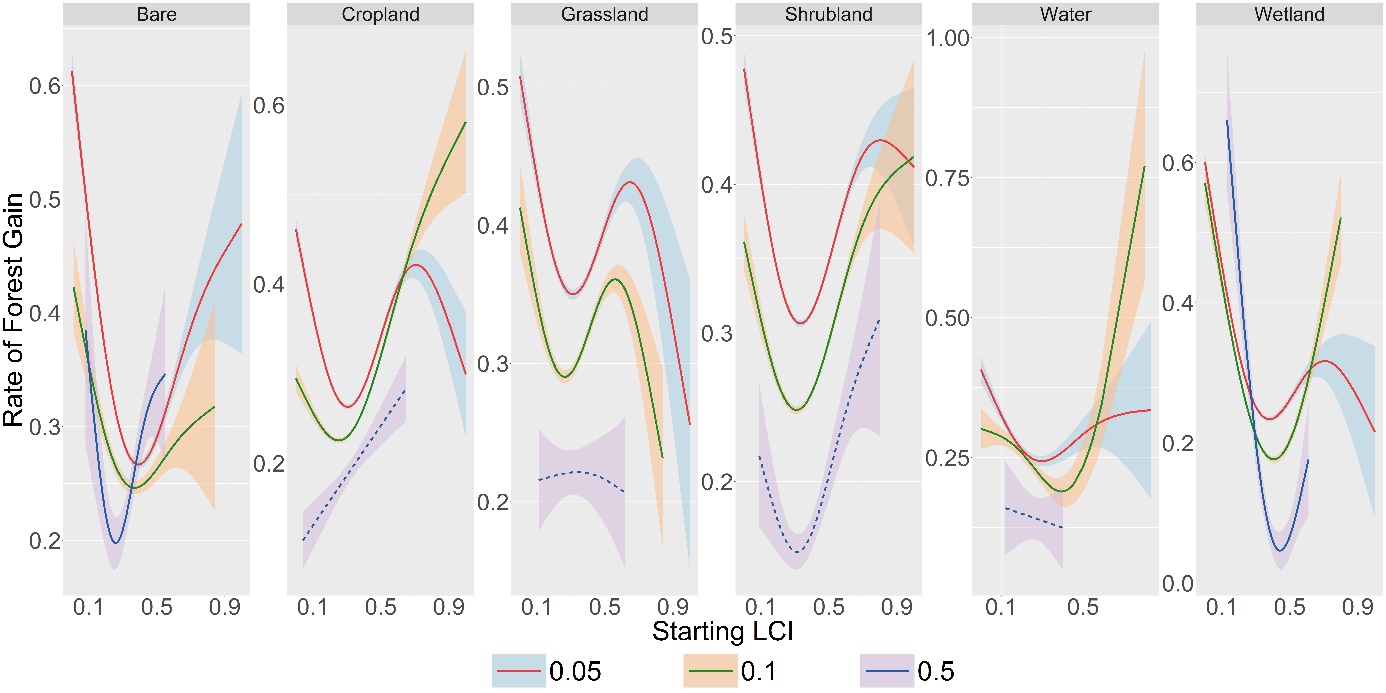
**

Figure 8: GAMs for the mean effect of the starting configuration on the mean rate of forest gain for the 0.05, 0.1 and 0.5 degree-decimal resolutions (which are equivalent to the 3, 6 and 30 arc-minute resolutions, respectively)

| **Table 8**: Multi-Scale GAM Statistics for Effects of Starting Configuration on Mean Rate of Forest Gain | | | | | | | | |
| --- | --- | --- | --- | --- | --- | --- | --- | --- |
| **Grid Size** | **Land-Cover** | **Number of Grids** | **EDF** | **Deviance** | **Fitted Values: Minimum** | **Fitted Values: Maximum** | **Mean of the Real Rate of Change** | **Mean of Absolute Rate of Change** |
| 3 arc-minute | Bare | 27019 | 2.912 | 0.832 | 0.39 | 0 | -0.458 | 0.696 |
| 3 arc-minute | Cropland | 84722 | 2.97 | 0.771 | 0.3 | 0 | -0.107 | 0.554 |
| 3 arc-minute | Grassland | 30413 | 2.903 | 0.473 | 1 | 0 | -0.135 | 0.392 |
| 3 arc-minute | Shrubland | 71533 | 2.956 | 0.75 | 0.33 | 0 | -0.147 | 0.425 |
| 3 arc-minute | Water | 3014 | 2.547 | 0.391 | 0.3 | 0 | -0.171 | 0.403 |
| 3 arc-minute | Wetland | 34621 | 2.931 | 0.881 | 1 | 0 | -0.503 | 0.721 |
| 6 arc-minute | Bare | 8877 | 2.544 | 0.226 | 0.37 | 0.01 | -0.236 | 0.363 |
| 6 arc-minute | Cropland | 26413 | 2.877 | 0.793 | 0.25 | 1 | 0.156 | 0.388 |
| 6 arc-minute | Grassland | 11712 | 2.884 | 0.366 | 0.84 | 0 | -0.092 | 0.327 |
| 6 arc-minute | Shrubland | 26185 | 2.884 | 0.665 | 0.31 | 1 | -0.04 | 0.337 |
| 6 arc-minute | Water | 719 | 2.743 | 0.288 | 0.39 | 0.8 | -0.103 | 0.344 |
| 6 arc-minute | Wetland | 7852 | 2.924 | 0.812 | 0.41 | 0 | -0.465 | 0.824 |
| 30 arc-minute | Bare | 295 | 2.494 | 0.231 | 0.26 | 0.08 | 0.08 | 0.64 |
| 30 arc-minute | Cropland | 381 | 1 | 0.118 | 0.04 | 0.65 | 0.279 | 0.279 |
| 30 arc-minute | Grassland | 237 | 1.166 | 0.008 | 0.62 | 0.34 | -0.01 | 0.036 |
| 30 arc-minute | Shrubland | 677 | 2.31 | 0.162 | 0.31 | 0.8 | 0.069 | 0.31 |
| 30 arc-minute | Water | 28 | 1 | 0.003 | 0.4 | 0.12 | -0.125 | 0.125 |
| 30 arc-minute | Wetland | 76 | 2.598 | 0.607 | 0.44 | 0.13 | -0.632 | 1.119 |
| EDF = Effective Degrees of Freedom; Deviance = The proportion of the null deviance explained by the model | | | | | | | | |

**Appendix V**

Here, it is important to note that our analysis focuses on summary statistics of the LC change process over the entire timeseries (from 1992 to 2020). To address this concern, it is thus important to consider how the uncertainties in LC classification propagate into uncertainties regarding these summary statistics. Specifically, the summary statistics that form the two response variables of our study are:

1. Net Forest Change
2. Rate of Forest Change

Summarizing the analysis (which we present in more detail below), we first find that estimates of net forest change are robust to classification uncertainty for all LC transitions for which sufficient reference data is available. For forest-wetland and forest-settlement transitions, insufficient reference data is currently available to reliably assess their classification uncertainty, as we now acknowledge in the revised version of the manuscript (see details below).

Second, the accumulation of classification uncertainty as it propagates through the time-series, assumes the temporal independence of classification errors. Previous studies, however, suggest that classification errors tend to persist over time (Tsutsumida and Comber, 2015), which would drastically reduce their impact on estimates of extents and rates of LC change. Analyzing the frequency of landscape stability in our dataset corroborates this notion of spatio-temporal autocorrelation in classification errors. This implies that classification uncertainty does not distort our assessment of forest change dynamics in any major way, providing validation for the results and conclusions mentioned in the study. In the following text, we provide details regarding the tests and analysis that were done to evaluate the effects of classification uncertainty.

**Assessing the Impact of Classification Uncertainty on the Net Forest Change:**

Our rationale for assessing the impact of classification uncertainty on our estimates of net forest change was as follows. The reported uncertainties in LC classification from the study by Defourny et al. (2021) can be used to assess, for one point in time, the uncertainty in the estimate of forest extent and the type of LC it transitions to. This procedure is explained in Step 1 below. Subsequently, we aimed to assess how these uncertainties in forest extent estimates compared to the inferred magnitudes of forest change over the time-series (from 1990 to 2020). If uncertainties are relatively small compared to the magnitudes of net forest change, then conclusions about the sign of net forest change can be robustly drawn. However, if uncertainties become larger, this will impact the ability of drawing robust conclusions about the direction of forest cover change. In the case of inferred forest loss, a particular concern is that there was an overestimation of forest cover at the beginning of the time period, and an underestimation of forest cover at the end of the time period. In that case, even a stable landscape, or one undergoing an increase in forest cover, could erroneously be characterized as a landscape with forest loss, and be treated in our analysis accordingly. In step 2 below, we therefore assess for what proportion of our grids this situation may have occurred. For these analyses, we restricted our scope to grids that showed net forest loss only (n=246,174) and performed the following steps:

1. **Modeling the Estimation Uncertainty of possible Forest Pixel Amounts (0-324):**
   1. We used the confusion matrices for the 2016-2020 CCI-LC classification maps (Defourny et al., 2021) and aggregated the LCCS legend values to the IPCC LC categories (see Table 1 and Table 2).

| **Table 1: Legend Mapping from LCCS to IPCC Categories** | | |
| --- | --- | --- |
| **IPCC Classes** | **LCCS Legend Used in CCI-LC Maps*** | |
|  | **Value** | **Label** |
| Cropland | 10,11,12 | Cropland, Rainfed |
|  | 20 | Irrigated Cropland |
|  | 30 | Veg-Crop Mosaic Cropland (>50%) |
|  | 40 | Veg-Crop Mosaic Cropland (<50%) |
| Forest | 50 | Tree Cover, broadleaved, evergreen |
|  | 60,61,62 | Tree Cover, broadleaved, deciduous |
|  | 70,71,72 | Tree Cover, needle-leaved, evergreen |
|  | 80,81,82 | Tree Cover, needle-leaved, deciduous |
|  | 90 | Tree Cover, mixed leaf type (broadleaved and needle-leaved) |
|  | 100 | Mosaic Tree and Shrub (>50%) |
|  | 160 | Tree Cover, flooded, fresh or brackish water |
|  | 170 | Tree cover, flooded, saline water |
| Grassland | 110 | Mosaic Tree and Shrub (<50%) |
|  | 130 | Grassland |
| Shrubland | 120,121,122 | Shrubland |
| Bare | 140 | Lichens and Mosses |
|  | 150,151,152 | Sparse Vegetation |
|  | 200, 201, 202 | Bare Areas |
| Settlement | 190 | Urban Areas |
| Wetland | 180 | Shrub or Herbaceous cover, flooded, fresh/saline/brackish water |
| Water | 210 | Water Bodies |
| *The class ‘Bare’ also includes ‘Sparse Vegetation’. | | |

| **Table 2: Confusion Matrix for the 2016 CCI-LC Map** | | | | | | | | |
| --- | --- | --- | --- | --- | --- | --- | --- | --- |
|  | Cropland | Forest | Grassland | Shrubland | Bare | Settlement | Wetland | Water |
| Cropland | 202 | 17 | 24 | 10 | 3 | 1 | 0 | 0 |
| Forest | 6 | 482 | 9 | 26 | 10 | 2 | 0 | 0 |
| Grassland | 11 | 1 | 67 | 20 | 38 | 2 | 1 | 1 |
| Shrubland | 8 | 27 | 21 | 105 | 9 | 0 | 0 | 0 |
| Bare | 7 | 0 | 25 | 24 | 116 | 1 | 0 | 0 |
| Settlement | 0 | 1 | 6 | 0 | 1 | 4 | 0 | 0 |
| Wetland | 0 | 1 | 0 | 0 | 0 | 0 | 3 | 0 |
| Water | 1 | 1 | 0 | 0 | 0 | 0 | 0 | 57 |

- 1. For each LC transition and for each year (2016-2020), we extracted relevant values and constructed a confusion matrix. For instance, the Forest to Cropland transitions’ confusion matrix (based on the 2016 map) is shown in Table 3 below. For each LC transition, therefore, we had a set of five 2-by-2 matrices.

| **Table 3: Confusion Matrix for the Forest to Cropland transition (2016 Map)** | | | |
| --- | --- | --- | --- |
|  | **Reference** | | |
| **Map** |  | Forest | Cropland |
|  | Forest | 482 | 6 |
|  | Cropland | 17 | 202 |

- 1. We constructed uniform distributions using the min/max values from the set of five values for each of the 4 matrix positions. Thereafter, we simulated confusion matrices from these uniform distributions, specific to each LC transition.
  2. We estimated the true forest pixel amount, as well as its standard error, based on the method described by Olofsson et al. (2013), where

$$Estimated Forest Amount=324*\sum_{i=1}^{2} W_{i}\frac{n_{ij}}{n_{i}}$$

Here, $W_{i}$ are the proportions of the forest and transition LC in the grid cell (we assume these are complementary, where number of transition LC pixels equals 324 – number of forest pixels); $n_{ij}$ is the number of pixels that are mapped as forest but are, in fact, the transition LC; and $n_{i}$ are the sum of all pixels mapped as Forest.

- 1. We generate an estimated forest amount lookup table for all observed forest counts (0–324 pixels) for each transition LC. We repeated this process 1000 times per forest count to capture the range of possible estimates. In Figure 1, we show the median estimate for each observed forest count, as well as the 95% confidence interval using the median standard error, for each transition LC.


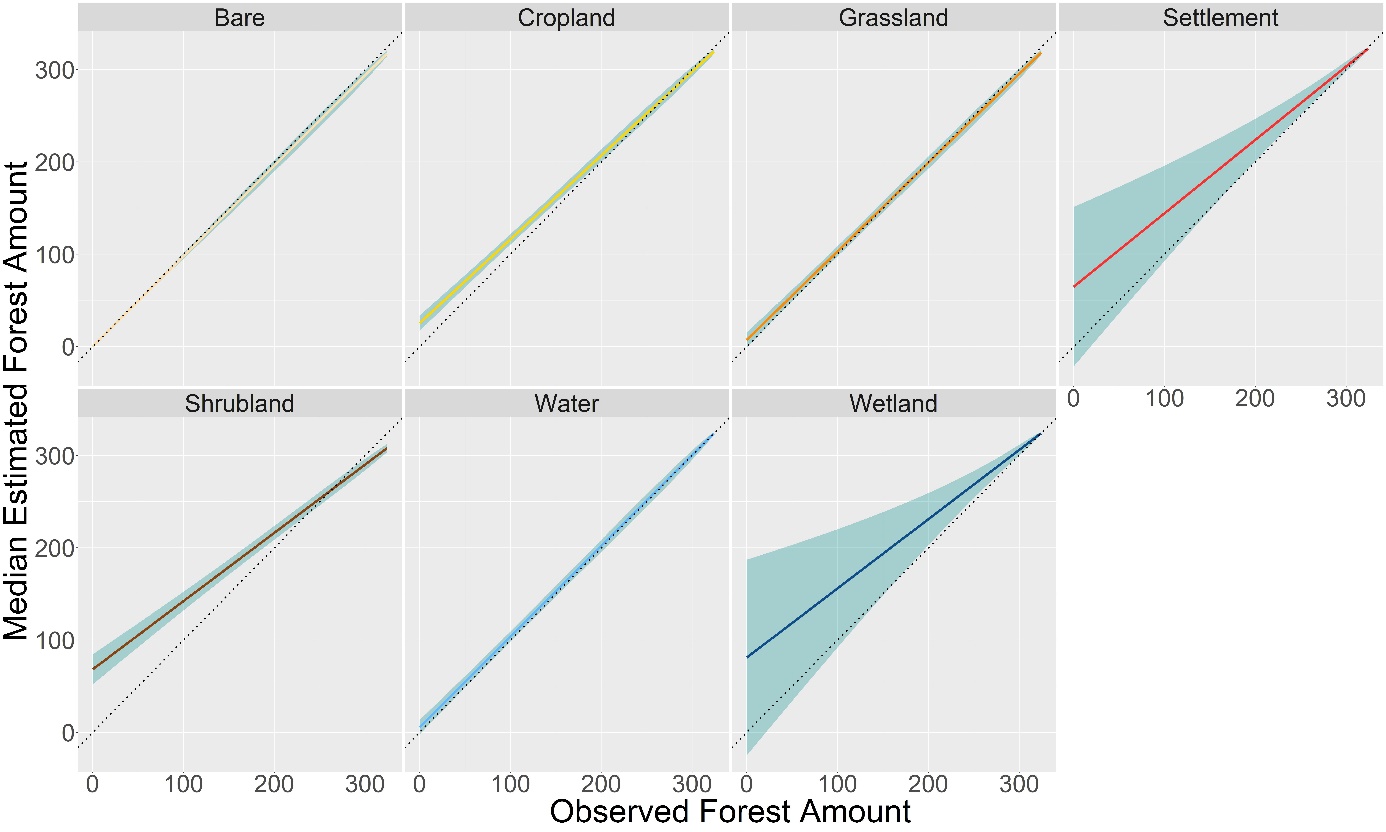


Figure 1: Median Estimated Forest Amount for each Observed Forest Amount (0-324), along with the 95% confidence interval of the estimate (shown with a blue ribbon). The y=x line (dotted) represents perfect agreement between the observed and estimated forest amount.

Here, the wide confidence intervals around the estimated forest amount for the settlement and wetland transitions reflects the limited reference data, as the classification error estimates for the settlement and wetland classes were derived from 4 and 10 samples respectively. Such limited reference data introduces high levels of uncertainty in the estimated forest amount. In contrast, cropland uncertainty estimates were derived from >200 samples which makes them more stable with narrower confidence intervals.

1. **Assessing the Robustness of Observed Net Forest Loss to Classification Uncertainty:**
   1. For each grid, we extracted the forest amount at the start and end points of the interval of forest change.
   2. For the observed starting and ending amounts, we extracted their corresponding 2.5^th^ and 97.5^th^ percentile estimates of forest extent using the lookup table (described in Step 1.5. above).
   3. We then compare if the Forest Start Estimate > Forest End Estimate. If ‘True’, then we flag that grid (and its corresponding estimate of net forest change) as robust to the classification uncertainty. If not, then the grid is flagged as unreliable.
   4. We progressively reduce the bounds of the starting and ending forest amount estimates being compared (for example, comparing the 2.5^th^ vs. 97.5^th^ percentile, then the 5^th^ vs. 95^th^, and so on) to determine how many grids remain robust under less stringent uncertainty thresholds.
   5. Further, it is important to note that when testing robustness using, for example, the 2.5^th^ and 97.5^th^ percentile of starting and ending forest amounts, the combined probability of both extremes occurring simultaneously

***P***(2.5th percentile of Forest Start Estimate ∩ 97.5th percentile of Forest End Estimate) = ***P***(2.5th percentile of Forest Start Estimate) x P(97.5th percentile of Forest End Estimate) = 0.025 x 0.025 = 0.000625.

This represents an extremely conservative (99.9375% confidence) test. For instance, the 95% confidence interval for the joint uncertainty would be better approximated when using the 22^nd^ and the 78^th^ percentile estimates for the Forest Start and End estimates, since 0.22 x 0.22 = 0.0484 ~ 0.05.

- 1. We calculate the percentage of grids per transition LC that were robust to the varying levels of classification uncertainty (Figure 2).


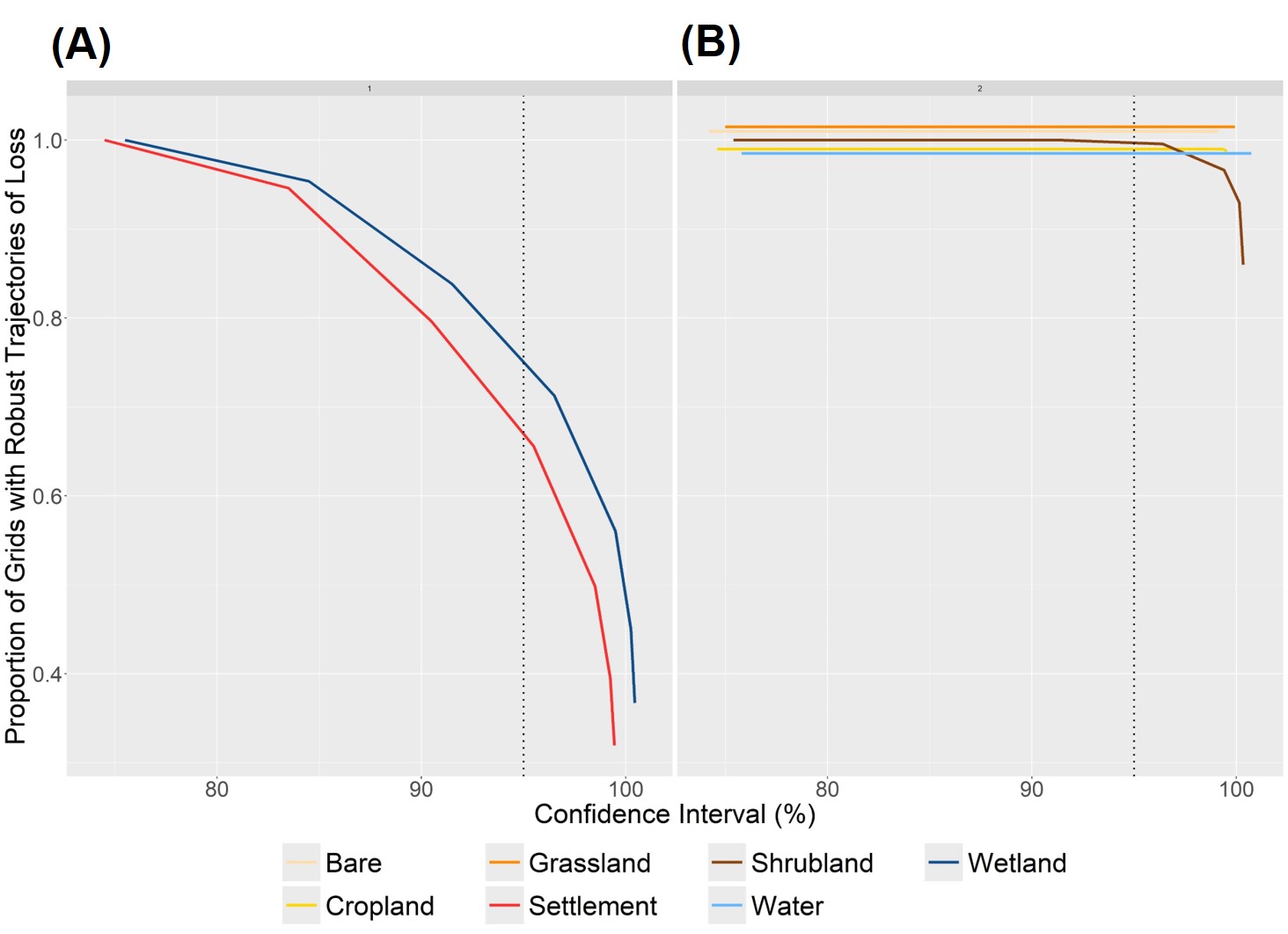


Figure 2: Proportion of grids for the settlement and wetland transitions (A) and for all other transition LCs with statistically robust net forest loss estimates assessed under varying confidence intervals. In Plot B, the percentage of robust estimates are equal to 1 but have been jittered to enable a discrimination between the different LC transitions. The dotted vertical line indicates the 95% confidence interval.

Here, we can see that all our estimates of net forest loss (~100%) for the cropland, grassland, shrubland, bare and water transitions are robust to classification uncertainty at the 95% confidence interval. For grids of wetland and settlement transitions, the current lack of reference data, means that we cannot reliably assess classification errors. Hence, this lack of information also propagates into estimates of forest transitions into these classes being characterized by high uncertainty. While this analysis invites caution in the interpretation of the results associated with the settlement and wetland transitions, it also serves to underscore the need for a robust sampling design that can ensure the collection of adequate reference data for all LC classes.

**Assessing the Impact of Classification Uncertainty on the Rate of Forest Change:**

Assuming the temporal independence of classification errors, the probability of accurately capturing the spatial-temporal trajectory of LC change decreases with increasing length of the time-series. However, previous studies have demonstrated that classification errors are typically spatially auto-correlated and exhibit temporal persistence (Tsutsumida and Comber, 2015). If classification errors are spatially and temporally autocorrelated, this would imply that the impact of classification on LC dynamics would be much more subdued.

While we do not have time-series reference data to assess spatio-autocorrelations of classification errors directly, we can assess the likelihood of the opposite case, i.e. with classification errors being independent in time and space. Specifically, in this latter case, it would be rather improbable to see the exact same landscape configuration at two consecutive timepoints. A useful starting point for this analysis is thus to assess how often landscape stability was observed within our analyzed data. Specifically, we tabulated the number of stable forest segments across our persistent forest loss dataset (n=246,174). A segment here refers to successive time-steps of the forest cover time-series. Because we are working with a 29-year time-series, we have a total of 28 segments possible within the time-series. Figure 3 shows the frequency distribution of the number of stable forest segments, i.e. segments for which forest cover appeared to remain constant and stable in the grid cell.


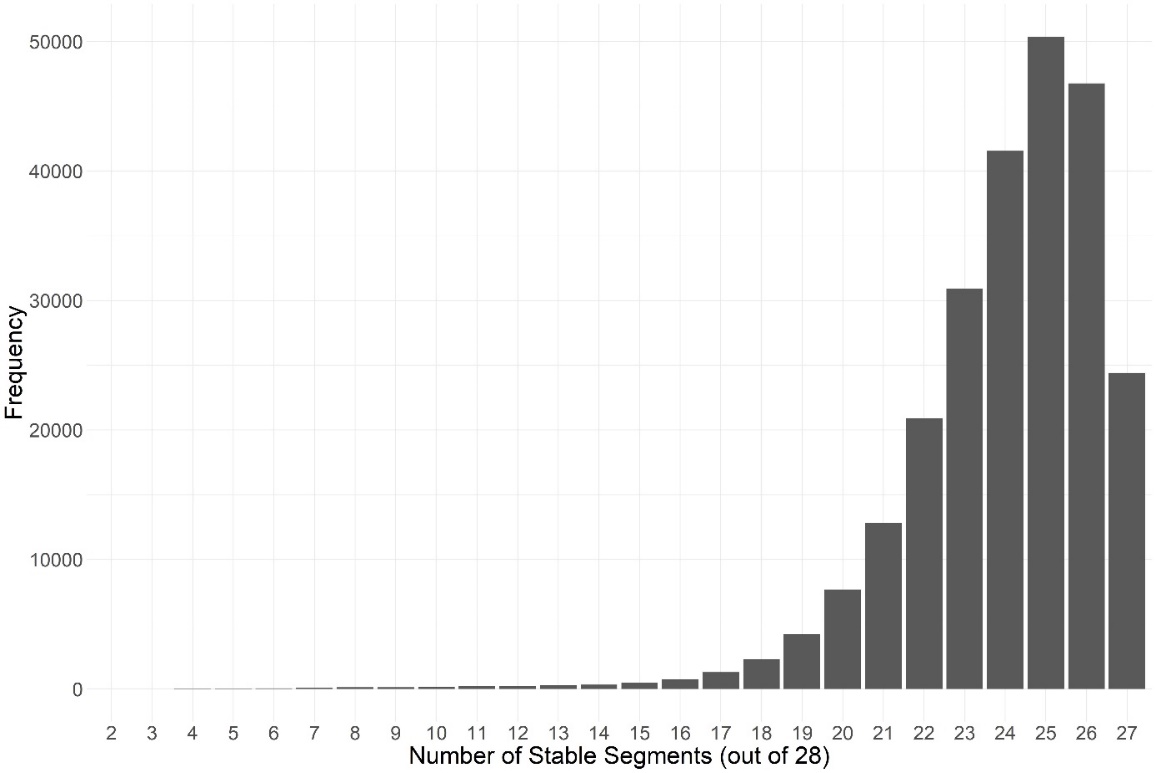


Figure 3: Frequency distribution of the number of stable forest segments per grid for all persistent forest loss grids (n=246,174)

Here, we can clearly see that periods of forest cover stability are extremely common in the forest cover time-series. This illustrates the prevalence of periods of time when forest cover is held constant in the landscape. Following the above line of reasoning, this suggests that spatio-temporal independence of classification errors is quite unlikely.

To test this notion more formally, we used the propagated uncertainties of LC transitions as described in the previous section. We used these uncertainties to simulate landscape dynamics, under the assumption of spatio-temporal independence of classification errors. The resulting frequency of stable segments were then compared to the number of stable segments observed in the data. Specifically, this procedure consisted of the following steps:

1. We randomly sampled 10,000 time-series of persistent forest loss, where forests have transitioned to croplands. We chose croplands, because of their prevalence, but also because their standard errors, as compared with shrublands, wetlands or settlements, are smaller and therefore less prone to large fluctuations (see Figure 1 above).
2. For each time-series, we estimated the median forest amount and the standard errors using the look-up tables (described earlier). We used the ‘rtruncnorm’ function of the ‘truncnorm’ packages to generate simulated time-series of forest cover by randomly applying error corrections drawn from a standard normal distribution with median and standard deviation equal to the values extracted from the look up tables (Mersmann et al., 2025). As this procedure was repeated for each timestep, it considered classification errors that were spatio-temporally independent.
3. For each simulated forest cover time-series, we calculated the number of segments where forest cover was stable.
4. For each one of the 10000 trajectories, Steps 2 and 3 were repeated 1000 times.

We calculated the mean number of stable segments per time-series from these 1000 replicate simulations, and compared this to the observed number of stable segments. The mean of the simulated stable forest segments was plotted against the observed number of stable forest segments for the 10,000 time-series and is shown in Figure 4.


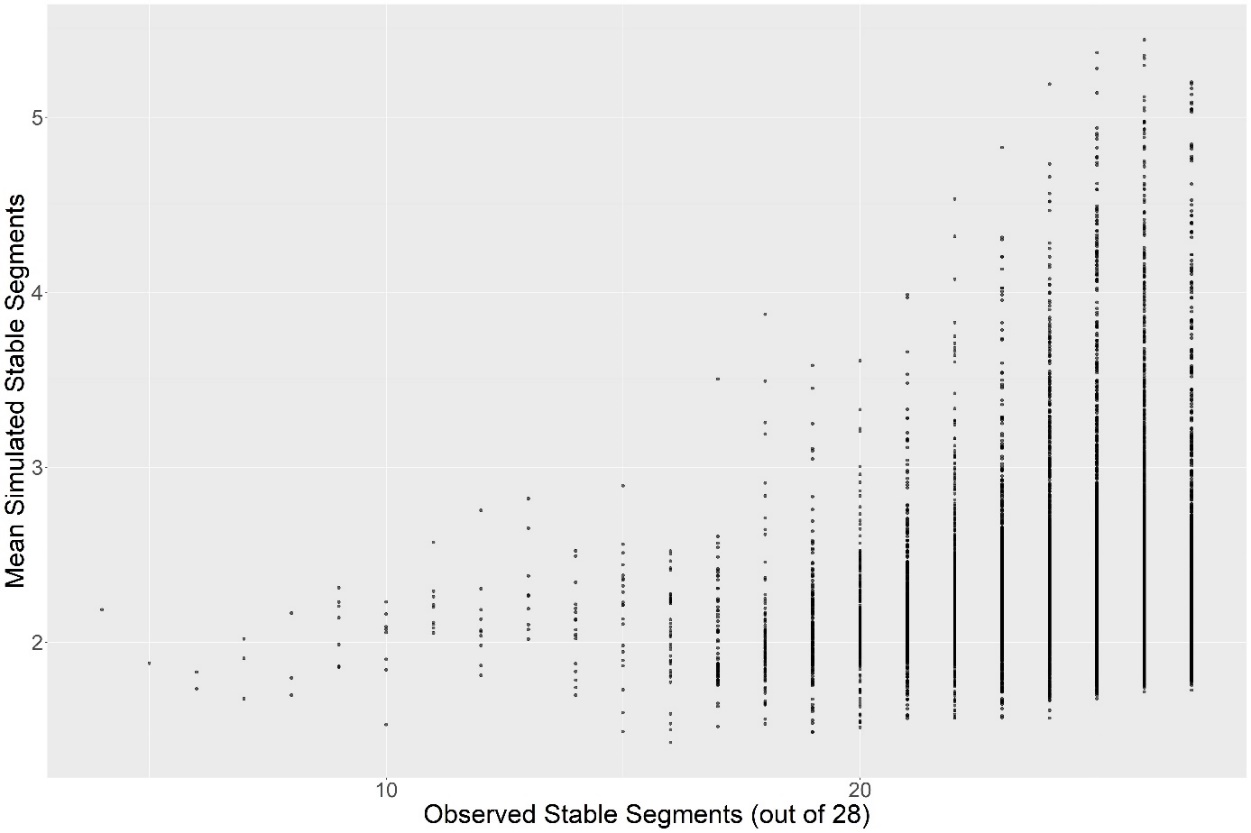


Figure 4: Plot showing the mean of simulated stable forest segments against the observed number of stable forest segments for 10,000 trajectories of persistent forest loss.

The different ranges of the x- and y-axes in Figure 4 clearly indicate that the number of stable segments under the assumption of spatio-temporally independent classification errors would be much lower than the numbers observed in the dataset. Specifically, for observed trajectories of forest loss, observed number of stable forest segments (mean = 24.00) far exceeds those generated by applying forest amount error-corrections independently across the time-series (mean = 2.35). This strongly suggests that classification errors are temporally autocorrelated. In summary, our trajectories of forest loss are characterized by alternating years of stable and declining amounts of forest cover. The above analysis shows that the combination of these two phases is extremely unlikely to be the result of classification errors, that are spatially and temporally independent. While each grid is clearly subject to classification errors, if these errors are spatially and temporally autocorrelated, their impact on the summary variables of interest, namely the net amount and rate of cover change, would be much more subdued. Hence, from this analysis, we conclude that our estimations of these two response variables are robust to classification errors in LC types.

**References:**

Defourny, P., Lamarche, M., Marissiaux, Brockmann, C., Boettcher, M., Kirches (2021). Product User Guide and Specification ICDR Land Cover 2016-2020 <https://community.esri.com/ccqpr47374/attachments/ccqpr47374/arcgis-pro-questions/23785/1/D3.3.12-v1.1_PUGS_ICDR_LC_v2.1.x_PRODUCTS_v1.1.1_APPROVED_Ver1.pdf>

Mersmann O, Trautmann H, Steuer D, Bornkamp B (2025). truncnorm: Truncated Normal Distribution. R package version 1.0-9, <https://github.com/olafmersmann/truncnorm>.

Olofsson, P., Foody, G. M., Stehman, S. V, & Woodcock, C. E. (2013). Making better use of accuracy data in land change studies: Estimating accuracy and area and quantifying uncertainty using stratified estimation. *Remote Sensing of Environment*, *129*, 122–131. https://doi.org/10.1016/j.rse.2012.10.031

Tsutsumida, N., & Comber, A. J. (2015). Measures of spatio-temporal accuracy for time series land cover data. *International Journal of Applied Earth Observation and Geoinformation, 41, 46–55*.
